# Action on diabetic macular oedema: achieving optimal patient management in treating visual impairment due to diabetic eye disease

**DOI:** 10.1038/eye.2017.53

**Published:** 2017-05-11

**Authors:** R Gale, P H Scanlon, M Evans, F Ghanchi, Y Yang, G Silvestri, M Freeman, A Maisey, J Napier

**Affiliations:** 1 ^1^The Action on DMO group, UK; 2 ^2^The York Hospital, York, UK; 3 ^3^Gloucestershire Hospitals NHS Foundation Trust, Cheltenham, UK; 4 ^4^University Hospital, Llandough, Cardiff, UK; 5 ^5^Bradford Teaching Hospitals, Bradford, UK; 6 ^6^The Royal Wolverhampton NHS Trust, Wolverhampton, UK; 7 ^7^Belfast Health & Social Care Trust, Belfast, UK; 8 ^8^Royal Hallamshire Hospital, Sheffield, UK; 9 ^9^Cardiff and Vale University Health Board, University Hospital of Wales, Cardiff, UK; 10 ^10^Bayer, Reading, UK

## Abstract

This paper identifies best practice recommendations for managing diabetes and sight-threatening diabetic eye disease. The authors provide an update for ophthalmologists and allied healthcare professionals on key aspects of diabetes management, supported by a review of the pertinent literature, and recommend practice principles for optimal patient management in treating visual impairment due to diabetic eye disease. In people with diabetes, early optimal glycaemic control reduces the long-term risk of both microvascular and macrovascular complications. The authors propose more can and should be done to maximise metabolic control, promote appropriate behavioural modifications and encourage timely treatment intensification when indicated to ameliorate diabetes-related complications. All people with diabetes should be screened for sight-threatening diabetic retinopathy promptly and regularly. It is shown that attitudes towards treatment adherence in diabetic macular oedema appear to mirror patients' views and health behaviours towards the management of their own diabetes. Awareness of diabetic macular oedema remains low among people with diabetes, who need access to education early in their disease about how to manage their diabetes to delay progression and possibly avoid eye-related complications. Ophthalmologists and allied healthcare professionals play a vital role in multidisciplinary diabetes management and establishment of dedicated diabetic macular oedema clinics is proposed. A broader understanding of the role of the diabetes specialist nurse may strengthen the case for comprehensive integrated care in ophthalmic practice. The recommendations are based on round table presentations and discussions held in London, UK, September 2016.

## Introduction

Diabetes has been estimated to affect over 8% of the world's population and, as a significant cause of mortality and major morbidity, is now posing one of the greatest challenges for our healthcare systems.^1, 2^ The global prevalence of diabetes is set to double over the next 20 years, with the vast majority of this increase related to type 2 diabetes.^1, 2^ As such, type 2 diabetes is increasingly recognised as a disease pandemic that is arguably one of the largest global health emergencies of the 21st century.^2^

There is therefore an increasing need for knowledge, skills, resources, and infrastructure to deliver the comprehensive healthcare needed to meet the complex demands of this progressive condition. Health policy and interventions should focus on diabetes prevention and early detection, including diabetic eye screening, as well as rehabilitation and long-term care of patients affected by wide-ranging complications of diabetes, such as end organ failure, blindness or amputation.

Healthcare professionals often work in isolation to deliver highly specialised care efficiently and effectively for the individual patient with diabetes and its effects on multiple organ systems. It is not uncommon for a patient to be making frequent visits to community clinics and different hospital clinics to see a variety of specialists and allied healthcare professionals, with seemingly little opportunity for coordination of this complex health management programme between the wider team involved. In a field that is so diverse and rapidly changing, healthcare professionals of all specialties need to be aware of developments across all aspects of diabetes management.

In this article, the authors provide an update for ophthalmologists and allied healthcare professionals on diabetes prevalence, the perspective of the diabetologist on what an ophthalmologist needs to know about diabetes, including developments and challenges in diabetic retinopathy screening. In addition, the paper discusses qualitative research assessment of treatment adherence and patient experience, the role of the diabetes specialist nurse (DSN) and practice principles for the management of people with diabetic eye disease. Summaries with concise ‘take home' messages from each topic are provided to enable the reader to easily focus on or refer to specific issues or areas of interest.

### Diabetes is an escalating global health challenge

The World Health Organization (WHO) estimates that the global age-standardised adult prevalence of diabetes has nearly doubled since 1980, rising from a prevalence rate of 4.7% in 1980 to 8.5% of the adult population in 2014 and reflecting an increase in associated risk factors.^1^ The rise in the age-standardised adult prevalence of diabetes is compounded by population growth and ageing, with older adults accounting for a larger proportion of the population in developed countries.^3^ Type 2 diabetes, consequent upon complex pathophysiology but effectively driven by defective energy homoeostasis and closely linked with obesity (body mass index ≥30 kg/m^2^) and physical inactivity, accounts for the majority of people with diabetes worldwide.^1^

Prevalence data, estimated by the Non-Communicable Diseases (NCD) Risk Factor Collaboration, were pooled from population-based studies that had collected data on diabetes through measurement of its biomarkers to arrive at estimated trends in diabetes prevalence since 1980.^3^ Diabetes was defined as fasting plasma glucose (FPG) ≥7.0 mmol/L; a history of diagnosis with diabetes; or use of insulin or oral hypoglycaemic drugs. The International Diabetes Federation (IDF) estimates there were 415 million adults aged 20–79 years with diabetes worldwide in 2015, including 193 million undiagnosed (Table 1).^2^

An estimated 1.5 million deaths in 2012 were directly caused by diabetes, and another 2.2 million deaths were attributable to hyperglycaemia.^1^ Diabetes and the management of related complications accounted for 12% of total healthcare expenditure worldwide in 2015, with over 80% of this expenditure being driven by the costs of complications.^2^ The direct annual cost of diabetes in the world is estimated at US $825 billion based on diabetes prevalence projections for 2014.^3^ Three-quarters of the global expenditure on diabetes was for people aged between 50 and 79 years, reflecting a higher prevalence of diabetes and of diabetes complications in this burgeoning age group.^2^

#### Prevalence estimates of United Kingdom adult diabetes approach 1 in 10

The WHO estimates a UK diabetes prevalence rate of 7.7% in 2014.^1^ The estimated number of UK adults with diabetes in 1980 was 2.3 million, increasing to an estimated 3.8 million adults with diabetes in 2014.^3^ Approximately 90% of people with diabetes have type 2 diabetes.^4^ Table 2 shows estimates of diabetes prevalence in the adult population across the United Kingdom for 2016: 3.6 million people have diagnosed diabetes and a further 1 million with undiagnosed diabetes.^4^

**Figure 1:**
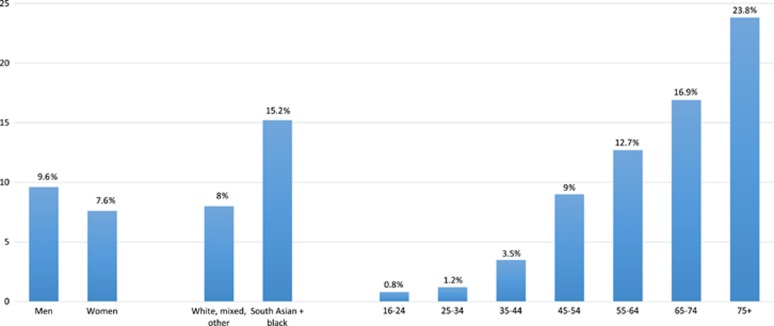
Expected diabetes prevalence (diagnosed and undiagnosed) for England in 2015 by gender, ethnicity, and age group.^5^ A Public Health England (PHE). Diabetes Prevalence Model. Public Health England, September 2016. Contains public sector information licensed under the Open Government Licence v3.0. The 2016 PHE diabetes prevalence model incorporates more up-to-date data sources and population estimates than previously published diabetes prevalence models. The previous model published in 2012 underestimated undiagnosed diabetes, suggesting that the overall prevalence estimates were probably low.

Prevalence estimates prepared by Public Health England's National Cardiovascular Intelligence Network indicate that around 9% of the adult population in England in 2015 has diabetes and this prevalence rate is projected to increase to 9.7% by 2035.^5^ Diabetes prevalence rates increase with age, from 9.0% for people aged 45–54 years to 23.8% for people aged 75 years and older (Figure 1). According to Public Health England, 2% of adults aged 16–44 years have diabetes, equating to 400 000 people and accounting for 10% of total estimated diabetes cases.^5^

Clinical commissioning groups (CCGs) with the highest estimated diabetes prevalence have high proportions of South Asian and black ethnic groups and high levels of deprivation, reflecting the well-recognised association between ethnicity and deprivation with incident diabetes risk.^6, 7^ The analysis and comparisons with the 2014/15 Quality and Outcomes Framework suggest that 76% of people with diabetes have been diagnosed and are included on General Practice (GP) registers, that is, 24% of people with diabetes are undiagnosed.

Hamer *et al*^8^ found a nearly doubling in the prevalence rate of diabetes over the 5-year period between 2003 and 2008 in Scotland, which increased from 5.2% in 2003 to 9.4% in 2008. Imkampe and Gulliford^9^ similarly identified increasing diabetes prevalence rates in United Kingdom. Prevalence of diagnosed diabetes increased in men from 3.74% in 1994 to 7.25% in 2006 and in women from 2.28 to 4.88% (prevalence based on self-reported diabetes diagnosed by a doctor). The IDF estimates a diabetes prevalence rate in Ireland of 5.3% in adults aged 20–79 years in 2015,^2^ with 171 800 adults having diagnosed diabetes

Diabetes-related health expenditure in the United Kingdom was estimated at US $13 billion in 2015.^2^ The nation is ranked one of the top ten countries for diabetes-related healthcare expenditure.^2^

#### National Diabetes Audit (United Kingdom and Wales), key findings, and recommendations

The National Diabetes Audit (NDA) provides a comprehensive overview of diabetes care in United Kingdom and Wales. The audit collects information from both primary and secondary care. Participation for the 2014–2015 NDA report was around 4700 GP practices (57%) and 99 specialist services, capturing information on 1.9 million people with diabetes, with the national participation rate lower than in previous years (70.7% national participation rate in the audit year 2012–2013).^10^

Annual care processes recommended by the National Institute for Health and Care Excellence (NICE) for all people with diabetes aged 12 years and older encompass blood tests (glycated haemoglobin (HbA1c), serum creatinine, cholesterol), blood pressure, urine albumin, foot surveillance, body mass index, and smoking history, which is the responsibility of Diabetes Care Providers.^10^ Annual digital retinal screening, also recommended by NICE, is the responsibility of National Health Service (NHS) Diabetic Eye Screening. The percentage of people with diabetes in United Kingdom and Wales receiving all eight NICE recommended care processes by Diabetes Care Providers in the audit year 2014-15 was 38.7% for people with type 1 diabetes and 58.7% for people with type 2 diabetes.^10^ Blood tests and blood pressure checks are more reliably performed than other care processes.^10^

**Figure 2:**
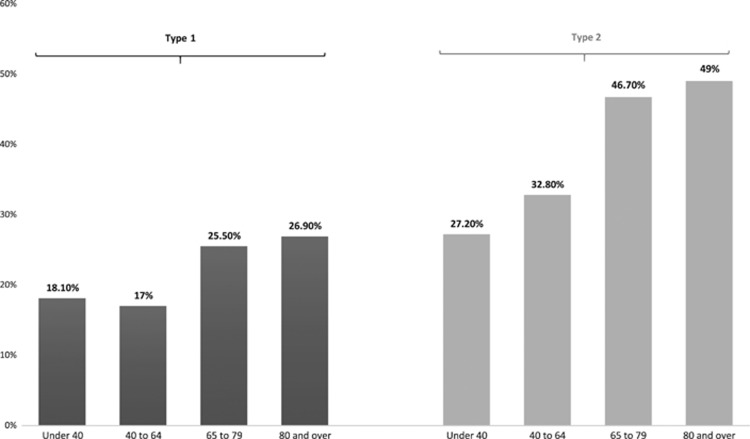
Percentage of all people with diabetes in United Kingdom and Wales achieving all three treatment targets (HbA1c ≤58 mmol/mol (7.5%), blood pressure ≤140/80 mm Hg, and cholesterol <5 mmol/l) by diabetes type and age group, 2014–2015.^10^ National Diabetes Audit 2013–2014 and 2014–2015. Report 1: Care Processes and Treatment Targets. Published 28 January 2016 by the Health and Social Care Information Centre (HSCIC), also known as NHS Digital. Contains public sector information licensed under the Open Government Licence v3.0.

Encouraging trends of improvement in blood pressure control for people with type 1 and type 2 diabetes and glucose control for type 1 diabetes were observed in the NDA audit report 2013–2015.^10^ However, people aged under 40 are much less likely to receive their care processes and those under 65 with either type 1 or type 2 diabetes are much less likely to achieve recommended treatment targets for HbA1c, blood pressure and cholesterol compared with those aged 65 years and older (Figure 2).^10^ There remain appreciable variations in care process completion and treatment target achievement between practices, between specialist services and between CCGs and Local Health Boards (LHBs).^10^

Furthermore, 75% of people newly diagnosed with diabetes were offered structured education in United Kingdom and Wales within 1 year of diagnosis in the audit year 2014–2015, compared with only 10.3% in the audit year 2010–2011. However, there is a large disparity between structured education offers and attendances: records of attending structured education have increased only modestly to 5.3% in 2014–2015 compared with 3.4% in 2012–2013.^10^

*Summary:* Diabetes is an escalating global health challenge.

- Diabetes is an escalating global health challenge; global diabetes prevalence has doubled since 1980.
- Type 2 diabetes, consequent upon complex pathophysiology but effectively driven by defective energy homoeostasis and closely linked with obesity and physical inactivity, accounts for the majority of people with diabetes worldwide.
- Recent prevalence estimates of 2015 indicate that around 9% of the adult population in United Kingdom has diabetes (3.8 million people, of which 940 000 have diabetes that is undiagnosed).
- Implementation of effective measures may help prevent type 2 diabetes and improve management to reduce diabetes-related complications and premature deaths; obesity and overweight are the strongest risk factors for type 2 diabetes.
- The National Diabetes Audit shows that those aged under 65 with either type 1 or type 2 diabetes are much less likely to achieve recommended treatment targets for HbA1c, blood pressure, and cholesterol compared with those aged 65 and older.

### The diabetologists' perspective: what an ophthalmologist and other healthcare professionals need to know about diabetes

Diabetes can cause major health problems related to microvascular and macrovascular complications. Diabetes was ranked the seventh leading cause of disability worldwide in 2013, increasing by more than two ranks compared with the same global evaluation of leading causes of global years lived with disability in 1990.^11^ Chronic hyperglycaemia can seriously affect the cardiovascular system, eyes, kidneys, and nerves, and in almost all high-income countries, diabetes is a major cause of cardiovascular disease, blindness, kidney failure and lower-limb amputation.^2^

Report 2 from the National Diabetes Audit for 2012–2013 presents findings on diabetes complications and mortality in United Kingdom and Wales.^12^ It shows that people with diabetes are considerably more likely than people without diabetes to be admitted to hospital with angina, myocardial infarction, heart failure and stroke, and are at far greater risk for a major amputation and renal replacement therapy. The risk of a person with diabetes being admitted to hospital for heart failure is 126% greater than among those without diabetes, while the risk of major amputation (above the ankle) is 400% higher.^12^ Furthermore, hospital length of stay is considerably greater for people with diabetes than those without,^13, 14^ further compounding the economic and healthcare system implications of the condition.

People diagnosed with diabetes are often active and working, faced with the challenge of attending numerous other medical appointments in cardiology, ophthalmology, renal assessment, endocrinology, and dietetics. Strain *et al*^15^ reported survey findings that physicians had low expectations of their diabetic patients in terms of attaining glycaemic control targets and following advice on diet and physical activity. Moreover, only a small proportion of diabetes patients believed lifestyle changes were important and the majority failed to comply.^15^ In terms of recall, people with diabetes had at best a rudimentary understanding of the risk of complications and the importance of good glycaemic control. Only 25% of patients surveyed in this multicountry evaluation reported being worried about developing type 2 diabetes complications.^15^

People with diabetes are encouraged to help manage their own disease through diet and exercise, blood pressure and glucose monitoring and insulin administration where indicated. The primary focus of clinical diabetes care revolves around a multidisciplinary team-based proactive approach to optimise risk factor control and thus minimise the risk of diabetes-related complications.

#### Early and sustained glycaemic control is important

Long-term prospective studies, some involving follow-up over several decades, underscore the benefit of early and sustained proactive glycaemic control in managing diabetes. These assessments conclusively show that reducing hyperglycaemia decreases the onset and progression of microvascular complications, such as retinopathy and nephropathy, in people with both type 1 and type 2 diabetes.^16, 17, 18, 19^

The Diabetes Control and Complications Trial (DCCT), a randomised clinical trial with mean follow-up of 6.5 years, showed that intensive glycaemic control effectively delays the onset and slows the progression of clinically important diabetic retinopathy (DR) (change of at least three steps from baseline that was maintained for at least six months), including vision-threatening retinopathy, nephropathy, and neuropathy in patients with insulin-dependent diabetes.^16^ Intensive treatment reduced the mean risk for development of retinopathy by 76% as compared with conventional therapy and slowed the progression of retinopathy by 54%.^16^

An observational follow-up study of the DCCT cohort demonstrated a significantly lower incidence of further DR progression in the intensive treatment group (hazard reduction 53–56%). The results indicate a durable beneficial effect, reinforcing the need for optimising glycaemic control as early as possible in patients with diabetes.^20^

The landmark UK Prospective Diabetes Study (UKPDS) was a large, multicentre, randomised controlled trial that compared the long-term effects of intensive blood glucose control (aim of fasting plasma glucose of <6 mmol/l) and conventional dietary treatment on the risk of microvascular and macrovascular complications in people with type 2 diabetes. Long-term follow-up results^17^ show that:

- Haemoglobin A1c was 53 mmol/mol (7.0%) in the intensive group compared with 63 mmol/mol (7.9%) in the conventional group over 10 years.
- Intensive blood glucose control substantially decreased the risk of diabetic microvascular complications in type 2 diabetes.
- Compared with the conventional treatment group, there was a 25% risk reduction (7–40, *P*=0.0099) in the intensive group in microvascular endpoints, including the need for retinal photocoagulation

Another study by the UKPDS Group, involving hypertensive patients with type 2 diabetes and a median follow-up of 8.4 years, showed that tight blood pressure control (144/82 mm Hg) achieved a clinically important reduction in the risk of deaths related to diabetes, complications related to diabetes, progression of DR, and deterioration in visual acuity.^21^ After 9 years of follow-up, the group assigned to tight blood pressure control had a 47% reduced risk (7–70%, *P*=0.004) of deterioration in visual acuity by 3 or more lines on the Early Treatment Diabetic Retinopathy Study (ETDRS) chart.^21^

Ten-year non-interventional post-trial monitoring was undertaken to determine whether improved glucose control persisted and whether such therapy had a long-term effect on macrovascular outcomes. Results revealed a continued reduction in microvascular risk and emergent risk reductions for myocardial infarction and death from any cause in the patient group originally assigned to receive intensive glucose-lowering therapy.^18^ Relative risk reductions persisted at 10 years for microvascular disease (24%, *P*=0.001). This was despite an early loss of between-group differences in HbA1c levels after the first year of post-trial monitoring.^18^

#### Multiple risk factor intervention for hyperglycaemia, hypertension, and dyslipidaemia

Sustained beneficial effects on vascular complications from intensive proactive multiple risk factor intervention was demonstrated in the Steno-2 Study, which involved 160 patients with type 2 diabetes and persistent microalbuminuria.^22^ With a mean follow-up of 7.8 years, results show that the relative risk of developing kidney, eye, and nerve complications all remained diminished by about 50% for the intensively-treated group, treated with angiotensin-converting-enzyme (ACE) inhibitors, statins, and glucose-lowering drugs. The impact of such an approach is highlighted by the fact that seven people in the conventional group became blind in one eye, compared with only one person in the group assigned to intensive therapy.^22^

Patients in the Steno-2 Study were subsequently followed observationally for a mean of 5.5 years. The intensive goal-directed multifactorial intervention group demonstrated sustained beneficial effects with regard to vascular complications as well as all-cause and cardiovascular complications.^23^

These data thus clearly illustrate the benefit of a proactive target driven multifactorial risk modification approach to optimising outcomes in people with type 2 diabetes.

#### Proactive clinical management of diabetes and co-morbidities, emphasis on patient-centred care

Given the evidence of benefit and supported by both national and international guidelines, a major focus for the care of people with type 2 diabetes is the achievement and maintenance of optimal glucose control early within the natural history of the condition.^17, 19, 24^ The clinical paradigm in managing glucose control centres on individualisation of therapy as there are many effective glucose-lowering agents to choose from when tailoring individual antihyperglycaemic treatment strategies for patients with type 2 diabetes. A stepwise approach is typically adopted, advancing from initial single drug therapy to dual combination and triple drug combination therapy when needed. Insulin therapy is considered a key component of any combination regimen when hyperglycaemia is severe.^19^

Treatment targets and strategies should be individualised based on a patient-centred approach (Table 3). This is reinforced in an updated joint position statement from the American Diabetes Association and the European Association for the Study of Diabetes.^19^ Personalisation of treatment is recommended, balancing the benefits and risks of glycaemic control, taking account of the potential risk of hypoglycaemia and other adverse effects and the patient's age and health status.^19^ Glycaemic control needs to be combined with a comprehensive cardiovascular risk factor reduction programme, to include smoking cessation and the adoption of other healthy lifestyle habits, blood pressure control, and lipid management.^19^ Personalised management plans for patients with type 2 diabetes should therefore encompass glycaemic control and effective blood pressure and lipid management

The initial management of type 2 diabetes involves advice and education about the potential benefits of dietary modification and lifestyle change, including increased physical activity. An early objective is to improve metabolic control through body weight reduction and thereby potentially improve insulin sensitivity.^25^ Many patients with diabetes however will require pharmacological therapy over time and often require escalation of treatment intensity. Only one in four patients in the UKPDS trial maintained an HbA1c level below 53 mmol/mol (7%) after 9 years without either oral agents or exogenous insulin.^25^

Over one-third of patients taking oral glucose-lowering medications report hypoglycaemic symptoms during the past year, which limited treatment satisfaction and therapy adherence.^26, 27^ Fear of hypoglycaemia drives poor therapy adherence and is one of the major barriers to the achievement of good glucose control from both a patient and healthcare professional perspective.^26^ In addition, severe hypoglycaemia has been identified as an independent risk factor in the aetiology of diabetes-related co-morbidities,^28^ while hypoglycaemia along with rapid improvements in glucose control contribute to an increase in retinal complications and blindness. Furthermore, severe hypoglycaemia and unawareness of hypoglycaemia can have serious socioeconomic implications, including suspension of a driving licence and reduced work productivity.^29, 30, 31^

A variety of risk factors are related to hypoglycaemia episodes, such as increasing age, longer duration of diabetes (years since diagnosis) and complexity of insulin regimen. Severe hypoglycaemia was found to be prevalent in almost half of people with long-duration type 1 diabetes.^32^ A retrospective cohort study by Bruderer *et al*^33^ found severe hypoglycaemia was recorded in 12 cases per 10 000 person-years in patients with type 2 diabetes in the United Kingdom who were newly treated with anti-diabetic drugs.

Other factors such as treatment complexity, weight gain, and socioeconomic factors can influence therapy adherence and hence glucose control.^26^ Indeed, poor treatment compliance, defined as medication non-compliance and/or non-attendance at medical appointments, was found to be associated with all-cause mortality in people with type 2 diabetes receiving insulin, in a study of patient data extracted from United Kingdom general practice records (*n*=15 984).^34^ Clinic non-attenders were more likely to be smokers, younger, have higher HbA1c and have more prior primary care contacts and greater morbidity.^34^

Patients decline participation in diabetes education programmes for a variety of reasons, commonly for logistical or financial reasons or because there is no perceived benefit. Non-attendance because of health-related shame and stigma of diabetes has been identified as a reason for non-attendance at structured education sessions in newly diagnosed type 2 diabetes patients.^35^ Alternative and innovative methods for delivering diabetes education beyond the conventional group format may encourage greater participation and individual empowerment.

#### Early intervention required to limit risk and progression of complications

Successful diabetes care requires a comprehensive proactive approach. A major objective of effective partnership between local primary care physicians and diabetology services is to maintain patients' quality of life and reduce treatment-related morbidity, such as hypoglycaemia. Patient engagement is central, encouraging individuals to know and own their data so as to maximise and maintain commitment and outcomes. Early and effective glycaemic control reduces microvascular risks and helps prevent later macrovascular complications.^36^ People with diabetes often have busy lifestyles and any initiative to reduce the amount of time required for hospital attendance or visits to the doctor's office is beneficial and may contribute to lowering overall healthcare costs.

*Summary:* What an ophthalmologist and other healthcare professionals need to know about diabetes.

- The primary focus of clinical diabetes care revolves around a multidisciplinary team-based proactive approach to optimise risk factor control and thus minimise the risk of diabetes-related complications.
- In people with diabetes, early optimal glycaemic control reduces the long-term risk of both microvascular and macrovascular complications.
- Fear of hypoglycaemia drives poor therapy adherence and is one of the major barriers to the achievement of good glucose control.
- Glycaemic control needs to be combined with a comprehensive cardiovascular risk factor reduction programme, to include smoking cessation and adoption of other healthy lifestyle habits, blood pressure control, and lipid management

*Summary:* National Institute for Health and Care Excellence (NICE) priorities for the management of type 2 diabetes in adults.^24^

- *Structured education*: Guidelines for managing adults with type 2 diabetes from NICE stress provision of structured education as an integral part of diabetes care.
- *Dietary advice and lifestyle modification*: Dietary advice should form part of a personalised diabetes management plan that includes other aspects of lifestyle modification, such as increasing physical activity and weight loss.
- *Management of blood glucose*: Adults should be actively encouraged to achieve their individual HbA1c target and maintain it unless adverse effects, or their efforts to achieve their target, impair their quality of life. If HbA1c levels are not adequately controlled by a single drug and rise to 58 mmol/mol (7.5%) or higher, clinicians are advised to reinforce dietary advice, lifestyle and adherence to drug treatment, support the person to aim for an HbA1c level of 53 mmol/mol (7.0%) and intensify drug treatment.
- *Hypertension*: Antihypertensive medications should be added if lifestyle advice does not reduce blood pressure to below 140/80 mm Hg (below 130/80 mm Hg if there is kidney, eye or cerebrovascular damage) and antihypertensive treatment intensified if necessary until the blood pressure is consistently below 140/80 mm Hg (below 130/80 mm Hg if there is kidney, eye or cerebrovascular damage).
- *Insulin therapy*: Insulin therapy for adults with type 2 diabetes should be considered if blood glucose levels are inadequately controlled despite dual therapy with metformin plus another oral anti-diabetic drug (if the person is markedly hyperglycaemic and prefers to start insulin rather than adding another drug), or where oral anti-diabetic drugs are contraindicated or not tolerated.

### Diabetic retinopathy screening in the United Kingdom: high coverage achieved, challenges and opportunities ahead

Screening for sight-threatening DR and maculopathy aims to detect diabetic eye disease at an appropriate stage in the disease process when treatment has a higher likelihood of success. Broad population coverage is essential and all people should be screened promptly and regularly after diagnosis of diabetes.

The rate of detection of referable DR is higher in those who are not screened promptly after diagnosis of type 2 diabetes, illustrating the need for strategies to increase uptake in those with a poor attendance record,^37^ for example, use of easy-to-read information and translated letters.^38^ Zoega *et al*^39^ described the relationship between non-attendance for diabetic eye disease screening and blind registration (VA <0.3) in a small sample of diabetes patients in Iceland, observing a significant relationship between screening compliance and visual outcome. Those people aged 18–34 years are least likely to attend promptly for screening after registration, with a higher risk of referable DR being present at first screen.^40^

#### Success in tackling vision impairment and blindness from DR in people of working age

**Figure 3:**
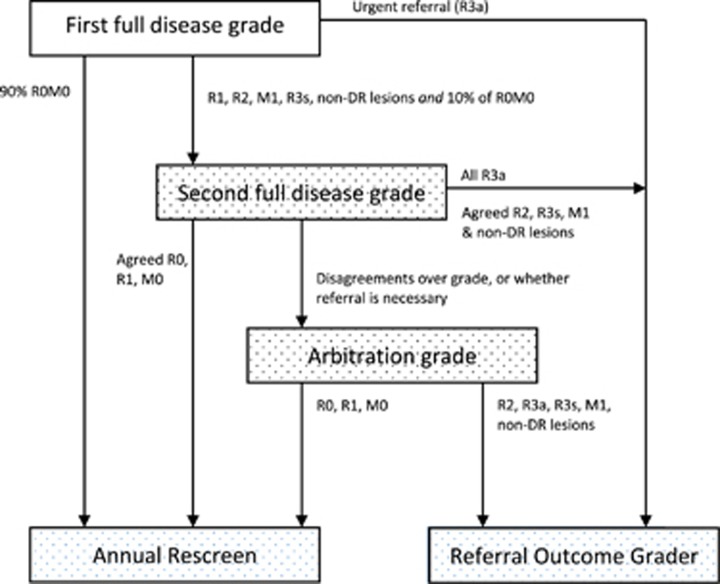
Retinal screening grading pathway, NHS England. Public Health England.^41^ Public health functions to be exercised by NHS England. Service specification No 22. NHS Diabetic Eye Screening Programme. Department of Health, NHS England, November 2013. Contains public sector information licensed under the Open Government Licence v3.0. Abbreviations: M0, no maculopathy; M1, maculopathy; R0, no retinopathy; R1, background retinopathy; R2, pre-proliferative retinopathy; R3a, active proliferative retinopathy; R3s, stable proliferative retinopathy.

The NHS Diabetic Eye Screening Programme involves a systematic approach designed to capture all people with diabetes for photographic screening once each year. People newly diagnosed with diabetes are invited annually for digital retinal photography screening and images are subjected to feature-based grading (Figure 3 outlines the retinal screening grading pathway).^41^ Assessments demonstrate the effectiveness of screening for DR by 2-field mydriatic digital photography, with good levels of sensitivity (87.8%) and specificity (86.1%) that compare well with seven field stereophotography and an ophthalmologist's examination using slit lamp biomicroscopy.^42, 43^ Those found to have potentially sight-threatening DR are referred to surveillance clinics or the NHS Hospital Eye Service.

The national screening programme has progressively evolved to achieve high population coverage in excess of 80% in United Kingdom. In 2014–2015, there were 83 local screening programmes involving both NHS and private providers in United Kingdom. That year, 2.5 million people with known diabetes were offered screening appointments and 2.1 million people were screened (84%). In the last 10 years, the epidemic of diabetes has seen an increase of 120 000 cases every 12 months. Of the 1.89 million people screened in United Kingdom in 2012–2013, 5.6% were graded background DR with maculopathy, 0.61% pre-proliferative DR without maculopathy, 0.59% pre-proliferative DR with maculopathy, 0.32% proliferative DR without maculopathy and 0.42% proliferative DR with maculopathy.

Similarly, the national Diabetic Retinopathy Screening Programme in Scotland screened 78.7% (201 299 individuals) of eligible people in 2014–2015, just below the Scottish national target of 80%.^44^ The national trend for 2014 was 8 916 new diabetes patients becoming eligible for screening across Scotland through 12 months. The annual percentage of referrals to ophthalmology on account of retinopathy is around 3.5%.^44^

Many people with diabetes have been prevented from losing vision through early detection and timely intervention. Liew *et al*^45^ reported that in persons aged between 16 and 64 years inclusive in 2009–2010, diabetic retinopathy/maculopathy was no longer the leading cause of certifiable blindness in United Kingdom and Wales, accounting for 14.4% of blindness certifications compared with 17.7% in 1999–2000. The underlying reasons for this change are thought to include the introduction of nationwide DR screening programmes in United Kingdom and Wales and improvements in glycaemic control.

Although DR is no longer the leading cause of severe sight impairment among working age adults in the United Kingdom, it remains a major cause of registrable blindness, second to hereditary retinal disorders. Prevalence figures from the National Ophthalmology Database show that, of a large cohort of diabetes patients assessed for DR in the UK Hospital Eye Service, almost 10% of eyes have centre-involving diabetic macular oedema (DMO) and around 20% have proliferative disease.^46^ Vitrectomy surgery for the late complications of proliferative DR continues to be required for some patients.^47^

### New challenges and opportunities to improve screening performance and referral refinement

#### Risk stratification and personalised screening intervals

Duration of diabetes is independently associated with microvascular events in type 2 diabetes, an effect that is greatest at younger age.^48^ The more prolonged the diabetes, the higher the prevalence of DR. However, the duration of diabetes is generally inconsistently documented and collated in clinical practice.

Better glycaemic control and to a lesser extent blood pressure control may be beneficial in reducing the incidence of proliferative DR and increasing the odds of improvement of DR.^49^ The 25-year cumulative rate of progression of DR was 83% and the rate of progression to proliferative DR was 42% in the Wisconsin Epidemiologic Study of Diabetic Retinopathy.^49^ Less severe DR, male gender, higher HbA1c, an increase in HbA1c level and an increase in diastolic blood pressure from the baseline to the 4-year follow-up increased the likelihood of progression of DR.^49^

The risk of progression of DR is significantly higher for those with background DR in both eyes than for those with background retinopathy in only one eye or in neither eye.^50^ The cumulative 4-year risk of development of referable DR was 1 in 3 for those with bilateral background DR, compared with less than 1 in 10 and 1 in 20 for those with only one eye or neither eye affected, respectively. With respect to the ETDRS severity scale, mild or non-proliferative DR or background DR (R1) identifies a minimum of at least the presence of one microaneurysm and/or retinal haemorrhage, equivalent to ETDRS levels 20–35; R1b defines the presence of these features in both eyes.^50, 51^

Two risk stratification models, one incorporating results from a single screening episode plus clinical information (HbA1c in the twelve months prior to screening and duration of diabetes) and another the results from two screening episodes alone, have been developed and validated.^52, 53^ These models discriminate well between those with very low and with high risk of progression to sight-threatening DR, which includes the risk of developing maculopathy as well as the risk of development of pre-proliferative DR or proliferative DR. Experience from the Diabetic Eye Screening Programme has helped develop risk stratification showing the risk for sight-threatening DR after 6 years is 1.7% compared with a risk for any retinopathy of 39%.^52^

The increasing numbers of people with diabetes presents challenges in maintaining a yearly screening service for all eligible diabetes patients. Following consultation, the UK National Screening Committee has recommended that in people at low risk of sight loss, the interval between screening tests should be changed from one year to two years. Extending the screening interval in selected low-risk cases is expected to reduce the number of unnecessary referrals to specialist eye services. Further outstanding questions need to be addressed however before implementation of these policy proposals. While observational studies in low-risk patients show little difference in clinical outcomes between 1 and 2-year screening intervals, Taylor-Phillips *et al*^54^ have argued there is insufficient evidence currently to support recommendations to extend the screening interval beyond one year.

#### Using optical coherence tomography as a second-line screening tool for those who are screen positive for maculopathy on 2-dimensional photographic markers: helping address over-referrals

The English Diabetic Eye Screening Programme has introduced surveillance into the approved service pathway standard. Cases that are classified as background DR with maculopathy (R1M1) in one or both eyes are also classified as having sight-threatening DR but are sometimes offered interim review appointments in surveillance clinics, depending on severity and circumstances.

Spectral domain optical coherence tomography (SD-OCT) imaging is a useful adjunct to colour fundus photography in screening for referable diabetic maculopathy.^55^ A prospective audit was performed of patients referred from the diabetic eye screening programme with mild to moderate non-proliferative DR (R1) and maculopathy in either eye attending an OCT-guided surveillance clinic. Results showed such cases had a 42.1% chance of having no DMO on SD-OCT imaging when graded by a retina specialist.^55^

In the Gloucestershire Diabetic Eye Screening Programme, the service pathway includes a surveillance clinic for certain cases that are classified on screening episode by digital photography as background DR and maculopathy in one or both eyes. Spectral domain OCT is utilised to grade and identify macular pathology. An audit of a consecutive case series (n=724) referred from the screening programme with background DR and maculopathy found only 20% needed to be referred to the NHS Hospital Eye Service for further review by an ophthalmologist, avoiding unnecessary hospital referral in 80% of screen-positive maculopathy cases (Table 4). Optical coherence tomography criteria and definitions used to grade scans as positive for referable diabetic maculopathy and borderline criteria are detailed in Table 5.

#### Automated image analysis software systems to augment manual graders

Manual grading is labour and capital intensive and requires trained, qualified graders. Systematic telemedicine digital retinal screening programmes have been introduced in a number of countries aiming to reach all people with diabetes.

Automated retinal image analysis systems may complement pure human grading, for example as an initial filter in routine screening prior to primary human grading or as a quality assurance tool, with manual grading as the reference standard.^56^ Studies are ongoing comparing the performance and economic costs of manual versus automated DR image assessment. Preliminary observational study results confirm that automated image assessment software systems provide acceptable sensitivity for referable retinopathy when compared with human graders, with sufficient specificity to make them cost-effective alternatives to manual grading alone.^56^

*Summary:* Retinal screening in the United Kingdom: progress and challenges.

- Although DR is no longer the leading cause of severe sight impairment among working age adults in the United Kingdom, it remains a major cause of registrable blindness.
- Strategies are needed to increase uptake in those with a poor attendance record.
- Risk stratification using one screening episode plus clinical risk factors (HbA1c in the twelve months prior to screening and duration of diabetes) or the results from two screening episodes alone can be used to identify low-risk groups who may require less frequent screening.
- Using OCT as a second-line screening tool in a screening surveillance clinic for those who are screen positive for maculopathy on photographic imaging may help reduce unnecessary referrals to the NHS Hospital Eye Service.
- Automated retinal image analysis systems may complement manual grading, providing an initial filter in routine screening prior to primary human grading or as a quality assurance tool.

### Patient experience and adherence with intravitreal injection therapy for visual impairment due to DMO: results of a qualitative research study

#### DMO is a common complication of DR

The risk for developing DMO is associated with duration of diabetes and severity of DR. Estimates of the DMO prevalence rate range from around 1 to 12% of all people with diabetes.^57^ A study estimated 7% of people with diabetes in United Kingdom had DMO, and of these, more than one-third (39%) had clinically significant macular oedema with visual impairment (visual acuity <6/6 in at least one eye).^58^

Therapeutic options for the management of DMO include laser photocoagulation, vitrectomy, corticosteroid therapy, and intravitreal anti-vascular endothelial growth factor (anti-VEGF) treatment. Macular laser photocoagulation has been the historic mainstay treatment for DMO over the past several decades. Intravitreal corticosteroid therapy has shown efficacy in improving VA in patients with DMO and may play a role in the treatment of adult pseudophakic DMO patients who fail to respond sufficiently to prior non-corticosteroid therapy, or are considered unsuitable for non-corticosteroid therapy.^59, 60, 61^ Anti-VEGF therapy, associated with substantially improved visual and anatomic outcomes compared with laser photocoagulation in patients with DMO,^62^ is considered the standard of care for initial management of eyes with visual impairment from central-involved DMO.^59, 62, 63^ Focal laser is an option for cases of non centre-involving DMO.^64^

Anecdotal evidence suggests that treatment adherence for some patients with intravitreal injection therapy for DMO is not optimal. A qualitative research study, commissioned by Bayer, was undertaken to elucidate patient experience and treatment adherence patterns amongst a diverse range of patients with DMO. The research programme also assessed patient compliance from the perspective of healthcare professionals and explored clinical and practical solutions that may help improve compliance, including clinic attendance. See Supplementary Information—Patient Experience and Adherence in DMO.

Individual in-depth interviews were conducted with 18 DMO patients being treated with licensed anti-VEGF therapy within the NHS Hospital Eye Service. Ten of 18 patients were regarded as non-compliant with treatment, defined as any missed appointment. The patient sample, drawn from ten different clinical centres in United Kingdom, Scotland, and Wales, was broadly mixed by age (range 35–65) and socioeconomic grouping. The majority (15/18) were men. Ten healthcare professionals (six consultant medical retina specialists, one advanced nurse practitioner and three nurse practitioners) from UK ophthalmology centres and all having experience in the management of both compliant and non-compliant DMO patients were also interviewed. Extracts are reproduced in Table 6.

#### Perspectives of the healthcare professional: proactive support may help improve health outcomes

Many patients do not immediately associate sight-threatening DMO with diabetes and time is required to explain the implications of their diabetic eye disease. People with diabetes need to be informed of the risks to their sight early on in their care pathway. Although it cannot be achieved in all patient groups, healthcare professionals should counsel patients on diabetes control, encourage them to understand the threat of vision loss from DMO and to manage their diabetes. Ethnic, cultural and language barriers however can make this approach difficult to achieve in all cases.

The thought of intravitreal injections and an intensive appointment schedule can be stressful for patients. The burden of treatment for bilateral DMO in particular can present challenges for patients who are coping with other diabetes-related co-morbidities. Anxiety often can persist throughout treatment and may be addressed with appropriate educational interventions.

Wilful or deliberate non-compliance was generally found to be rare. According to healthcare professionals interviewed, education about diabetes control could be better addressed, individually tailored to take account of ethnicity, age, and social needs. The introduction of a DSN in a dedicated pathway for patients with DMO may help to improve outcomes,^65^ generating positive patient experience and awareness and potentially improve individual motivation towards participation in structured education programmes and for attaining and maintaining good blood glucose and blood pressure targets.

#### Patients' perspectives

At the time of a diagnosis of DMO, patients typically experience worry, concern, and fear. For patients, fear of intravitreal injections and uncertainty over a proposed treatment plan without a determinate end often overshadow all other concerns. Explanations at the time of consultation about the importance of regular retreatment to avoid deterioration of vision and the association between diabetes, glycaemic control, and sight loss may be obscured by needle phobia or general treatment anxiety.

A lack of awareness of DMO, the risk of visual impairment if left unchecked, and the link to chronic diabetes was common. This dilutes the important communication that is required between doctor and patient in a DMO clinic. Patients require access to up-to-date educational materials about DMO, including useful signposting to authoritative sources, both before and at the point of diagnosis, to reinforce and encourage better diabetic control. Nine in ten adults report going online every day, reflecting the fact that internet use is an integral part of many people's daily lives.^66^ Multichannel access to educational materials increases awareness and involvement and may motivate the patient with regard to DMO treatment compliance and prioritisation of lifestyle modification to better control modifiable risk factors.

**Figure 4:**
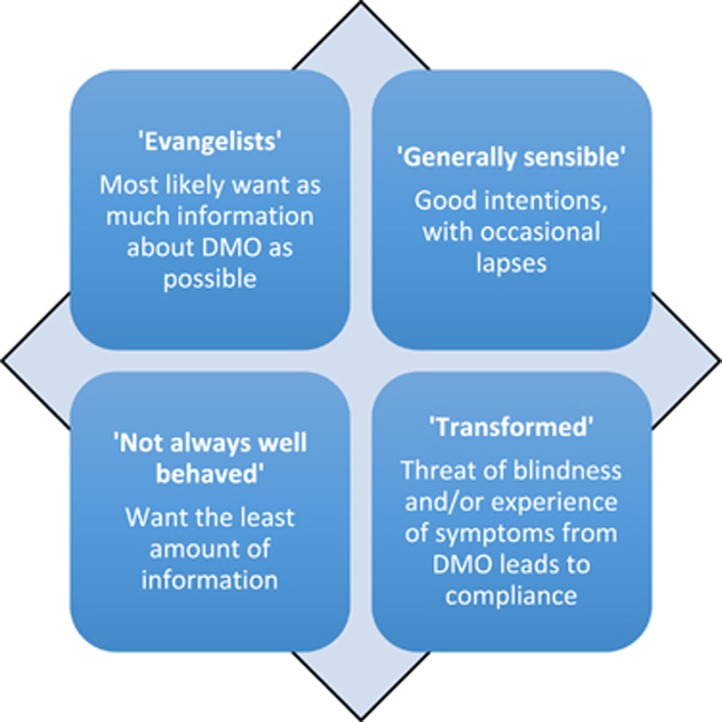
Characteristic psychological profiles of diabetes patients, covering attitudes to diabetes and its treatment. Three distinct types of diabetes patient were identified, which match DMO patient profiles, with the threat of blindness or experience of visual impairment creating an additional profile—‘Transformed'.

Three psychological profiles were identified in the research study that characterise the main differences amongst people with diabetic macular oedema attending DMO clinics: ‘evangelists', the ‘generally sensible' and ‘those not always well behaved' (Figure 4). The evangelical patient takes a keen interest in the condition, focused on adjusting lifestyle to help achieve better management of diabetes. They are disciplined and very compliant. The generally sensible profile probably covers the majority of patients, who listen, are interested, have good intentions and achieve good compliance most of the time, with occasional lapses in clinic attendance. Then there are patients who are not always well behaved, embracing a range of behaviours: past non-compliance, those in denial of their condition, or preoccupied with ‘chaotic' and busy lifestyles.

Attitudes towards DMO treatment adherence were found to mirror patients' views and behaviours in relation to the management and care of their own diabetes. Fear of visual impairment from DMO added a further patient profile: the ‘transformed'. These are patients who previously have not always been well behaved, but whose diabetic compliance has been markedly improved (transformed) by the diagnosis of DMO and resulting fear of blindness.

#### Experience of the DMO journey

Patients appreciate the explanation and reassurance provided by healthcare professionals at the time of commencing DMO treatment. However, they are aware of time and resource pressures within NHS hospital clinics. A perception of busy clinics can make patients reluctant to ask too many questions or take up too much time with clinicians. Not all patients want information upfront about DMO, preferring a stepwise approach one appointment at a time. Others prefer to absorb as much information as possible at the outset.

Compliant patients are more likely to understand the need for good diabetic control and are encouraged by threat or symptoms of sight loss. For such patients, explanation and counselling by healthcare professionals acts to support or reinforce compliance.

Not all patients with DMO receiving intravitreal anti-VEGF therapy are aware of the nature of the treatment regimen or understand the importance of the treatment intervals involved. Non-compliant patients are often least aware of the need to have repeat injections at prescribed intervals. This could be addressed by providing newly diagnosed DMO patients with good website links or patient information leaflets providing useful guidance about the course of treatment involved and about how treatment frequency typically peaks in the first year and diminishes over subsequent years.^67^

#### Mobile text messaging may encourage improved compliance with scheduled clinic visits

There were 91.5 million United Kingdom mobile subscriptions at the end of 2015.^66^ Seventy-one per cent of all UK adults own a smartphone, up from 66% in 2015.^66^ Ofcom research shows that 66% of adults claimed to access data services on a mobile phone in 2016, with a significant increase since 2015 noted for users aged 55 and older (20% of respondents aged 55+ in 2016 compared with 11% in 2015).^66^

Hence, text message reminders could be more widely utilised to reinforce and encourage good patient attendance for scheduled appointments and minimise ‘did not attend' rates, particularly for those who might be reluctant to turn up. A systematic literature review of studies on mobile telephone message reminders in healthcare services found 70% of the studies showed improved outcomes.^68^ Mobile phone text messaging reminders have been shown to increase attendance at healthcare appointments when compared to no reminders or postal reminders.^69^

Message content and timing in relation to an appointment should be carefully considered. The ideal may be a letter some weeks beforehand, so that time off work can be booked, followed by a simple text message reminder a couple of days prior to the appointment.

*Summary:* Qualitative research study exploring patient treatment adherence patterns with intravitreal injection therapy for DMO.

- People with diabetes need to be informed of the risks to their sight early on in the management of their diabetes care.
- Attitudes towards DMO treatment adherence appear to mirror patients' views and health behaviours in relation to the management and care of their own diabetes.
- Patients require access to up-to-date educational materials about DMO and the importance of adherence to treatment schedules, both before and at the point of diagnosis.
- Text message reminders could be more widely utilised to reinforce and encourage good patient attendance for scheduled appointments and minimise ‘did not attend' rates.

### Expanding the role of the diabetes specialist nurse in diabetic eye clinics

Practice interventions by a DSN promotes proactive modification of lifestyle behaviours and may improve glycaemic status and overall metabolic control. A randomised study in Sweden, involving data collection in 2010 and 2011, found nurse-led intervention focused on patient-centred self-management support lowered HbA1c among patients with type 2 diabetes at 12 months' follow-up.^70^ Glycated haemoglobin level significantly decreased from baseline in patient groups randomised to group intervention and individual intervention by 5 and 4 mmol/mol, respectively. An external control group recruited from another county council showed increased HbA1c from baseline.

**Figure 5:**
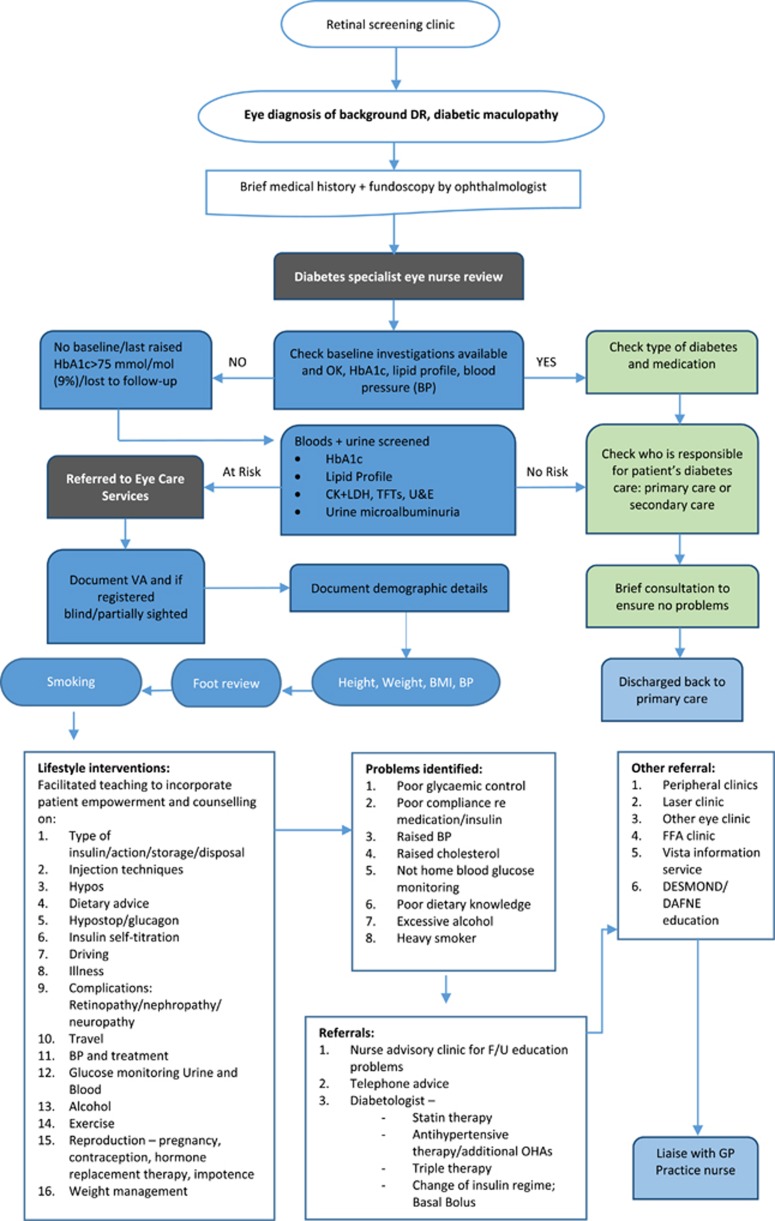
Type 1 and type 2 diabetes: diabetes specialist eye nurse care pathway, University Hospitals of Leicester. Abbreviations: BMI, body mass index; CK, creatine kinase; DR, diabetic retinopathy; FFA, fundus fluorescein angiography; F/U, follow-up; HbA1c, haemoglobin A1c; LDH, lactate dehydrogenase; OHAs, ocular hypertensive agents; TFTs, thyroid function tests; U&E, urea and electrolytes; VA, visual acuity. Reproduced with permission from UHL ophthalmology department.

An earlier published report from practitioners at University of Hospitals of Leicester NHS Trust showed positive results in an audit of a service pathway involving a diabetes specialist eye nurse working in both the diabetes and ophthalmology clinics (Figure 5 shows the care pathway and protocol).^65^ Khan *et al*^65^ reported improved glycaemic control and reductions in mean cholesterol levels at 12 months' follow-up in a cohort of 100 people with diabetic eye disease. For the full audited cohort, mean HbA1c level reduced from 71 mmol/mol (8.67%) at baseline to 60 mmol/mol (7.64%) at 12 months' follow-up examination.

A majority of diabetes patients with severe DMO (78–82% of 52 patients) failed to achieve a glycaemic control target of ≤48 mmol/mol (6.5%), in a retrospective audit of results from a regional hospital eye clinic in United Kingdom with conventional treatment without direct DSN intervention.^71^ The data suggest there is room for improvement in the management of diabetes patients being treated for DMO.

#### Exploring the role of the DSN in diabetic ophthalmic practice

The DSN role exists to educate and support people living with diabetes and their families at all stages of their lives, motivating people to self-manage their diabetes as effectively as possible. The DSN provides expertise as part of dedicated diabetes teams and assists other healthcare professionals in the care they provide. Considered the patient's advocate, the DSN can act as a valuable bridge between healthcare professionals and services in primary, secondary and integrated care. Nurse-led structured education programmes encourage people to manage modifiable risk factors for onset and progression of diabetic eye disease, by optimising control of blood glucose levels, blood pressure and lipid management.

Careful instructions are provided on when and how to self-test blood glucose and what to do with the results. Smoking cessation is stressed, as is the importance of regular eye examination for early identification and initiation of care for patients with retinopathy. The main modifiable risk factors for type 2 diabetes are overweight and obesity, insufficient physical activity and unhealthy dietary practices.^1^ Results of a parallel-group, randomised controlled trial at 56 primary care practices in Central and South United Kingdom found that an internet-based intervention with brief practice nurse support helped people maintain clinically important weight reductions over 12 months.^72^

Patients with diabetes require access to concise written material and online educational resources. Often there appears to be a lack of emphasis at the time of diabetes diagnosis on the relationship between managing known risk factors and development of diabetic eye disease. Diabetes patients should understand the need for good glycaemic control, as progression of eye disease is associated with the severity and duration of hyperglycaemia, and for effective treatment of hypertension.^73, 74, 75^

Stratton *et al*^76^ found that development of retinopathy in type 2 diabetes over 6 years from diagnosis was strongly associated with baseline glycaemia, glycaemic exposure over 6 years and higher blood pressure. Progression of existing retinopathy was associated with older age, male gender and hyperglycaemia. Intensive treatment of both hyperglycaemia and hypertension is advisable to help minimise the incidence of diabetes complications.^75^

Vision impairment negatively impacts quality of life and may restrict independence and mobility. Even mild impairment (near-normal vision) has a tangible influence on quality of life. Cumberland *et al*^77^ reported that, in a gradient of increasing severity, all-cause impaired visual function was associated with adverse social outcomes and impaired general and mental health. Vision-reliant tasks are required for good chronic disease management, including self-care (eg, foot checks in diabetes) and getting to and from clinic visits.^78^ Vision loss also may complicate the management of other conditions by creating difficulties in medication adherence and management, for example, administering insulin or eye drops.

The DSN can ensure baseline investigations for risk factors are completed at time of initial assessment for patients with DMO, refer patients at high risk of visual loss to a diabetologist (eg, poorly controlled hypertension, raised lipid parameters, microalbuminuria) and encourage good follow-up attendance for patients at risk of visual deterioration.

*Summary:* Expanding the role of the diabetes specialist nurse in dedicated eye clinics for patients with diabetic macular oedema.

- Often there appears to be a lack of emphasis at the time of diabetes diagnosis on the relationship between managing known risk factors and development of diabetic eye disease.
- Practice interventions by a diabetes specialist nurse (DSN) promotes proactive modification of lifestyle behaviours and may improve glycaemic status and overall metabolic control.
- Wherever feasible, the introduction of a DSN in a dedicated DMO eye clinic may help to improve care outcomes.
- The DSN can ensure baseline investigations for risk factors are completed at time of initial assessment following referral, direct patients at high risk of visual loss to a diabetologist and encourage good follow-up clinic attendance and treatment adherence.

### Practice principles and clinical considerations

In diabetes management, commitment of patients can deteriorate over time. People generally are more willing to consider behavioural change at the time of diabetes diagnosis. But enthusiasm typically wanes, with condition fatigue emerging with therapy escalation. Poor glucose control is often explained by clinical inertia, which limits or delays intensification of treatment when needed in the management of diabetes.^79^

Diabetic retinopathy and DMO are the two major retinal complications that account for most diabetes-related vision loss.^80^ Left unchecked, approximately half of people with DMO at baseline lose 2 or more lines of visual acuity within 2 years.^74^ Hyperglycaemia, hypertension and dyslipidaemia are risk factors for both the development and progression of DR/DMO and there is substantial evidence that control over metabolic factors can effectively prevent development and progression of potentially blinding diabetic eye disease.^74^

For optimal clinical care of patients with visual impairment due to DMO, several practice-based principles merit consideration in NHS ophthalmic service provision and care pathway redesign.

### Practice-based principles for optimal clinical care

#### Principle 1

Foster closer working relationships between diabetes management, general practitioners, and ophthalmology specialties. Closer clinical collaboration between diabetology, primary care, and ophthalmology services may enhance patient experience.

The ophthalmologist should establish who is taking care of the individual patient's diabetes and, as emphasised in clinical guidelines for DR from the Royal College of Ophthalmologists,^64^ develop strong links with local primary care and diabetology services to ensure that patients have effective integrated care plans for the management of their condition.^64^ The aim is to achieve closer working relationships so that patients with DMO or advanced or progressive retinopathy are appropriately managed from both the perspective of ophthalmology and diabetes management.

Much of the clinical focus in diabetes management is to limit the burden of diabetes-related complications. A structured approach to education concerning health behaviours and health promotion is beneficial and higher uptake should be encouraged. Multiple risk factor intervention is required to reduce disease burden and improve clinical outcomes, generating value at the individual, clinical and health system level.

In tailoring diabetes management to individual patients who are being treated for DMO, the diabetologist would benefit from knowing the patient's degree of sight loss, frequency of current treatment and expected prognosis. The diabetes management team should be aware of the main prognostic factors for increased risk of development and progression of sight-threatening DMO. This would enable the diabetes care team to identify high-risk patients at an early stage, for example, obesity, sleep apnoea, and elevated risk of progression of DR.^81^

#### Principle 2

Consider the benefits of establishing a dedicated clinic service for the management and follow-up of patients with DMO. Consider streamlining the pathway of diagnosis and treatment of DMO, with an emphasis on improving education and awareness, multidisciplinary working, expanding the role of the DSN in dedicated DMO clinics, and ensuring communication/liaison with colleagues beyond ophthalmology is effective and sufficient.

More time during consultation is needed for patients, to educate them about their diagnosis, treatment, outcomes, and diabetes control. Dedicated service provision for DMO would also facilitate integration of a DSN-led review service thus enhancing efficiency of diabetes care. The English Diabetic Retinopathy Screening Programme and guidance on retinal screening from the Royal College of Ophthalmologists have for a long time recommended dedicated assessment clinics in the NHS Hospital Eye Service for people with diabetic eye disease referred for ophthalmologist review.

Dedicated clinics for patients with diabetic eye disease would help promote:

- The value of early detection and intervention;
- Delivery of sustained education and health promotion;
- Multidisciplinary working and engagement with other healthcare professionals working with diabetes patients;
- Flexible service provision and clinics for greater patient engagement; and
- Identification of high-risk patients

Practice varies across the United Kingdom with regard to injection treatment clinics for DMO. While many centres choose to treat all eligible patients with retinal disease during a combined medical retina injection treatment service for example, other clinical centres prefer to maintain separate assessment and injection clinics for patients with DMO. Protocols for assessment and monitoring, as well as the recommended treatment posology with intravitreal anti-VEGF therapy, vary for different retinal disease entities.

#### Principle 3

Explore aspirations and opportunities to expand the role of the DSN in the hospital eye clinic, with the nurse specialist acting as the main hub between ophthalmology, endocrinology, diabetology, and primary care services/support. Specialist input from a diabetes expert is required when dealing with DMO patients coping with established diabetes-related complications and who may be at risk of mild or moderate vision impairment and of other later macrovascular complications because of poor metabolic control. Diabetes experts urge ophthalmologists to make better use of the specialist diabetes services available. Commissioners investing in local services should also support expansion and strengthen DSN recruitment so they may logistically be in a position to provide further coverage within hospital eye departments.

#### Principle 4

Tailor clinical practice and follow-up initiatives to improve treatment adherence in DMO. A wide range of appointment times should be available for patients with diabetes. Afternoon, evening or weekend appointments for working diabetic patients are often preferred, with experience suggesting only minor non-attendance rates for evening DMO clinics. Messaging beyond letter notification could be considered to reinforce the importance of a scheduled clinic appointment, for instance with follow-up text messaging. Text messaging can be quite effective for younger adults for example. For DR screening programmes, the regular non-attenders need to be vigorously chased.

#### Principle 5

Set realistic patient expectations when initiating treatment of DMO. Adequate support should be provided to ensure that patients understand the treatment they are being given and why that particular treatment is right for them, the treatment response they might expect, the most common side effects and the options that might be available if the initial treatment does not work well or does not suit them.

Where the treatment choice for DMO is intravitreal ant-VEGF therapy, patients should understand the goal of treatment, the need for regular repeat therapy, the treatment plan including follow-up regimen and be aware also that the frequency of injections may diminish after the first year of treatment.

Regular clinical attendance is required to resolve macular oedema and to ensure maintenance and/or improvement of vision long-term. Inadequate or delayed treatment may result in irreversible vision loss. The potential need for additional or substitute treatments should be discussed at the outset as there may be some cases of refractory macular oedema. Patients are reminded of the need to be proactive in optimising control of modifiable systemic risk factors. Several online sources of patient information and resources are outlined in Table 7.

#### Principle 6

Perform a regular audit of practice outcomes and benchmark performance, preferably using an electronic medical record (EMR) system. Audit and benchmark the key performance indicators of efficacy, safety, and treatment burden in the management and treatment of DMO. Development of a nationally agreed DMO outcome data set for EMR reporting will facilitate national audit data collection and shared learning.

### Best practice

Best practice models illustrate progress in strengthening service capacity and referral refinement of DR, including the use of OCT imaging as a second-line surveillance tool for evaluating referrals of screen-positive maculopathy. Illustrative examples include:

- *Refinement of screening referrals.* Consider establishing a second-line surveillance service for screen-positive maculopathy and/or pre-proliferative DR. OCT-guided surveillance clinics for screen-positive maculopathy and/or pre-proliferative DR provide additional flexibility in DMO management by helping minimise or limit false positive referrals to the Hospital Eye Service.^82^
- *Evening technician-led imaging clinics.* Evening technician-led OCT imaging clinics for DMO patients being treated with intravitreal anti-VEGF therapy have been introduced successfully within ophthalmology departments, uplifting capacity and allowing high-speed decision-making based on ophthalmologist review of acquired OCT scans. This frees up additional time for direct ophthalmologist review of more complex cases and for patient cases that may benefit from a treatment switch.
- *DSN-led reviews* of initial referrals of background DR or diabetic maculopathy after brief medical history and fundoscopy carried out by the eye clinic ophthalmologist, covering key baseline diabetes investigations and onward referral to the eye care service or discharge to primary care diabetes care.
- *Virtual review clinics utilising OCT imaging combined with fundus photography.* For patients with established DMO on regular intravitreal treatment and follow-up, a virtual OCT review clinic allows for separate assessment and grading of OCT images and fundus photographs by trained hospital technicians and nurses. Patients with stable disease are taken out of the existing DMO clinic service, releasing additional front-line treatment capacity.

## Conclusion

Findings from the Diabetic Retinopathy Clinical Research Network (DRCR.net) reiterate that long-term success in optimisation of glycaemic control as measured by HbA1c can be challenging.^83^ Investigators from DRCR.net identified a need for frequent educational interaction and additional communication with local primary diabetes care providers, as part of a comprehensive approach to the management of vision loss due to DR and DMO.^83^ People with diabetes need education to manage their condition and need to be informed of the risks to their sight early in the management of their diabetes care to ensure good compliance with regular eye checks.

Greater collaboration between eye health professionals and general practitioners, practice nurses and community-based diabetes care providers is recommended in order to ensure better coordinated follow-up and timely assessment of diabetes patients with related eye disease. Broader utilisation of and access to community-based diabetes care regimens can be expected to improve standards of patient care, and contribute to greater awareness of the need for improved glycaemic control to reduce diabetes-related complications and morbidity.

Evidence from randomised controlled trials supports treatment of proliferative DR and DMO to prevent progressive vision loss and imaging plays a valuable role in surveillance.^62^ Diabetes specialist nurses can help allay patient anxiety, reinforce knowledge of the value of adherence to treatment regimens and encourage appropriate health behaviours.

As an integral part of the patient pathway, ophthalmologists and other healthcare professionals play a vital role in disease management by encouraging and educating patients with DR and/or DMO to achieve important health targets, particularly for blood glucose and blood pressure control, to reduce the risk of progression of vascular complications and preserve visual function.

*Summary:* Action on DMO: best practice principles.

- Foster closer working relationships between diabetes management, general practitioners, and ophthalmology specialties.
- Consider the benefits of establishing a dedicated DMO eye clinic service for management and follow-up of patients with diabetic eye disease, for example, facilitate integration of a DSN-led review service and enhance efficiency of diabetes care.
- Explore aspirations and opportunities to expand the role of the DSN in the hospital eye clinic, with the specialist nurse acting as the main hub between ophthalmology, endocrinology, diabetology, and primary care services/support.
- Tailor clinical practice and follow-up initiatives to improve treatment adherence in DMO.
- Set realistic patient expectations when initiating treatment of DMO.
- Perform a regular audit of practice outcomes and benchmark performance, preferably using an EMR system.

## Figures and Tables

**Table 1:** Global prevalence estimates and healthcare expenditures 2015 and projection for 2040^a^

	*2015*	*2040*
*Global population*		
** **Total world population	7.3 billion	9.0 billion
** **Adult population (20–79 years)	4.72 billion	6.16 billion

*Diabetes (20–79 years)*		
** **Global prevalence	8.8% (7.2–11.4%)	10.4% (8.5–13.5%)
** **Number of people with diabetes	415 million (340–536 million)	642 million (521–829 million)
** **Number of deaths due to diabetes	5 million	

*Healthcare expenditure due to diabetes (20–79 years)*		
** **Total health expenditure, *R*=2^b^ 2015 US dollars	673 billion	802 billion

a Table adapted with permission from International Diabetes Federation.^2^

b *R*=2 estimate assumes healthcare expenditures for people with diabetes are on average twofold higher than for people without diabetes.

**Table 2:** Estimates of diabetes prevalence in the United Kingdom 2016^a^

*Country*	***N**umber of people diagnosed with diabetes in the adult population across the United Kingdom in 2016 (QOF diagnosed)*
United Kingdom	3 033 529
Northern Ireland	88 305
Scotland	280 023
Wales	188 644
Total	3 590 501

Abbreviation: QOF, Quality and Outcomes Framework 2015/16.

a Data included with permission from Diabetes UK.^4^

**Table 3:** Recommended approach to the management of hyperglycaemia in patients with type 2 diabetes: modulation of the intensiveness of glucose lowering based on patient and disease features, a broad construct to guide clinical decision-making^a^

*Patient/disease features*	*⇐ HbA1c 53* *mmol/mol (7.0%) ⇒*	
	*More stringent*	*Less stringent*
Risks potentially associated with hypoglycaemia and other adverse drug effects	Low	High
Disease duration	Newly diagnosed	Long-standing
Life expectancy	Long	Short
Important co-morbidities	Absent	Severe
Established vascular complications	Absent	Severe
Patient attitude and expected treatment efforts	Highly motivated, adherent, excellent self-care capacities	Less motivated, non-adherent, poor self-care capacities
Resources and support system	Readily available	Limited

Abbreviation: HbA1c, glycated haemoglobin.

a Adapted with permission from Inzucchi *et al.*^19^

**Table 4:** Referral decisions in a consecutive case series of diabetes patients following OCT-guided assessment in Gloucestershire Diabetic Eye Screening surveillance clinic (*n*=724)

*Referral outcome*	*Number of people*	*Percentage*
OCT/photographic clinic	426	59%
Referred to Ophthalmology Eye Clinic	146	20%
Annual screen	122	17%
Rebooked	21	3%
Deceased	9	1%

Abbreviation: OCT, optical coherence tomography.

**Table 5:** Criteria utilising spectral domain optical coherence tomography (OCT) as an adjunct to colour fundus photography in diabetic eye screening surveillance clinic

*OCT positive criteria:*
The presence of subretinal fluid or diffuse retinal thickening or intraretinal cystoid spaces associated with a change in the internal limiting membrane (ILM) or foveal contour
The presence of intraretinal cystoid spaces and associated with:
A drop in visual acuity (VA) to ≤6/12; or
With a large area of greater than 1 disc area of fluid the edge of which is within 1 disc diameter of the central fovea
Retinal thickness in the foveal central subfield ≥300 μm

*OCT borderline criteria:*
The presence of intraretinal cystoid spaces, or subretinal fluid, without any change in the ILM contour, and foveal central subfield ≤300 μm in the central 1 mm macular subfield
With VA ≥6/9
Without a large area of leakage of greater than 1 disc area the edge of which is within 1 disc diameter of the central fovea

**Table 6:** Revealing extracts, see Supplementary Information—Patient Experience and Adherence in DMO

*Perspectives, in the words of consultant retina specialists*
○ 'I always look to see what kind of diabetic patient they are. Their treatment history, how their HbA1c is controlled and what they do for themselves to manage their diabetes. It will tell me a lot about their diabetic macular oedema.'
— Consultant Retina Specialist
○ 'We really need more ability to intervene with diabetes patients to encourage behaviour change, but we just don't have the time or resources or links to other services. All I can really do is tell them what they should be doing and hope they listen.'
— Consultant Retina Specialist
*Perspectives, in the words of DMO patients*
○ 'The best thing I ever did was the Dose Adjustment For Normal Eating (DAFNE) course. I learnt about diet and exercise and how to manage my insulin and my diabetes.'
— Compliant DMO patient
○ 'I was really upset when I got the diagnosis (of diabetes), I didn't want to have a disease and certainly not one that would ruin my life.'
— Non-compliant DMO patient
*Experience of the DMO journey, in the words of DMO patients*
○ 'The reaction in my eyes was caused by a dramatic drop in my glucose levels. I was told it may have dropped too quickly and affected the vessels in my eyes.'
— Compliant patient
○ 'I was in bits when they told me I had DMO. It is bad enough being diabetic and now I could go blind! I felt like my life had ended and then they tell me I needed to have an injection in my eye and that was terrifying beyond belief.'
— Non-compliant DMO patient

**Table 7:** Online sources of patient information and resources

*Diabetes*
○ Diabetes UK. The leading charity that cares for and campaigns for people affected by or at risk of diabetes.
○ http://www.diabetes.org.uk/
○ Following a change in measurement reporting of HbA1c, Diabetes UK has developed an easy-to-use online HbA1c converter, for conversion of old (percentage units) and newer higher order measurements in millimoles per mol (mmol/mol).
○ http://www.diabetes.org.uk/HbA1c
○ Leicestershire Diabetes Service provides an extensive online diabetes resource.
○ http://www.leicestershirediabetes.org.uk/
*Diabetic eye disease*
○ Moorfields Eye Hospital NHS Foundation Trust. Facts about diabetic macular oedema. Patient information - medical retina services. Authored by Ms Dawn Sam, revision number 4, May 2016.
○ http://www.moorfields.nhs.uk/sites/default/files/diabetic-macular-oedema.pdf
○ The Macular Disease Society. Your guide to diabetic macular oedema. Macular Society 2015.
○ http://www.macularsociety.org/sites/default/files/resource/Diabetic Macular Oedema.pdf
○ Royal National Institute for the Blind (RNIB). Understanding eye conditions related to diabetes. RNIB and RCOphth July 2016. Accredited by the Royal College of Ophthalmologists, this guide provides a detailed explanation of the eye and helpful advice on next steps.
○ http://www.rnib.org.uk/sites/default/files/Understanding_eye_conditions_related_to_Diabetes.pd_related_to_Diabetes.pdf
○ NHS Public Health England. Your guide to diabetic retinopathy. Important information about signs of changes to your eyes caused by diabetes. Public Health England.
○ http://www.gov.uk/government/uploads/system/uploads/attachment_data/file/499568/DES_03_Your_guide_to_diabetic_retinopathy_single_pages_090216.pdf
○ NHS Public Health England. Closer monitoring and treatment for diabetic retinopathy. Public Health England.
○ http://www.gov.uk/government/uploads/system/uploads/attachment_data/file/502429/DES_05_web_version_230216.pdf
○ Further online information about diabetic eye screening and other aspects of diabetes care is available at http://www.nhs.uk/diabeticeye
*Driving*
○ The Macular Disease Society. Driving. Everything you need to know about driving if you have macular disease. Also available on audio CD. Macular Society 2016.
○ http://www.macularsociety.org/sites/default/files/downloads/Macular Society Driving 0316.pdf
