# Supplementary material for: Action on diabetic macular oedema: achieving optimal patient management in treating visual impairment due to diabetic eye disease
Source: Eye (Lond). 2017 May 11;31(Suppl 1):S1–S20. doi: 10.1038/eye.2017.53 (PMC5437340; doi:10.1038/eye.2017.53)
Supplement: Supplementary Information [file eye201753x1.ppt]

## Slide 1
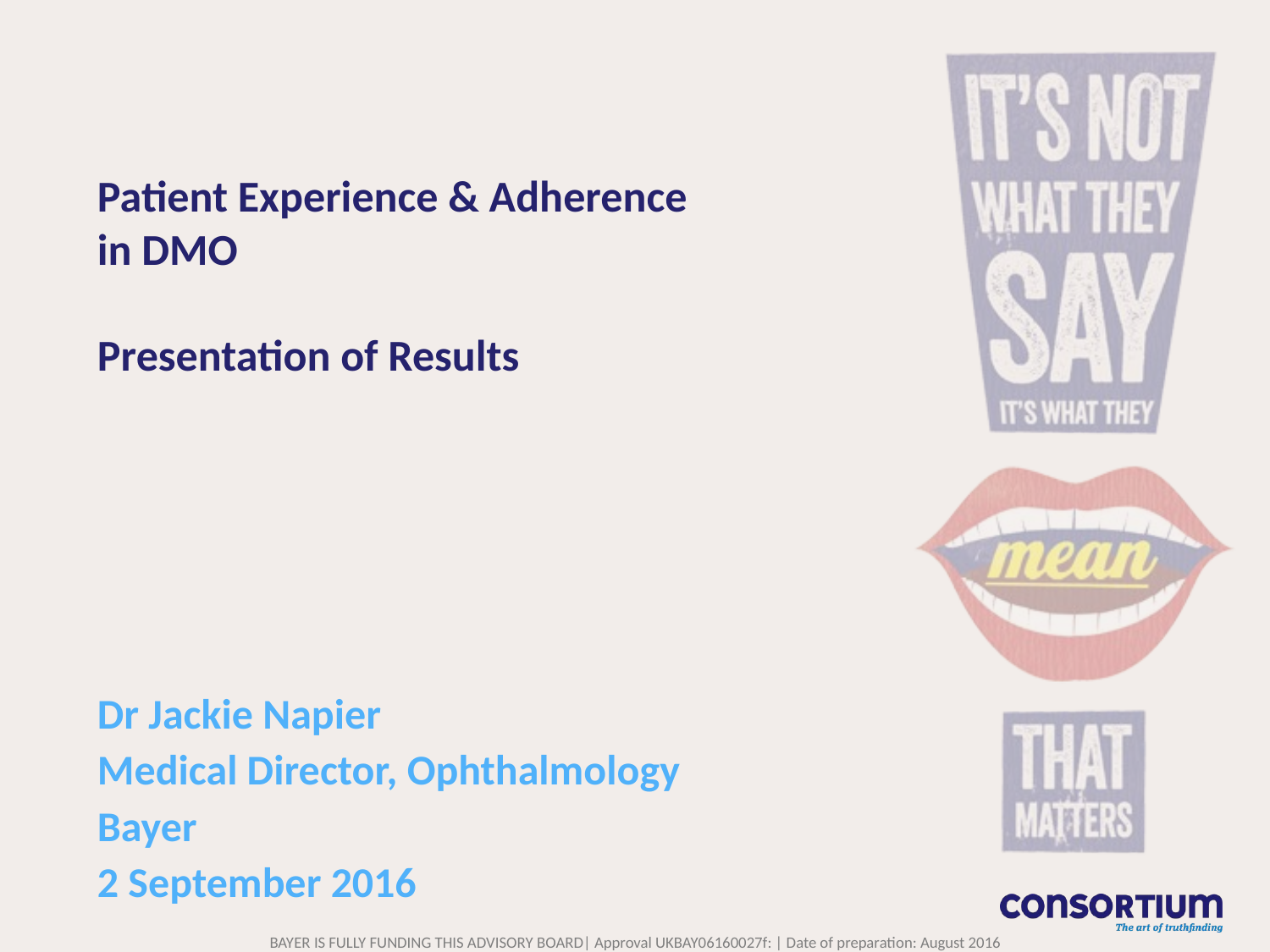

# Patient Experience & Adherence in DMO Presentation of Results
Dr Jackie Napier
Medical Director, Ophthalmology
Bayer
2 September 2016
BAYER IS FULLY FUNDING THIS ADVISORY BOARD| Approval UKBAY06160027f: | Date of preparation: August 2016

## Slide 2
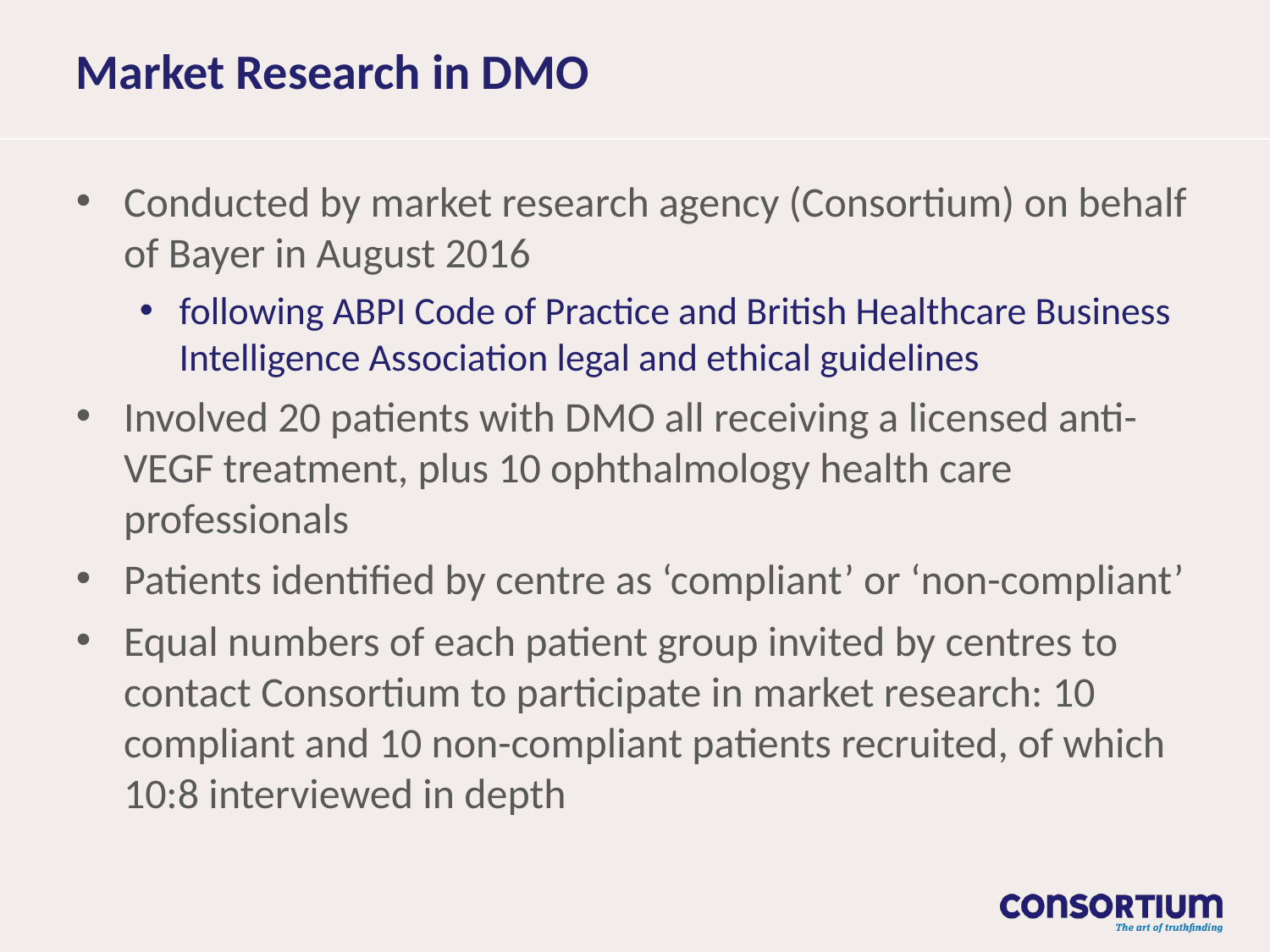

# Market Research in DMO
Conducted by market research agency (Consortium) on behalf of Bayer in August 2016
following ABPI Code of Practice and British Healthcare Business Intelligence Association legal and ethical guidelines
Involved 20 patients with DMO all receiving a licensed anti-VEGF treatment, plus 10 ophthalmology health care professionals
Patients identified by centre as ‘compliant’ or ‘non-compliant’
Equal numbers of each patient group invited by centres to contact Consortium to participate in market research: 10 compliant and 10 non-compliant patients recruited, of which 10:8 interviewed in depth

## Slide 3
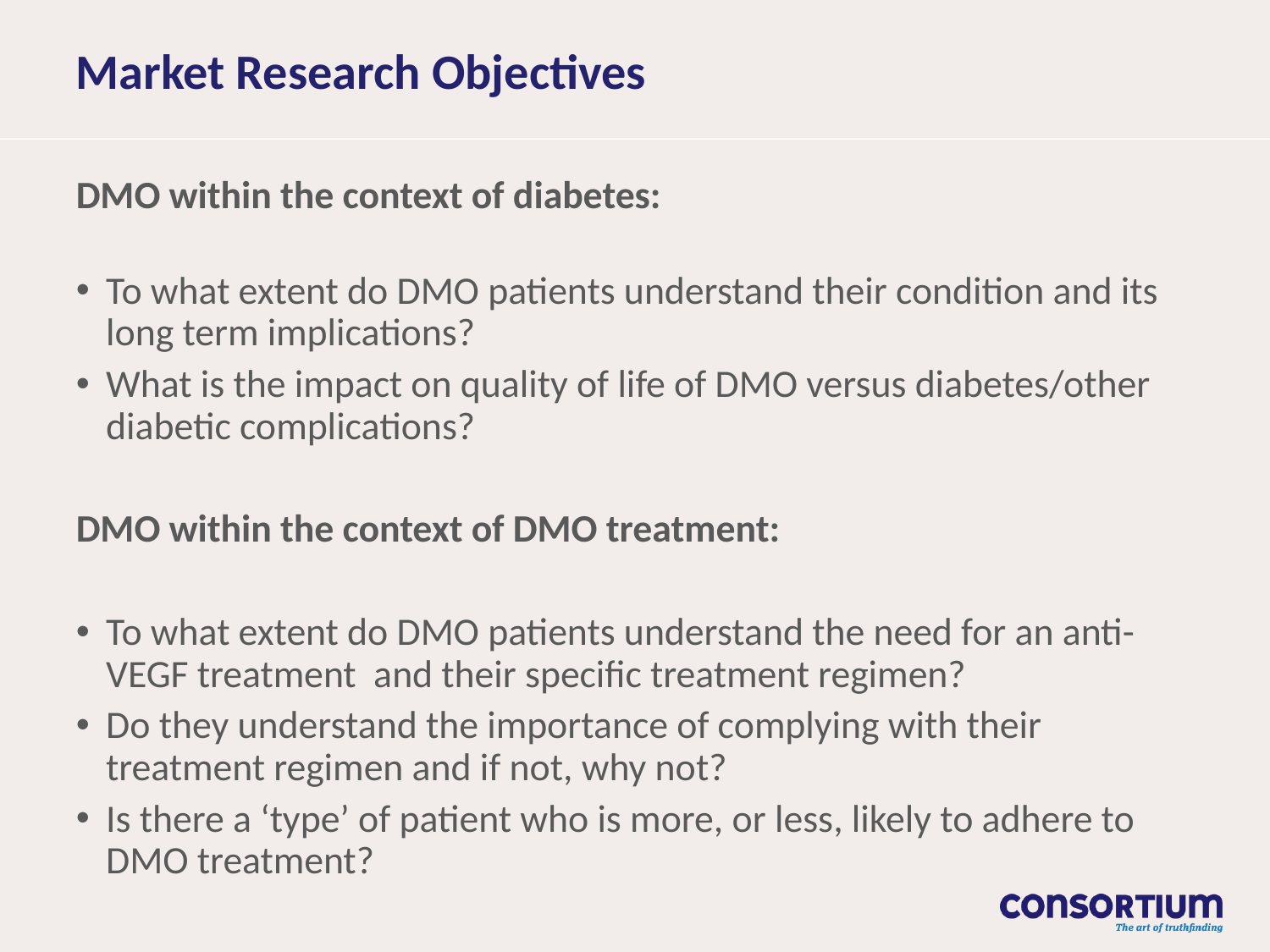

# Market Research Objectives
DMO within the context of diabetes:
To what extent do DMO patients understand their condition and its long term implications?
What is the impact on quality of life of DMO versus diabetes/other diabetic complications?
DMO within the context of DMO treatment:
To what extent do DMO patients understand the need for an anti-VEGF treatment and their specific treatment regimen?
Do they understand the importance of complying with their treatment regimen and if not, why not?
Is there a ‘type’ of patient who is more, or less, likely to adhere to DMO treatment?

## Slide 4
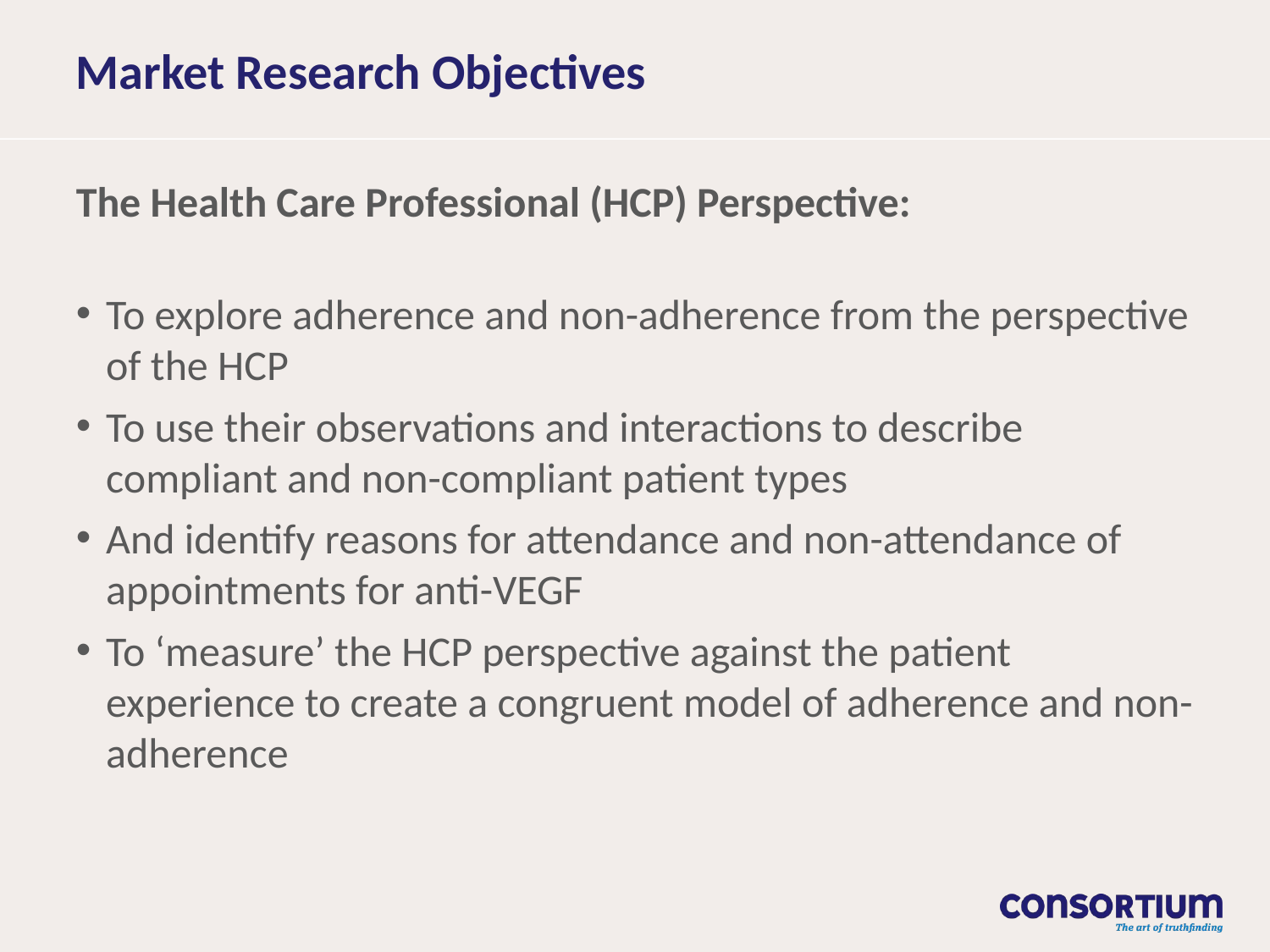

# Market Research Objectives
The Health Care Professional (HCP) Perspective:
To explore adherence and non-adherence from the perspective of the HCP
To use their observations and interactions to describe compliant and non-compliant patient types
And identify reasons for attendance and non-attendance of appointments for anti-VEGF
To ‘measure’ the HCP perspective against the patient experience to create a congruent model of adherence and non-adherence

## Slide 5
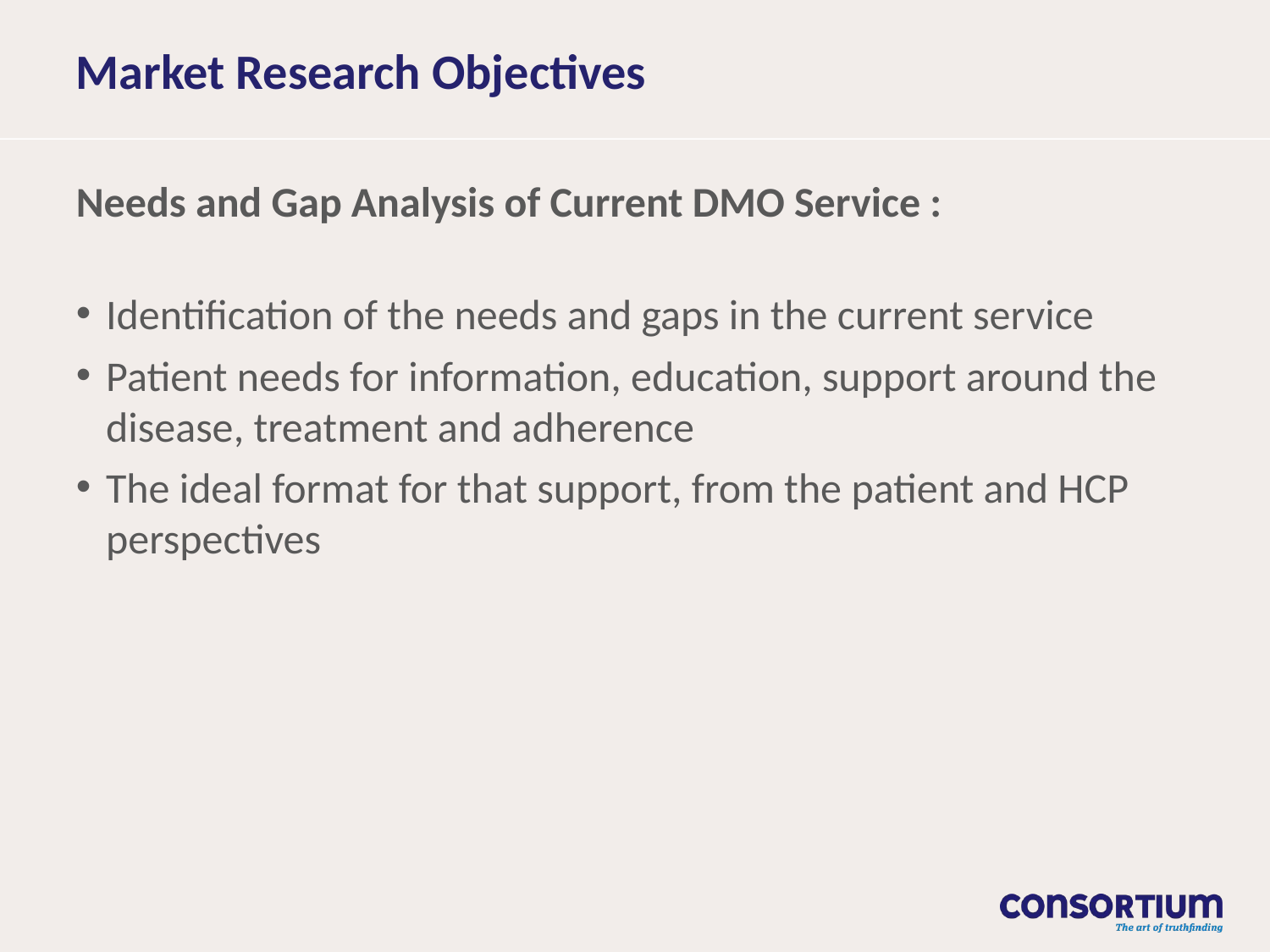

# Market Research Objectives
Needs and Gap Analysis of Current DMO Service :
Identification of the needs and gaps in the current service
Patient needs for information, education, support around the disease, treatment and adherence
The ideal format for that support, from the patient and HCP perspectives

## Slide 6
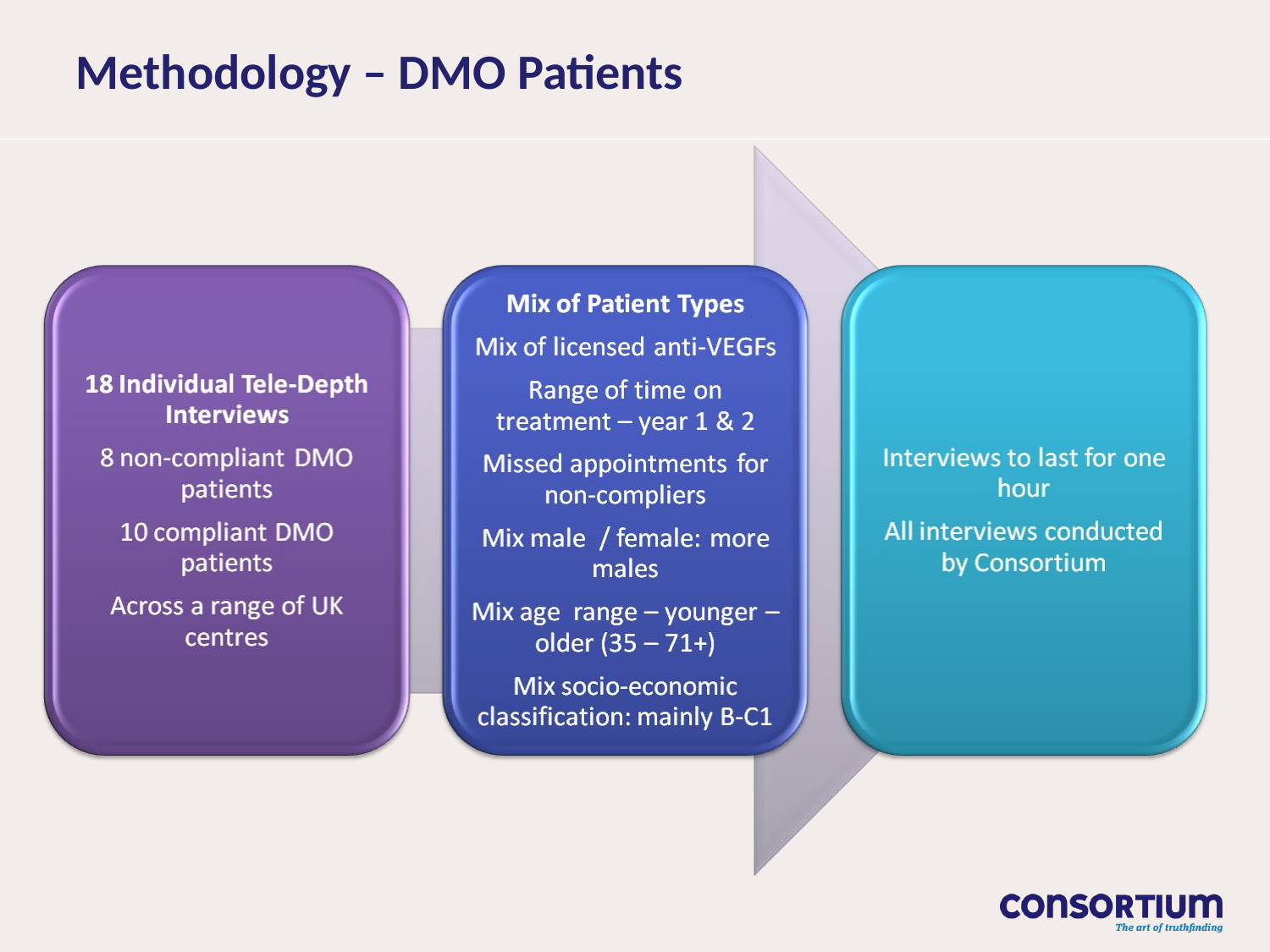

# Methodology – DMO Patients

## Slide 7
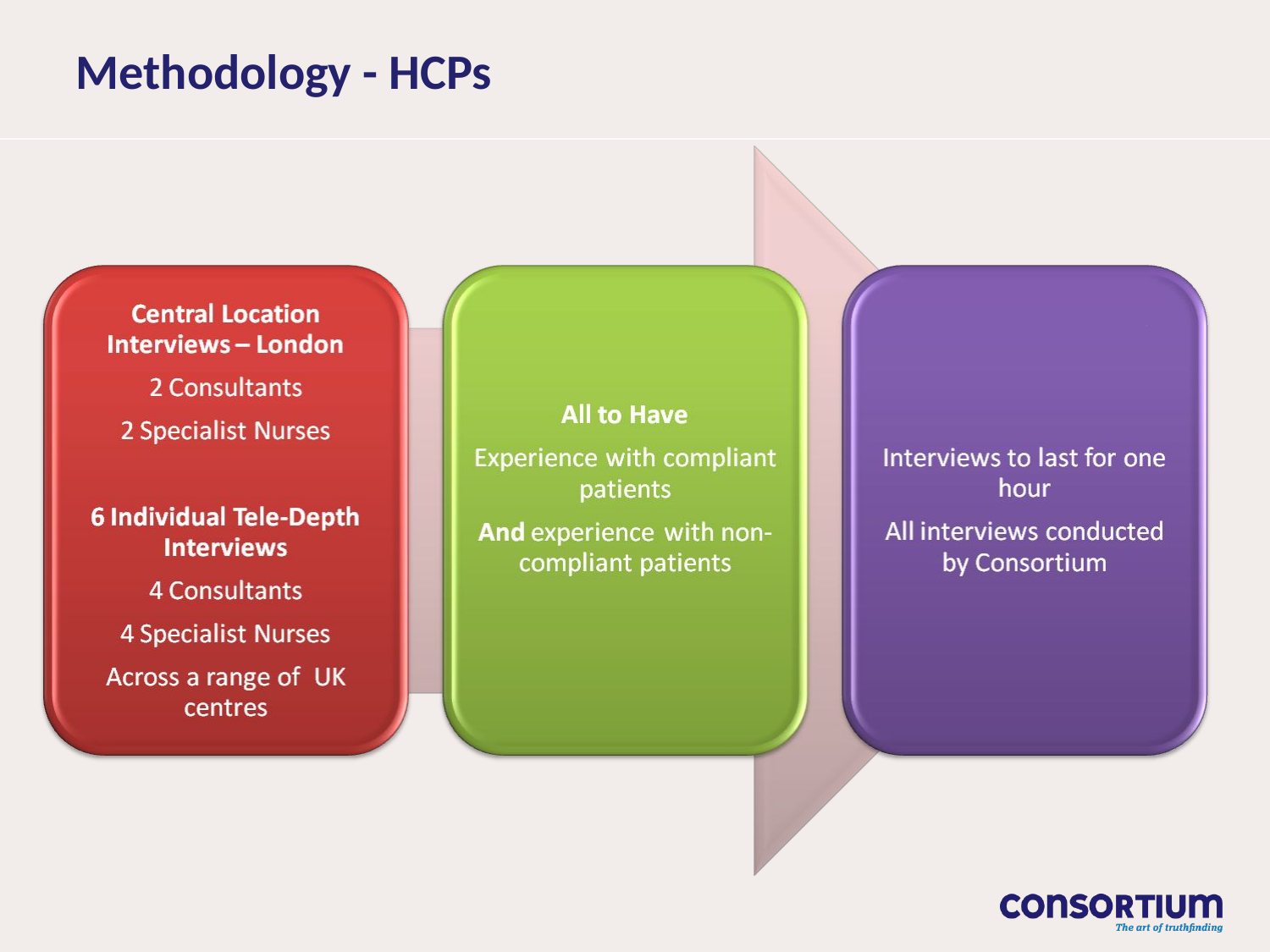

# Methodology - HCPs

## Slide 8
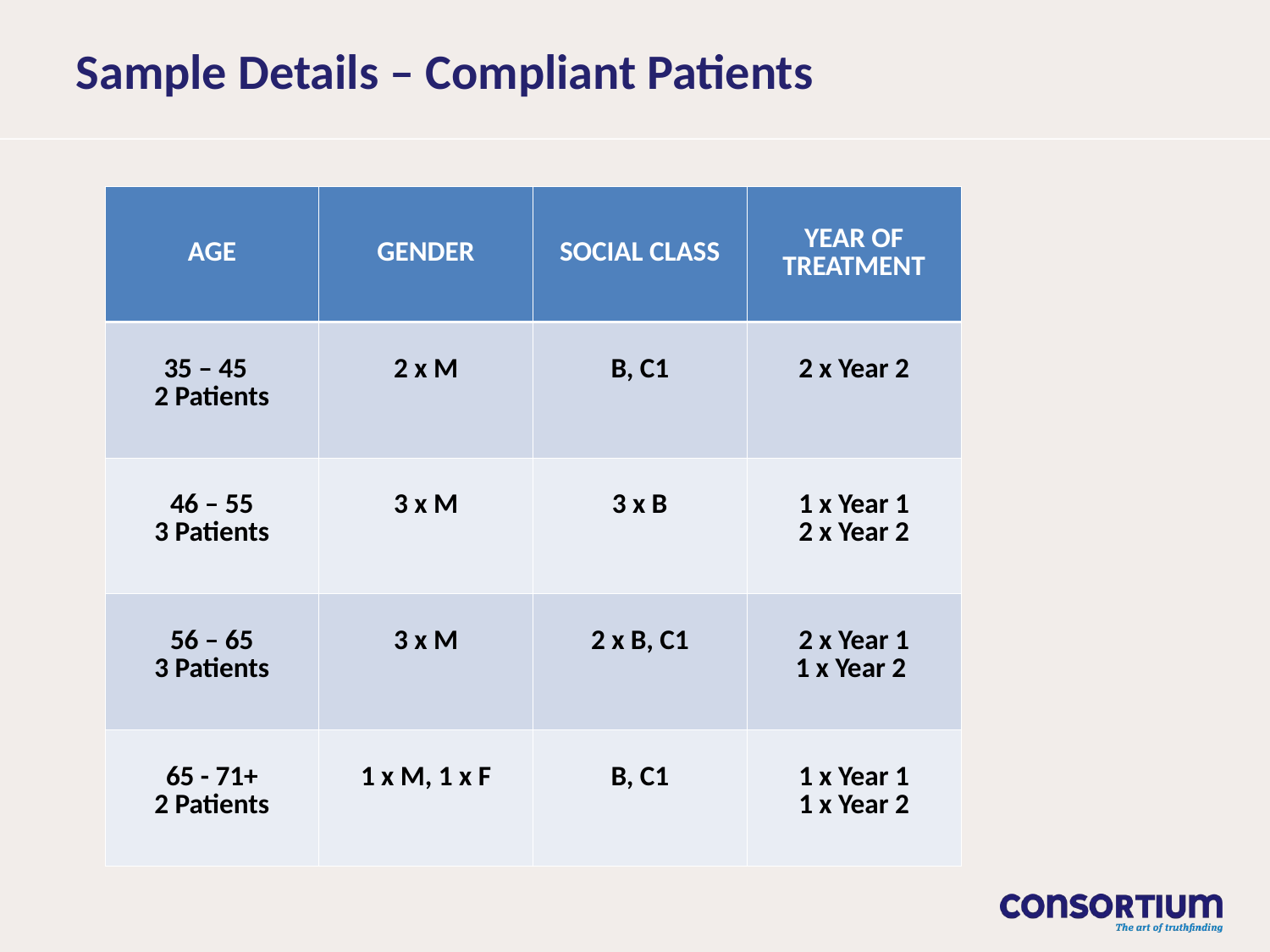

# Sample Details – Compliant Patients
| AGE | GENDER | SOCIAL CLASS | YEAR OF TREATMENT |
| --- | --- | --- | --- |
| 35 – 45 2 Patients | 2 x M | B, C1 | 2 x Year 2 |
| 46 – 55 3 Patients | 3 x M | 3 x B | 1 x Year 1 2 x Year 2 |
| 56 – 65 3 Patients | 3 x M | 2 x B, C1 | 2 x Year 1 1 x Year 2 |
| 65 - 71+ 2 Patients | 1 x M, 1 x F | B, C1 | 1 x Year 1 1 x Year 2 |

## Slide 9
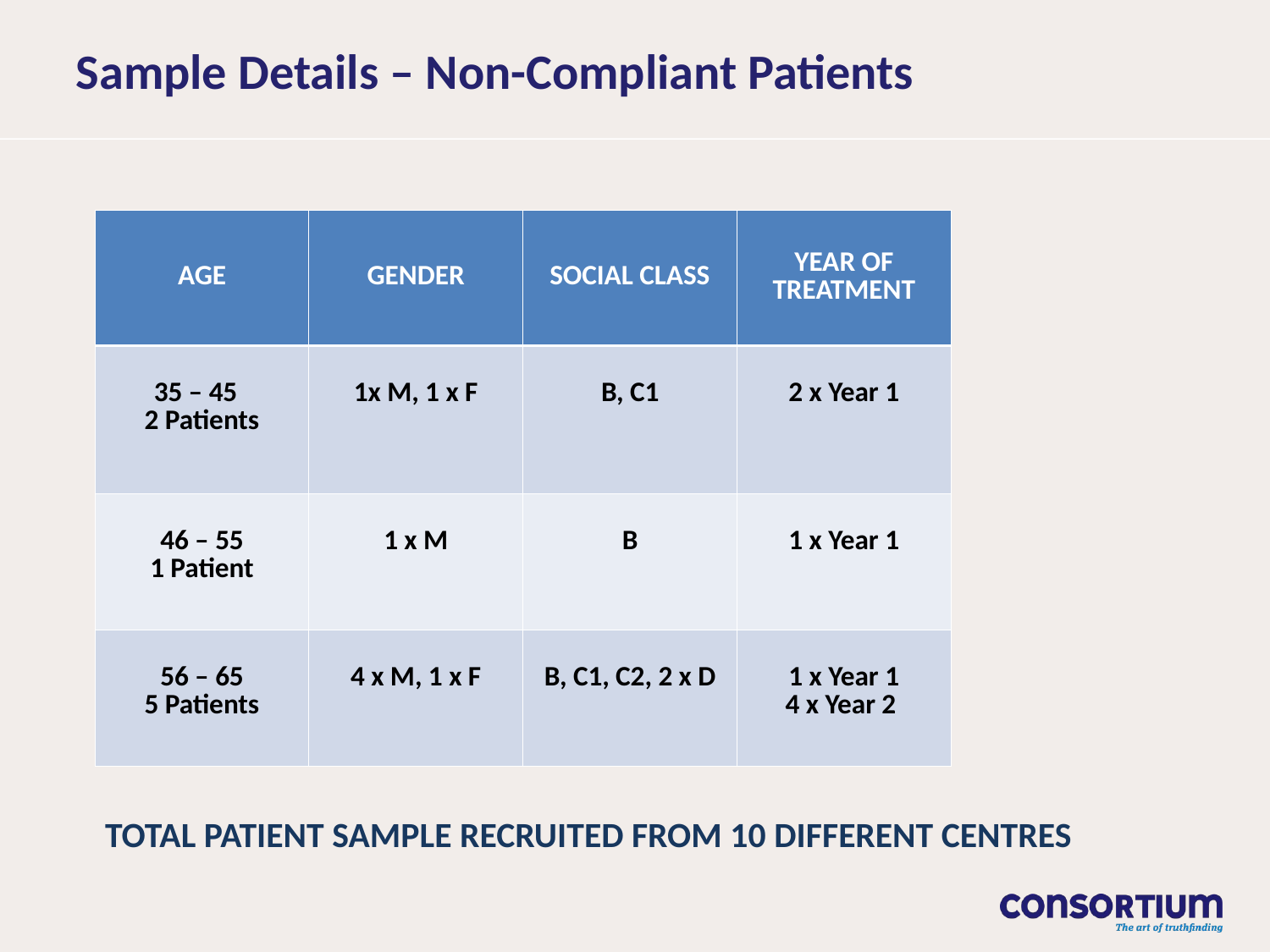

# Sample Details – Non-Compliant Patients
| AGE | GENDER | SOCIAL CLASS | YEAR OF TREATMENT |
| --- | --- | --- | --- |
| 35 – 45 2 Patients | 1x M, 1 x F | B, C1 | 2 x Year 1 |
| 46 – 55 1 Patient | 1 x M | B | 1 x Year 1 |
| 56 – 65 5 Patients | 4 x M, 1 x F | B, C1, C2, 2 x D | 1 x Year 1 4 x Year 2 |
 TOTAL PATIENT SAMPLE RECRUITED FROM 10 DIFFERENT CENTRES

## Slide 10
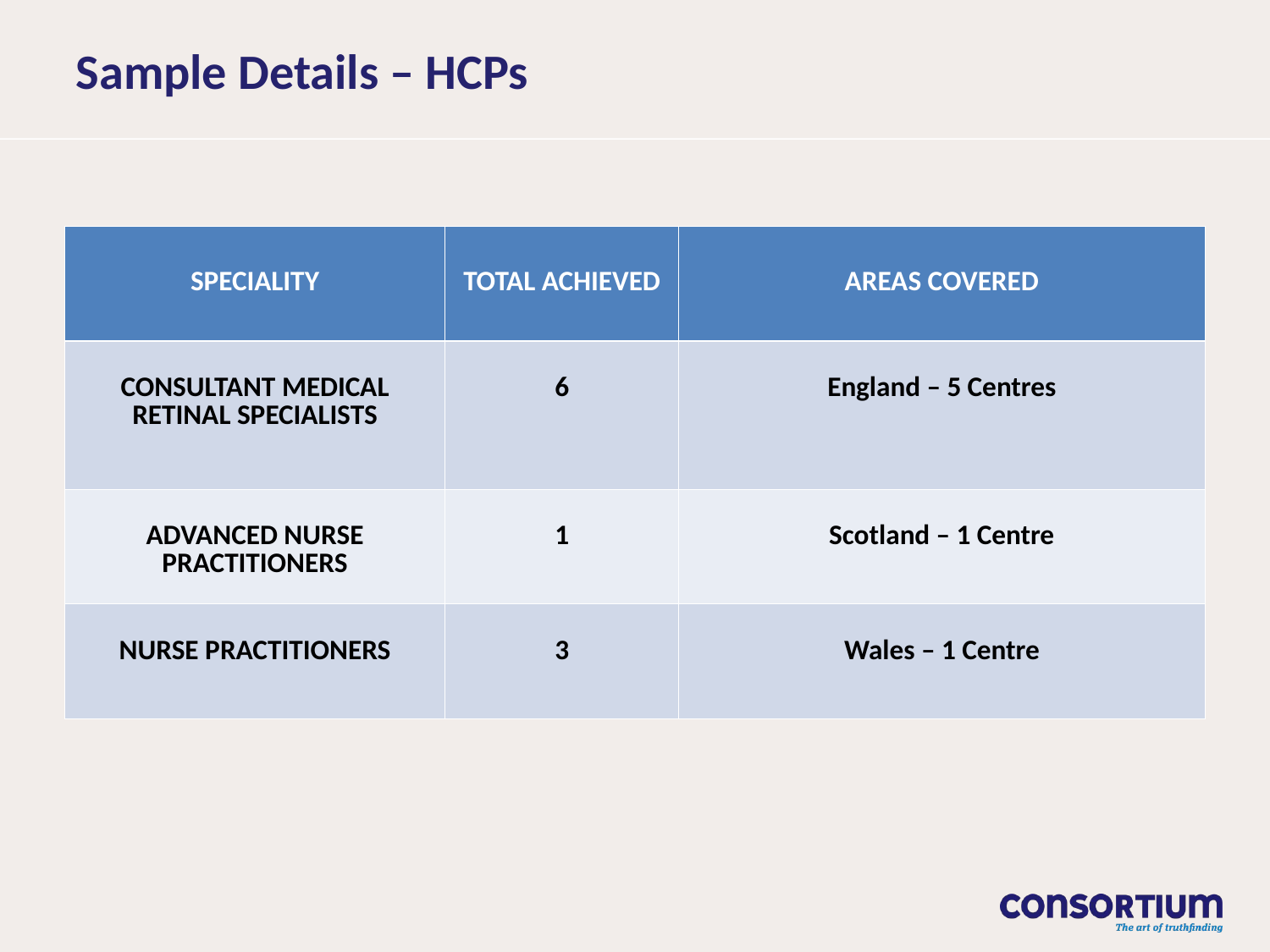

# Sample Details – HCPs
| SPECIALITY | TOTAL ACHIEVED | AREAS COVERED |
| --- | --- | --- |
| CONSULTANT MEDICAL RETINAL SPECIALISTS | 6 | England – 5 Centres |
| ADVANCED NURSE PRACTITIONERS | 1 | Scotland – 1 Centre |
| NURSE PRACTITIONERS | 3 | Wales – 1 Centre |

## Slide 11
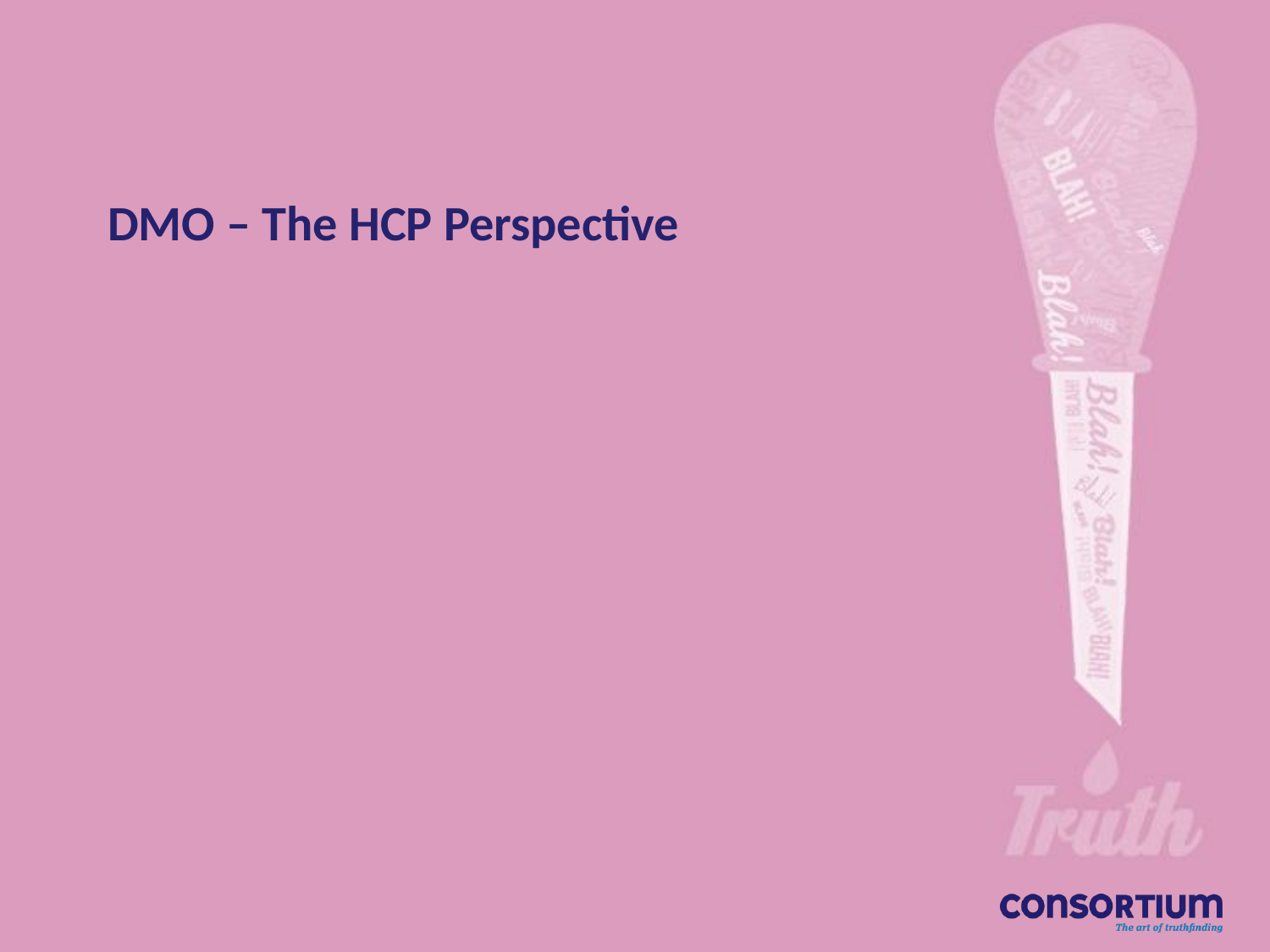

# DMO – The HCP Perspective

## Slide 12
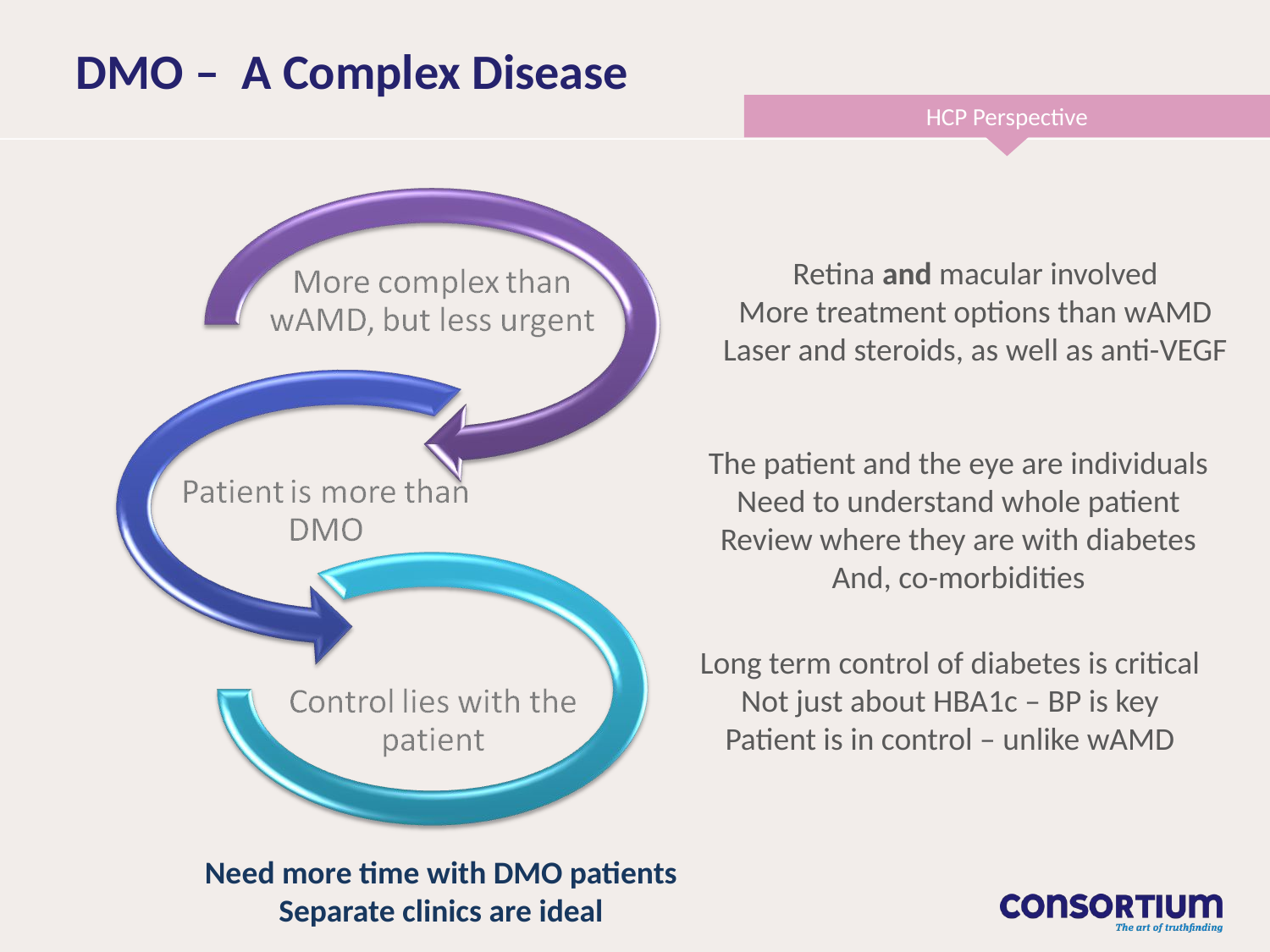

# DMO – A Complex Disease
HCP Perspective
Retina and macular involved
More treatment options than wAMD
Laser and steroids, as well as anti-VEGF
The patient and the eye are individuals
Need to understand whole patient
Review where they are with diabetes
And, co-morbidities
Long term control of diabetes is critical
Not just about HBA1c – BP is key
Patient is in control – unlike wAMD
Need more time with DMO patients
Separate clinics are ideal

## Slide 13
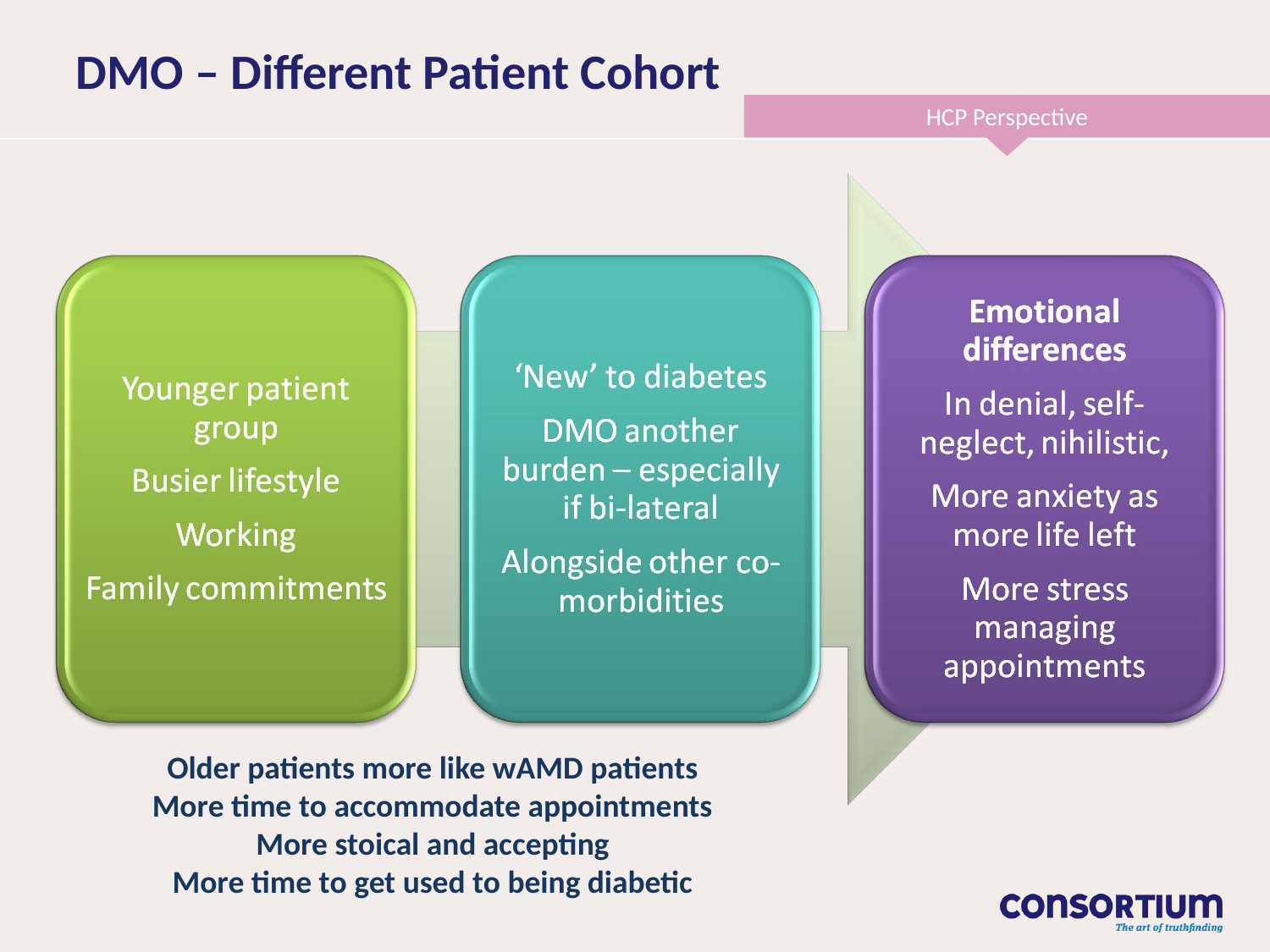

# DMO – Different Patient Cohort
HCP Perspective
Older patients more like wAMD patients
More time to accommodate appointments
More stoical and accepting
More time to get used to being diabetic

## Slide 14
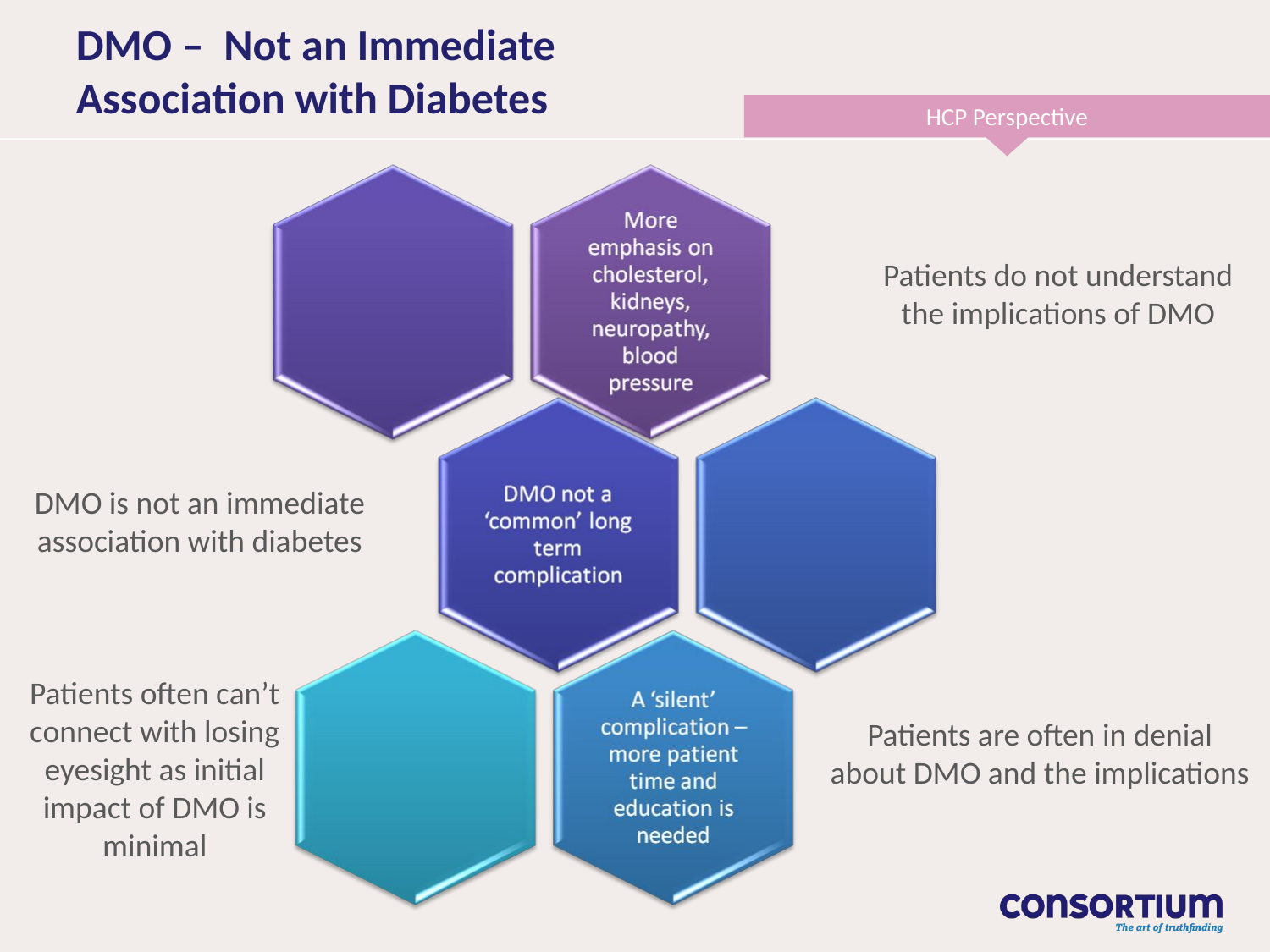

# DMO – Not an Immediate Association with Diabetes
HCP Perspective
Patients do not understand the implications of DMO
DMO is not an immediate association with diabetes
Patients often can’t connect with losing eyesight as initial impact of DMO is minimal
Patients are often in denial about DMO and the implications

## Slide 15
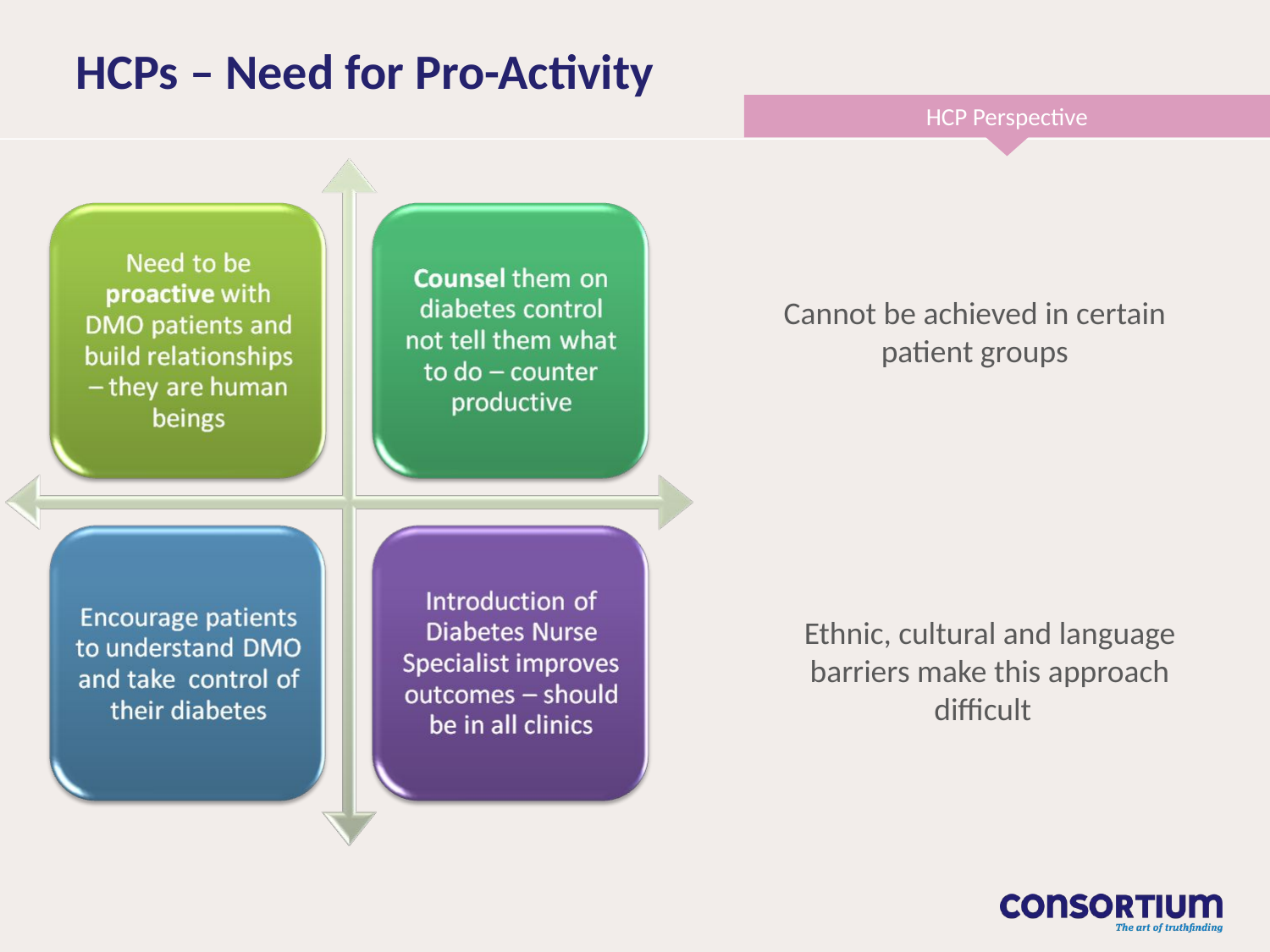

# HCPs – Need for Pro-Activity
HCP Perspective
Cannot be achieved in certain patient groups
Ethnic, cultural and language barriers make this approach difficult

## Slide 16
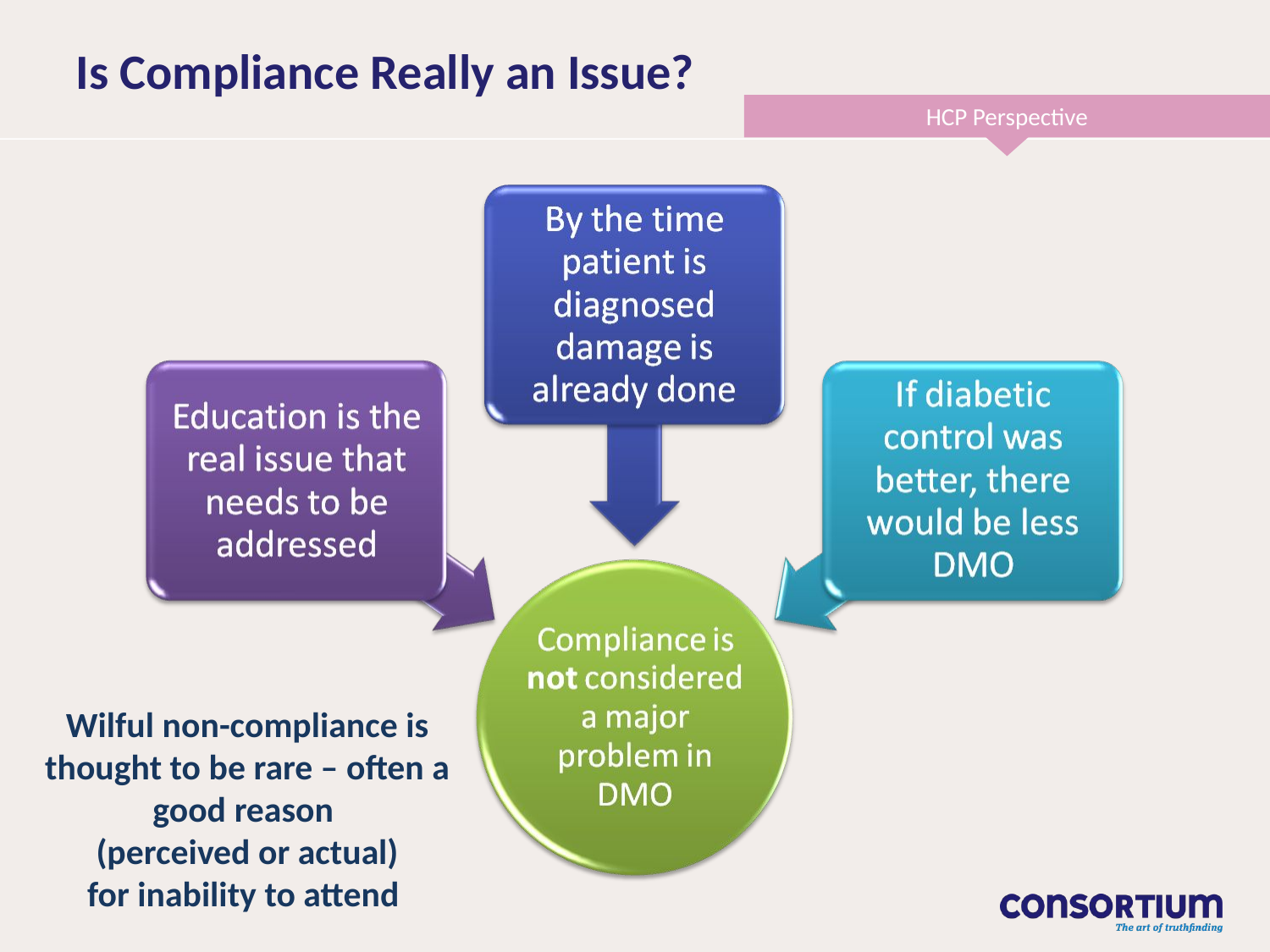

# Is Compliance Really an Issue?
HCP Perspective
Wilful non-compliance is thought to be rare – often a good reason
(perceived or actual)
for inability to attend

## Slide 17
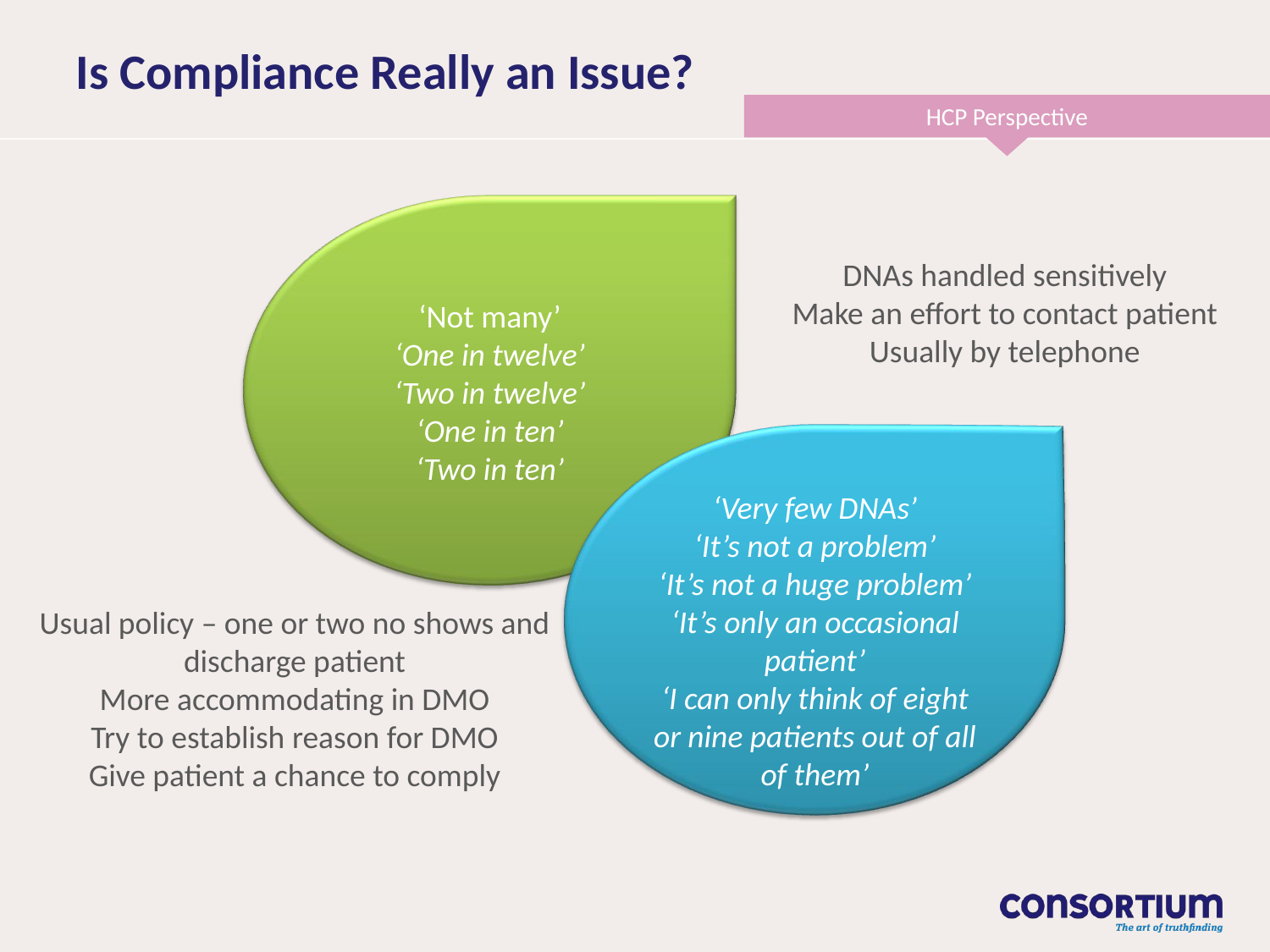

# Is Compliance Really an Issue?
HCP Perspective
‘Not many’
‘One in twelve’
‘Two in twelve’
‘One in ten’
‘Two in ten’
DNAs handled sensitively
Make an effort to contact patient
Usually by telephone
‘Very few DNAs’
‘It’s not a problem’
‘It’s not a huge problem’
‘It’s only an occasional patient’
‘I can only think of eight or nine patients out of all of them’
Usual policy – one or two no shows and discharge patient
More accommodating in DMO
Try to establish reason for DMO
Give patient a chance to comply

## Slide 18
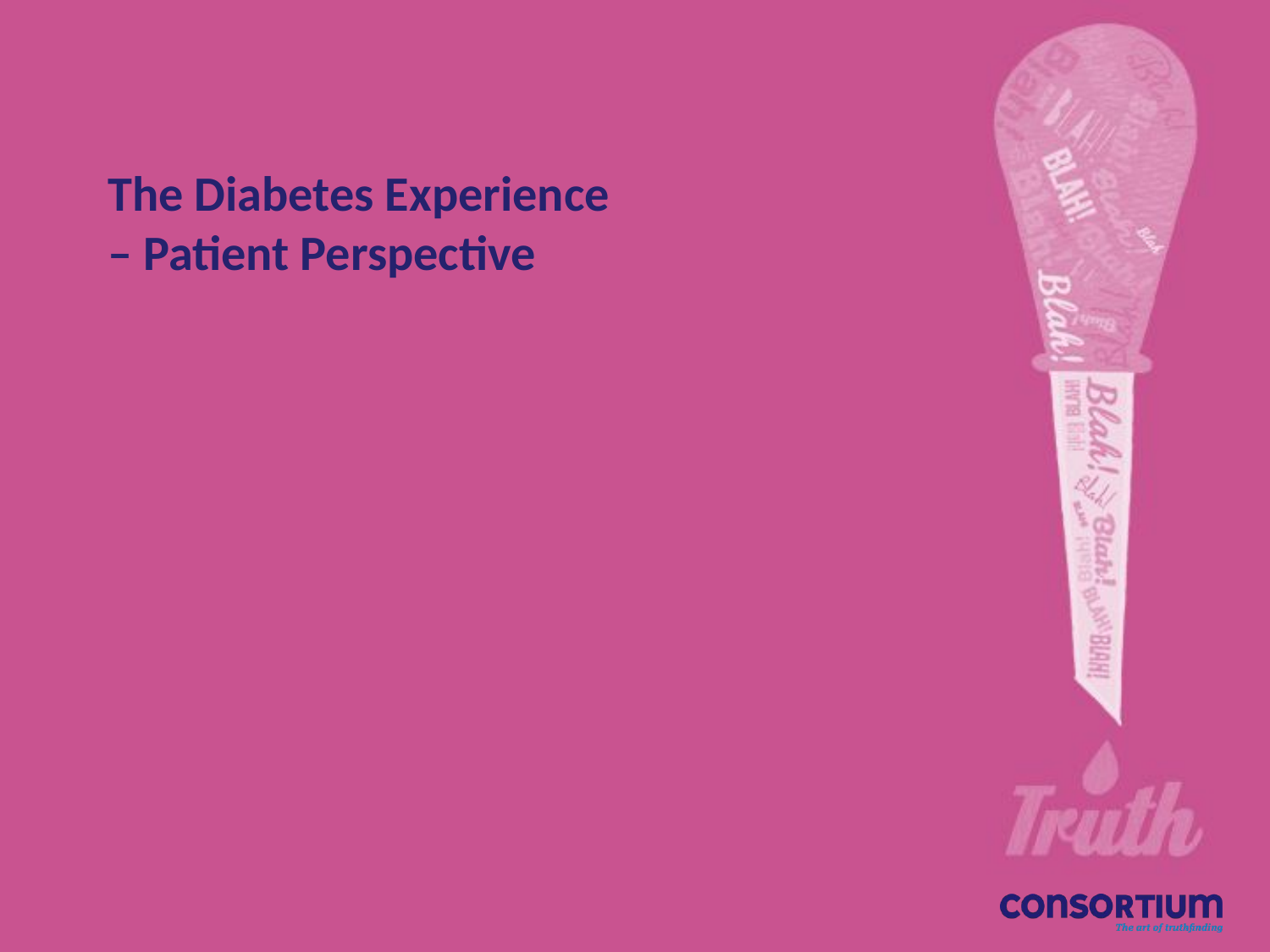

# The Diabetes Experience – Patient Perspective

## Slide 19
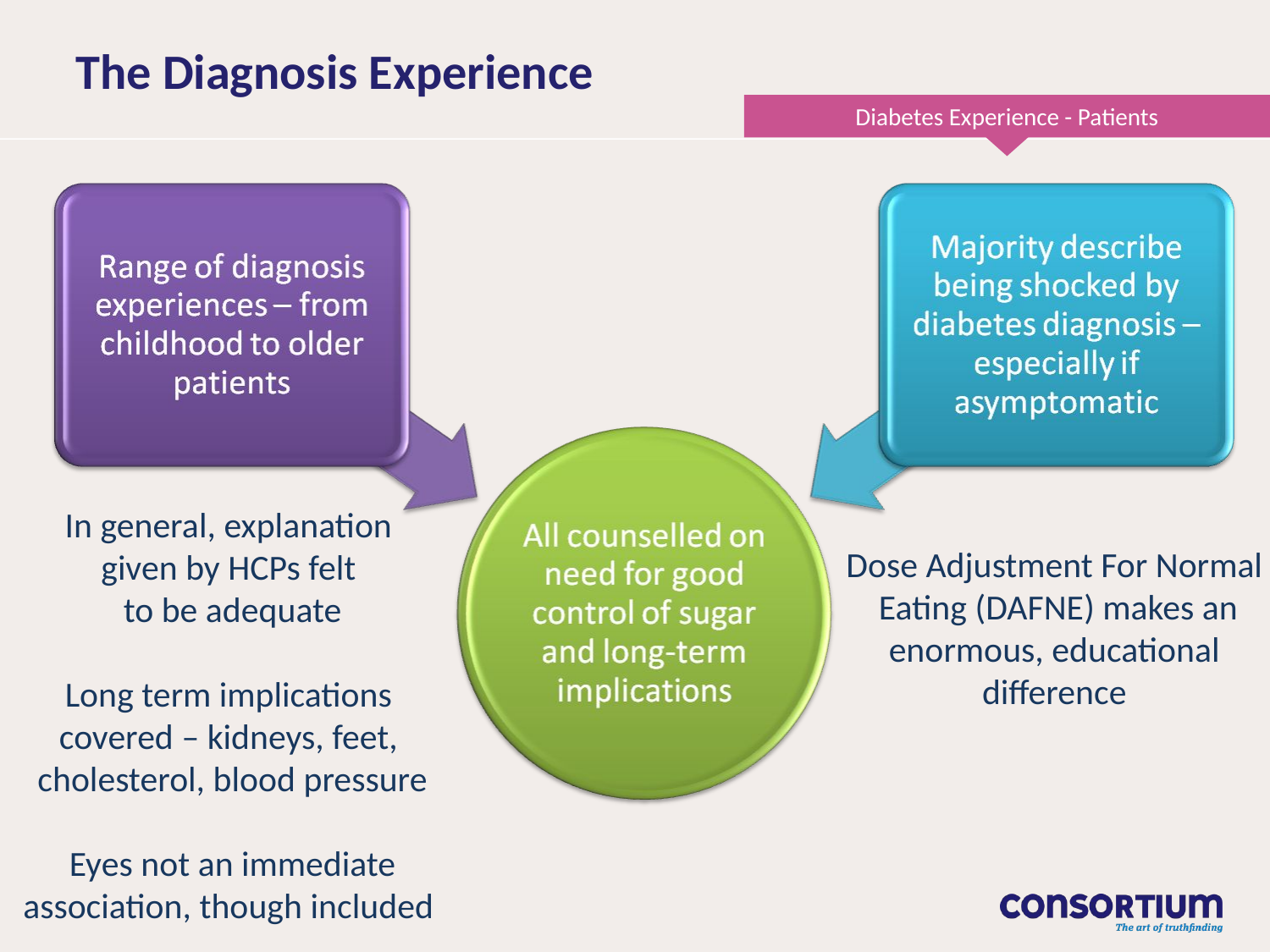

# The Diagnosis Experience
Diabetes Experience - Patients
In general, explanation
given by HCPs felt
to be adequate
Long term implications
covered – kidneys, feet,
cholesterol, blood pressure
Eyes not an immediate
association, though included
Dose Adjustment For Normal
Eating (DAFNE) makes an
enormous, educational
difference

## Slide 20
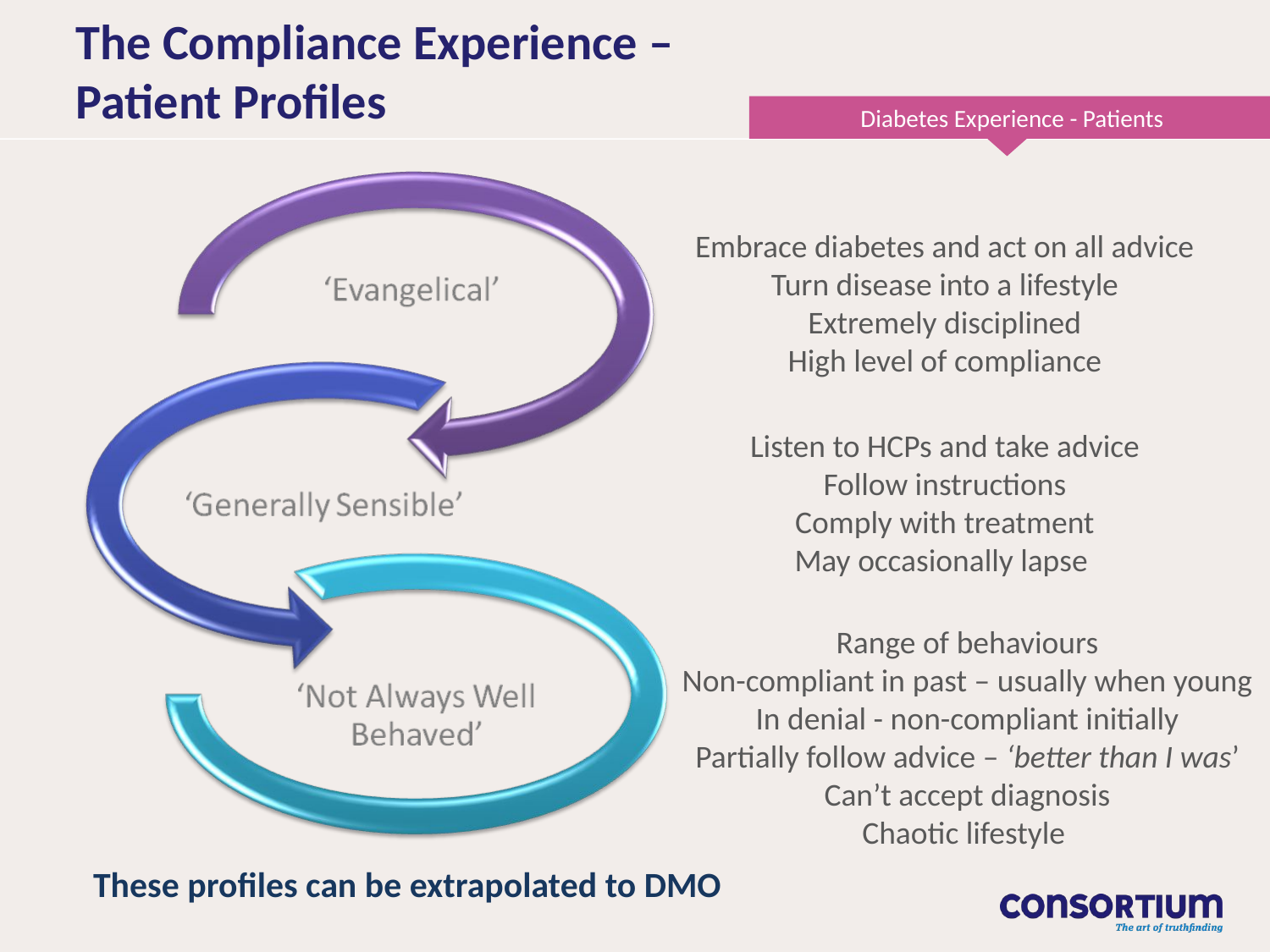

# The Compliance Experience – Patient Profiles
Diabetes Experience - Patients
Embrace diabetes and act on all advice
Turn disease into a lifestyle
Extremely disciplined
High level of compliance
Listen to HCPs and take advice
Follow instructions
Comply with treatment
May occasionally lapse
Range of behaviours
Non-compliant in past – usually when young
In denial - non-compliant initially
Partially follow advice – ‘better than I was’
Can’t accept diagnosis
Chaotic lifestyle
These profiles can be extrapolated to DMO

## Slide 21
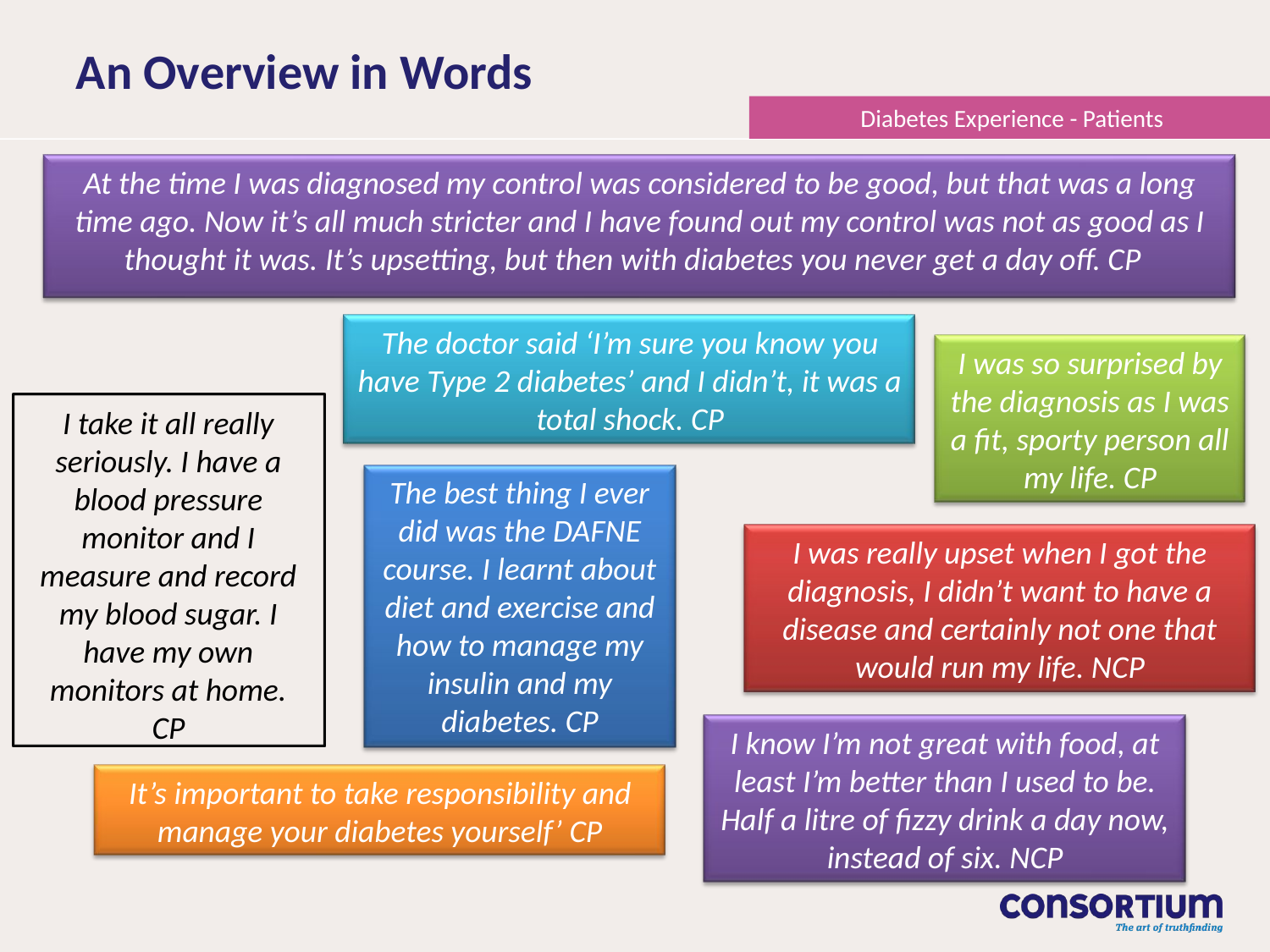

# An Overview in Words
Diabetes Experience - Patients
At the time I was diagnosed my control was considered to be good, but that was a long time ago. Now it’s all much stricter and I have found out my control was not as good as I thought it was. It’s upsetting, but then with diabetes you never get a day off. CP
The doctor said ‘I’m sure you know you have Type 2 diabetes’ and I didn’t, it was a total shock. CP
I was so surprised by the diagnosis as I was a fit, sporty person all my life. CP
I take it all really seriously. I have a blood pressure monitor and I measure and record my blood sugar. I have my own monitors at home. CP
The best thing I ever did was the DAFNE course. I learnt about diet and exercise and how to manage my insulin and my diabetes. CP
I was really upset when I got the diagnosis, I didn’t want to have a disease and certainly not one that would run my life. NCP
I know I’m not great with food, at least I’m better than I used to be. Half a litre of fizzy drink a day now, instead of six. NCP
It’s important to take responsibility and manage your diabetes yourself’ CP

## Slide 22
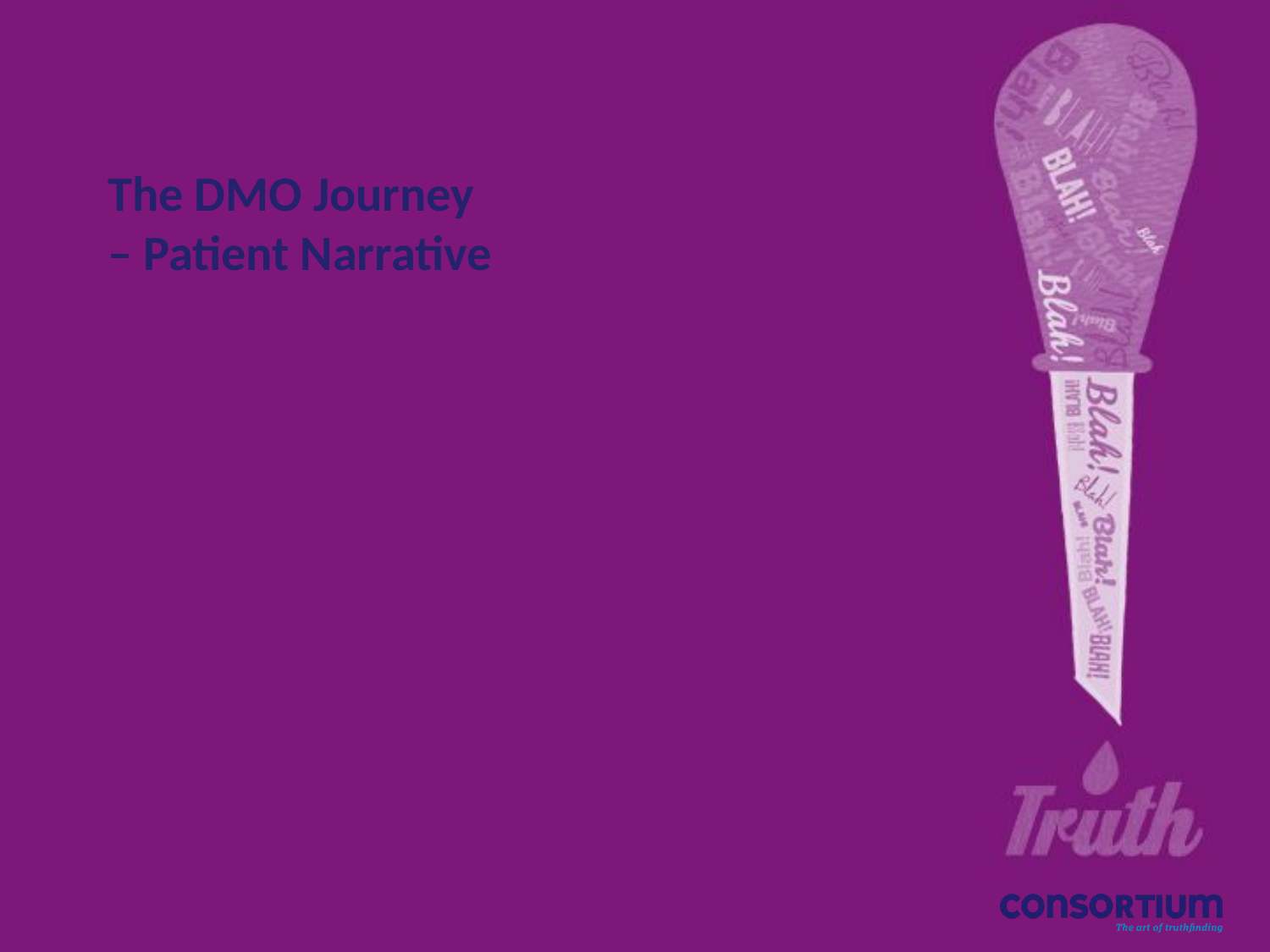

# The DMO Journey – Patient Narrative

## Slide 23
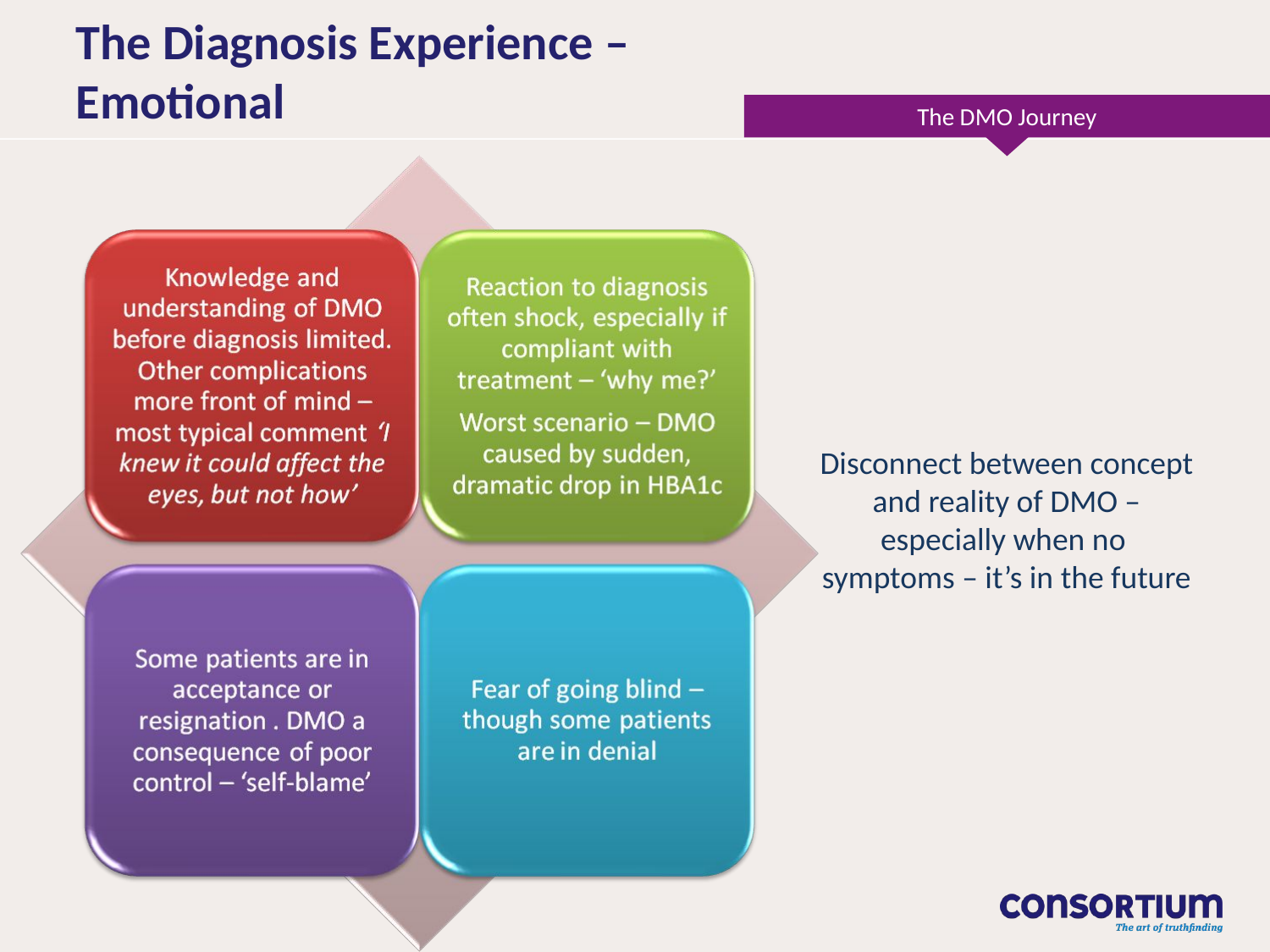

# The Diagnosis Experience – Emotional
The DMO Journey
Disconnect between concept
 and reality of DMO –
especially when no
symptoms – it’s in the future

## Slide 24
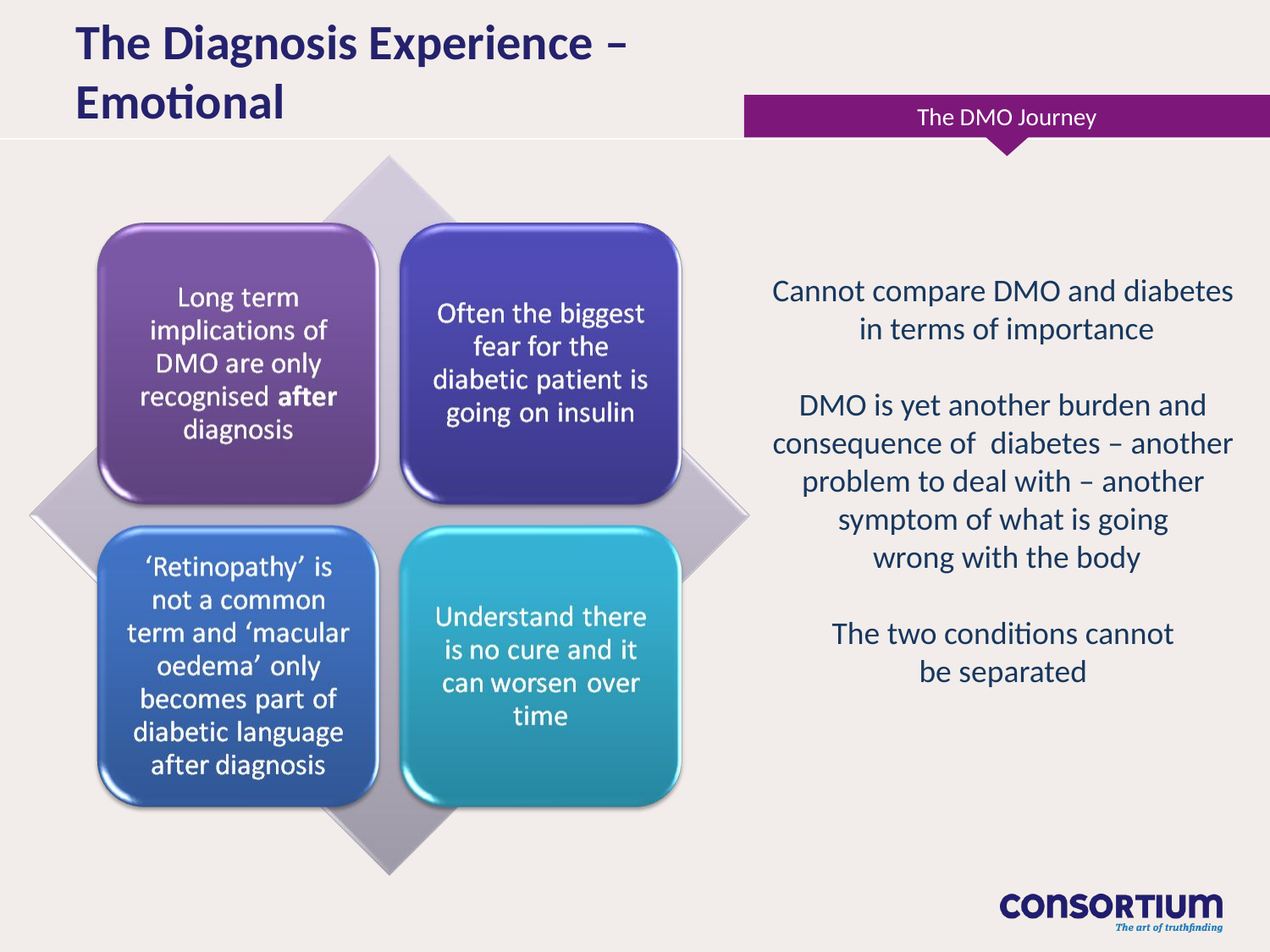

# The Diagnosis Experience – Emotional
The DMO Journey
Cannot compare DMO and diabetes
in terms of importance
DMO is yet another burden and
consequence of diabetes – another
problem to deal with – another
symptom of what is going
wrong with the body
The two conditions cannot
be separated

## Slide 25
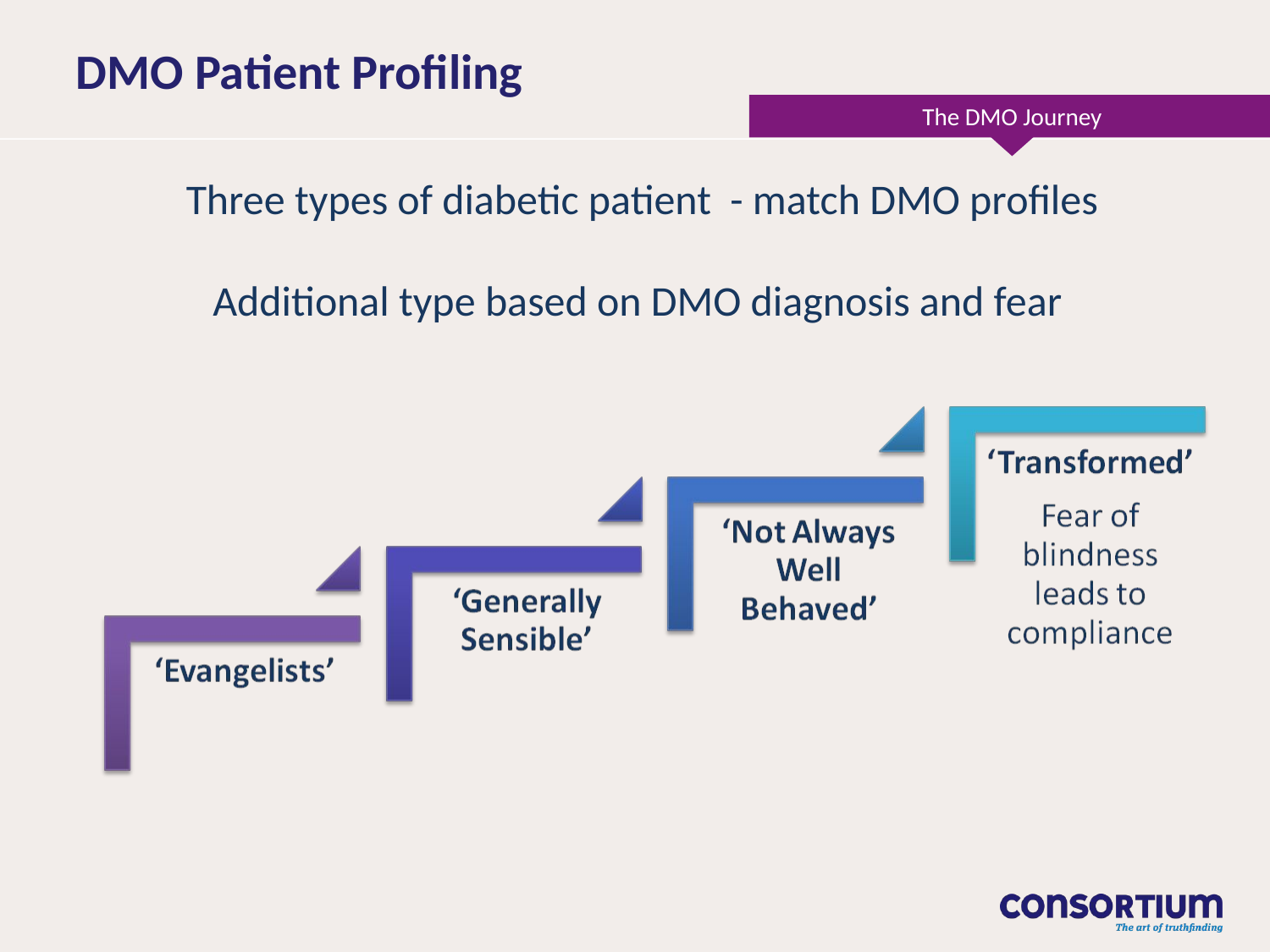

# DMO Patient Profiling
The DMO Journey
Three types of diabetic patient - match DMO profiles
Additional type based on DMO diagnosis and fear

## Slide 26
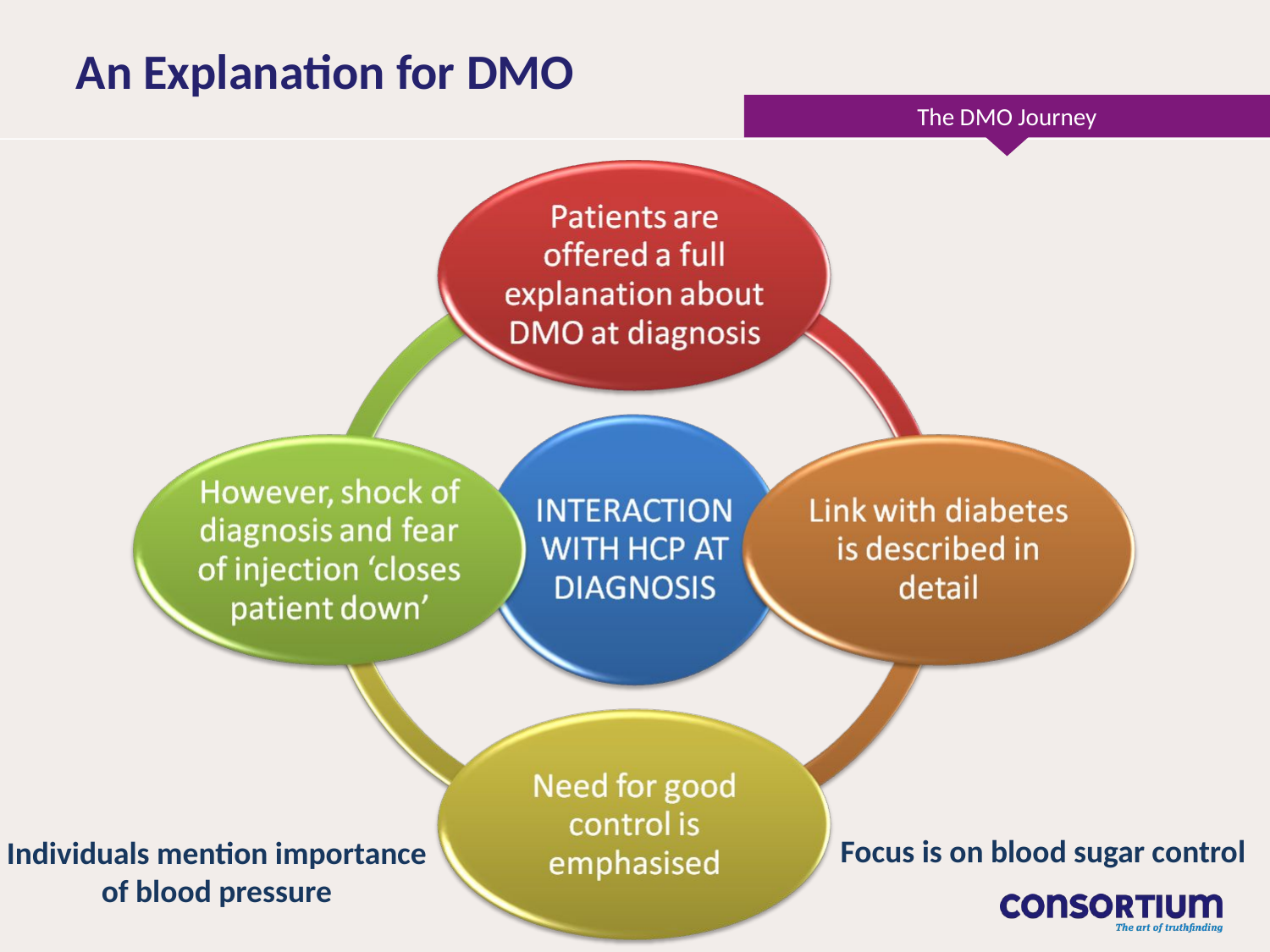

# An Explanation for DMO
The DMO Journey
Focus is on blood sugar control
Individuals mention importance
of blood pressure

## Slide 27
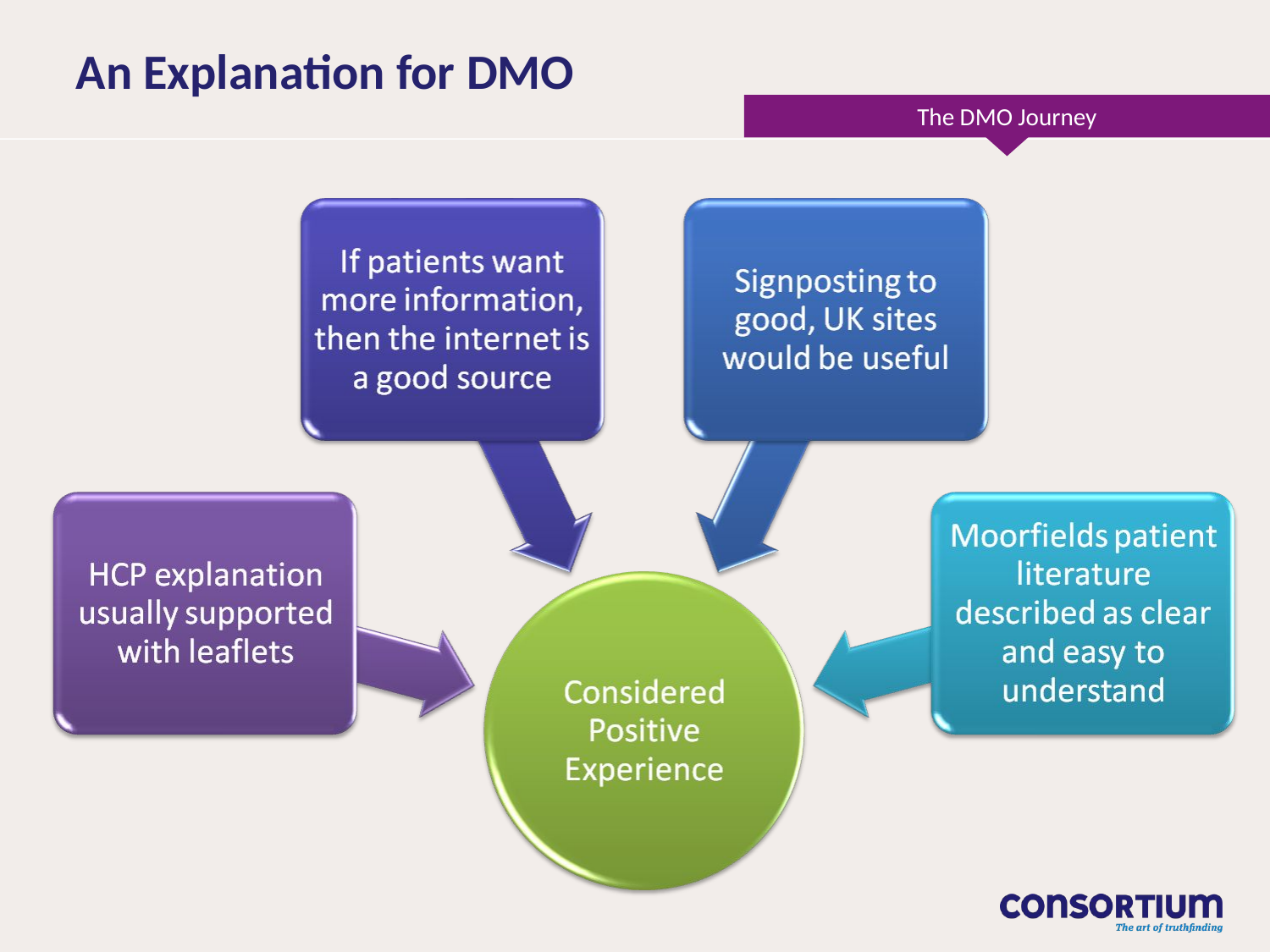

# An Explanation for DMO
The DMO Journey

## Slide 28
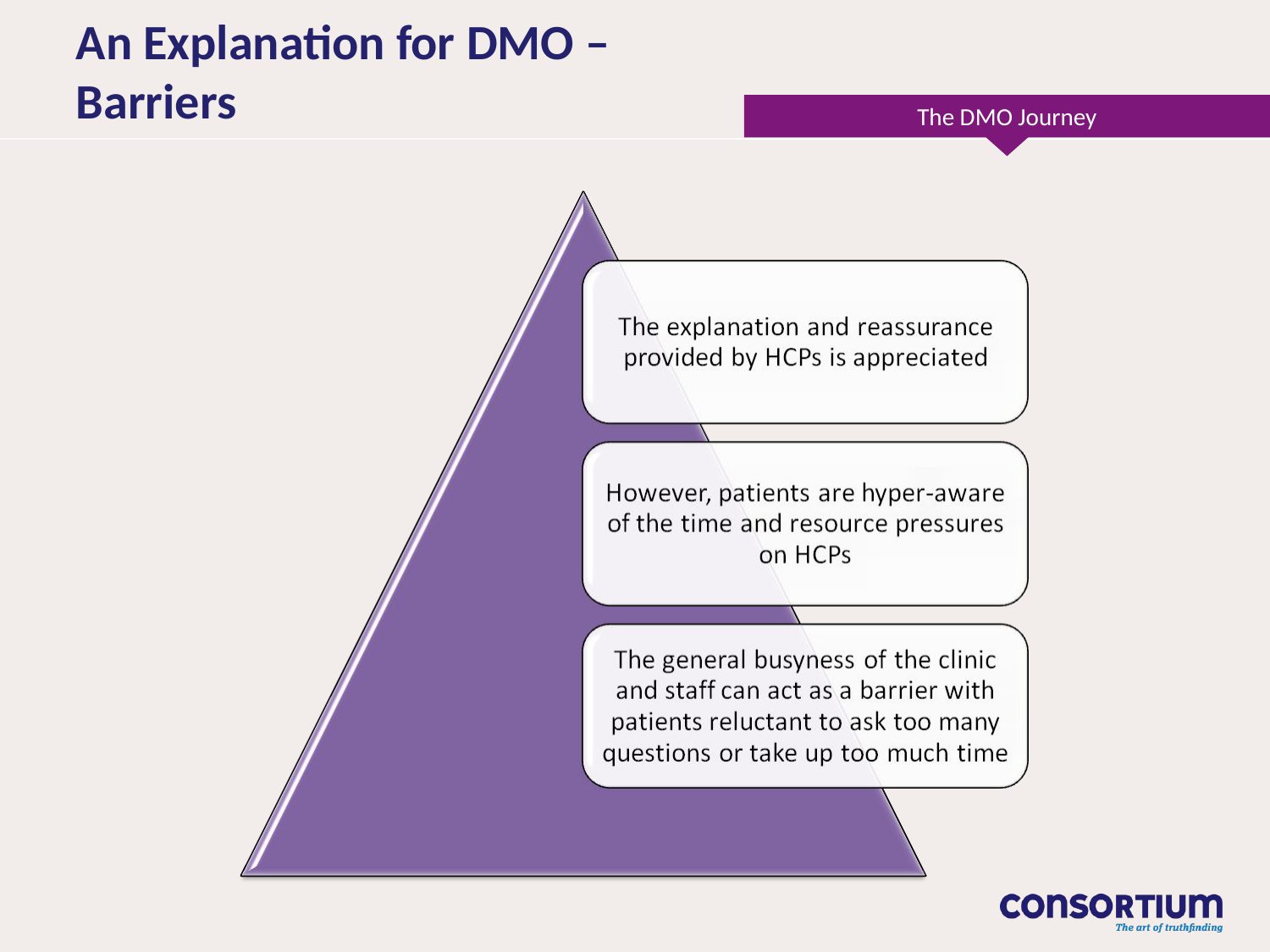

# An Explanation for DMO – Barriers
The DMO Journey

## Slide 29
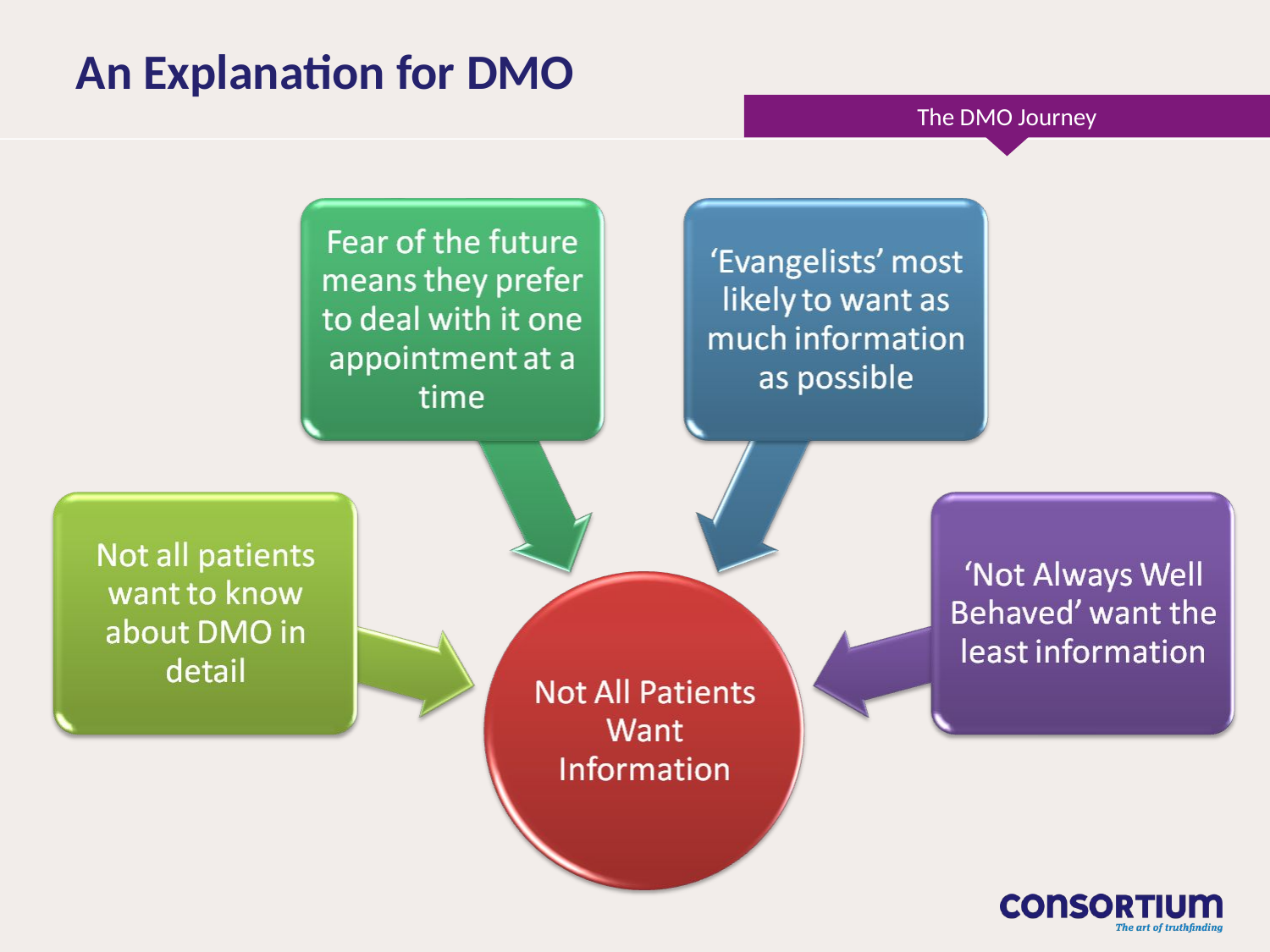

# An Explanation for DMO
The DMO Journey

## Slide 30
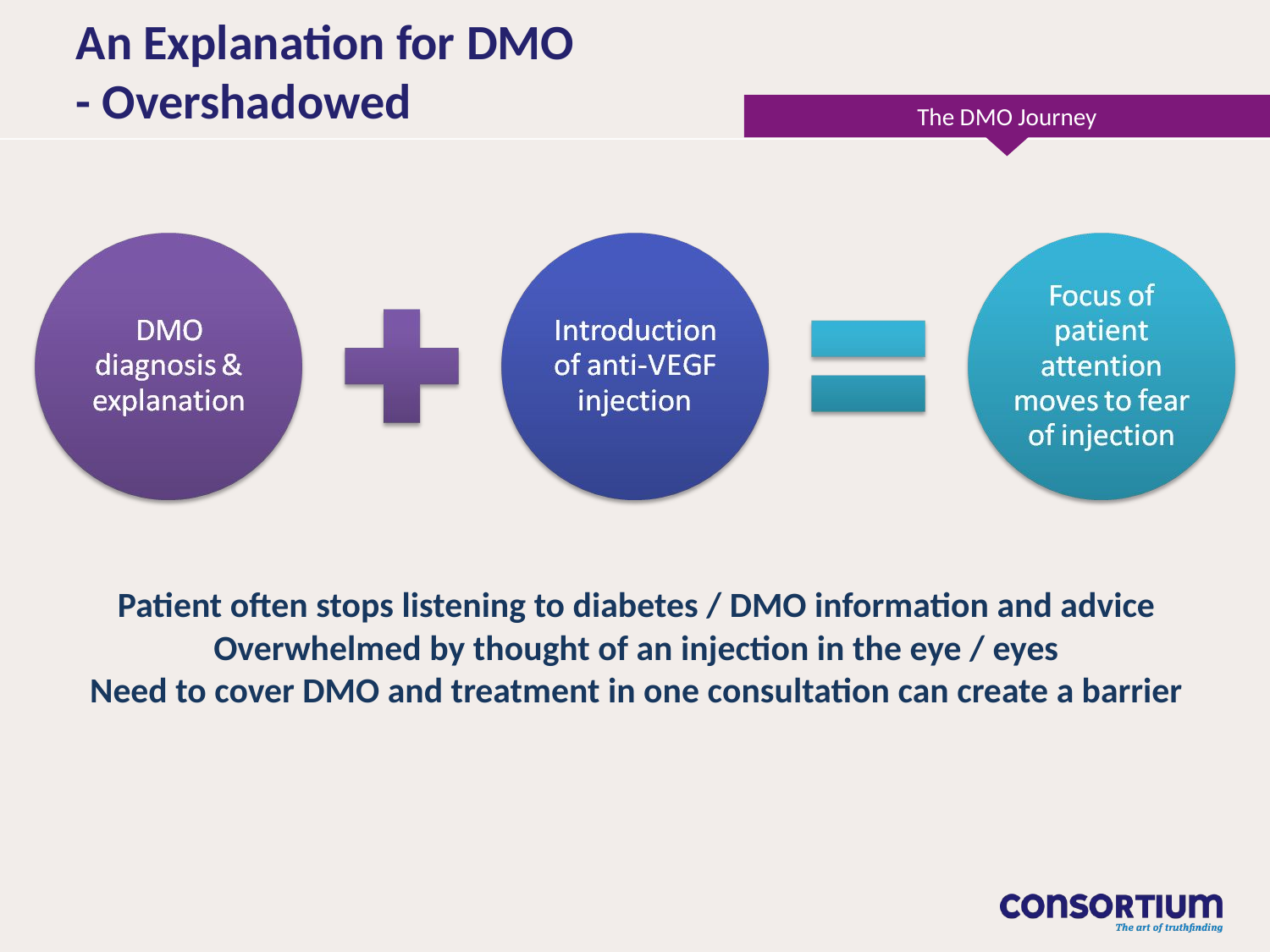

# An Explanation for DMO - Overshadowed
The DMO Journey
Patient often stops listening to diabetes / DMO information and advice
Overwhelmed by thought of an injection in the eye / eyes
Need to cover DMO and treatment in one consultation can create a barrier

## Slide 31
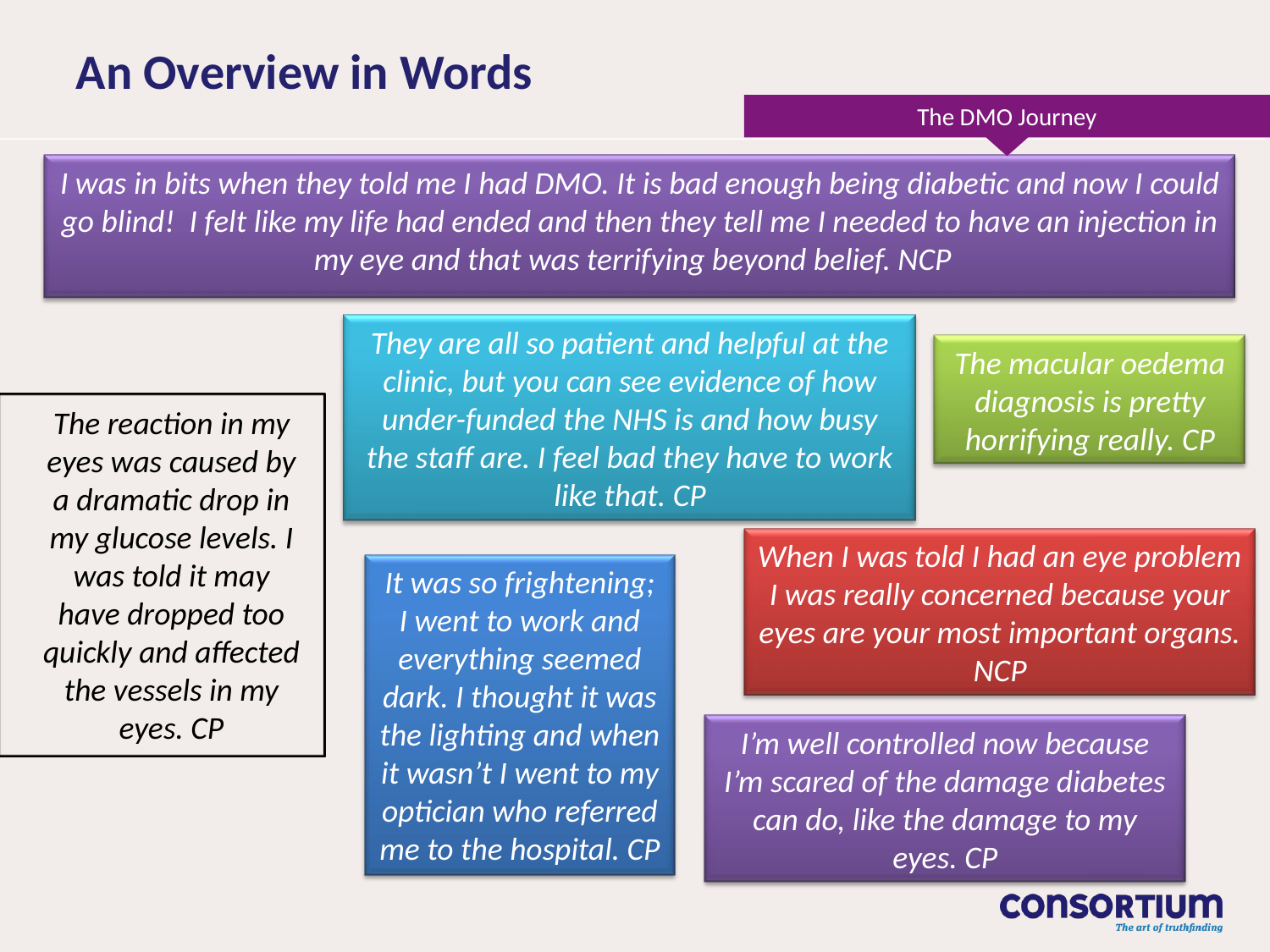

# An Overview in Words
The DMO Journey
I was in bits when they told me I had DMO. It is bad enough being diabetic and now I could go blind! I felt like my life had ended and then they tell me I needed to have an injection in my eye and that was terrifying beyond belief. NCP
They are all so patient and helpful at the clinic, but you can see evidence of how under-funded the NHS is and how busy the staff are. I feel bad they have to work like that. CP
The macular oedema diagnosis is pretty horrifying really. CP
The reaction in my eyes was caused by a dramatic drop in my glucose levels. I was told it may have dropped too quickly and affected the vessels in my eyes. CP
When I was told I had an eye problem I was really concerned because your eyes are your most important organs. NCP
It was so frightening; I went to work and everything seemed dark. I thought it was the lighting and when it wasn’t I went to my optician who referred me to the hospital. CP
I’m well controlled now because I’m scared of the damage diabetes can do, like the damage to my eyes. CP

## Slide 32
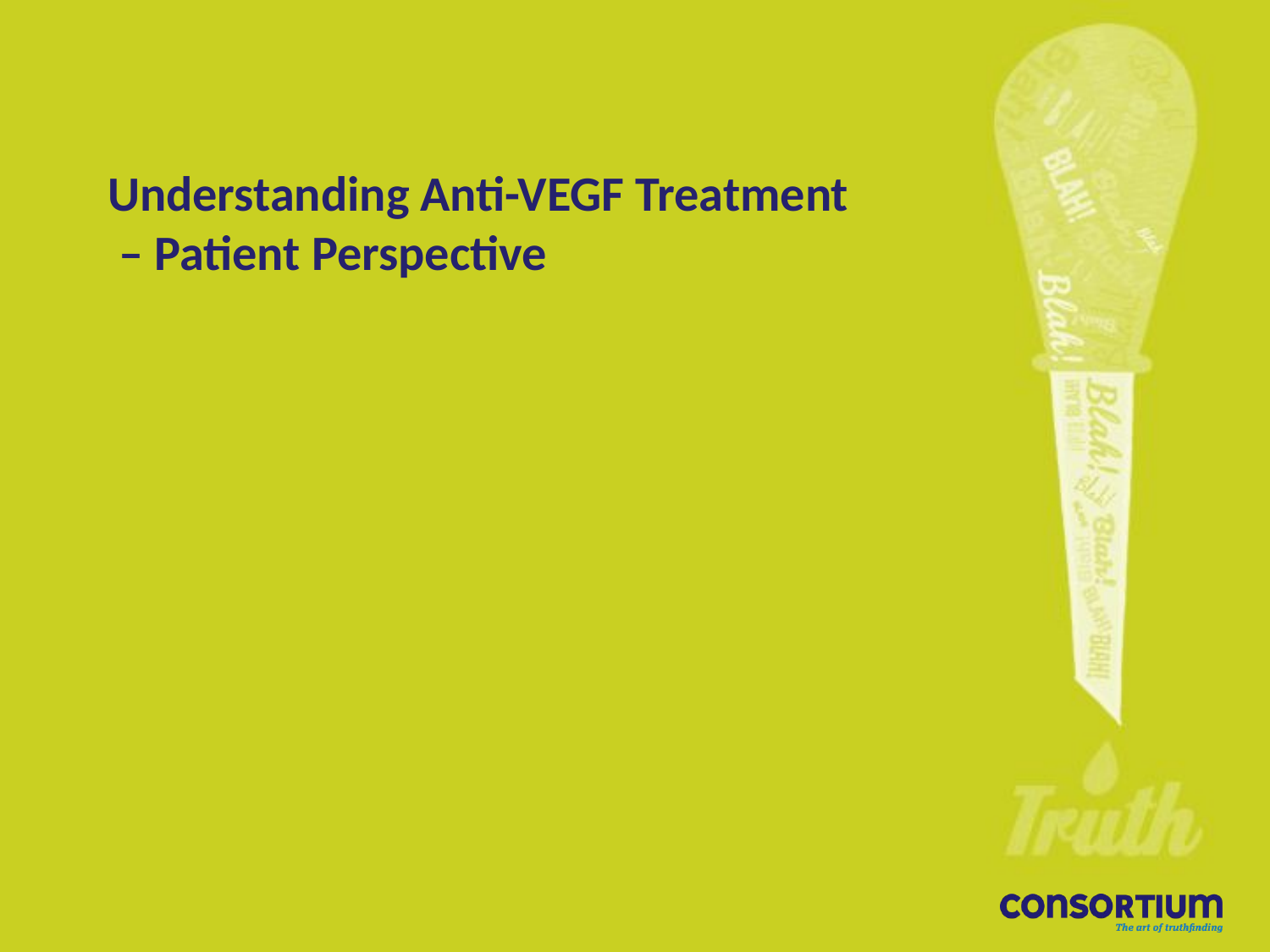

# Understanding Anti-VEGF Treatment – Patient Perspective

## Slide 33
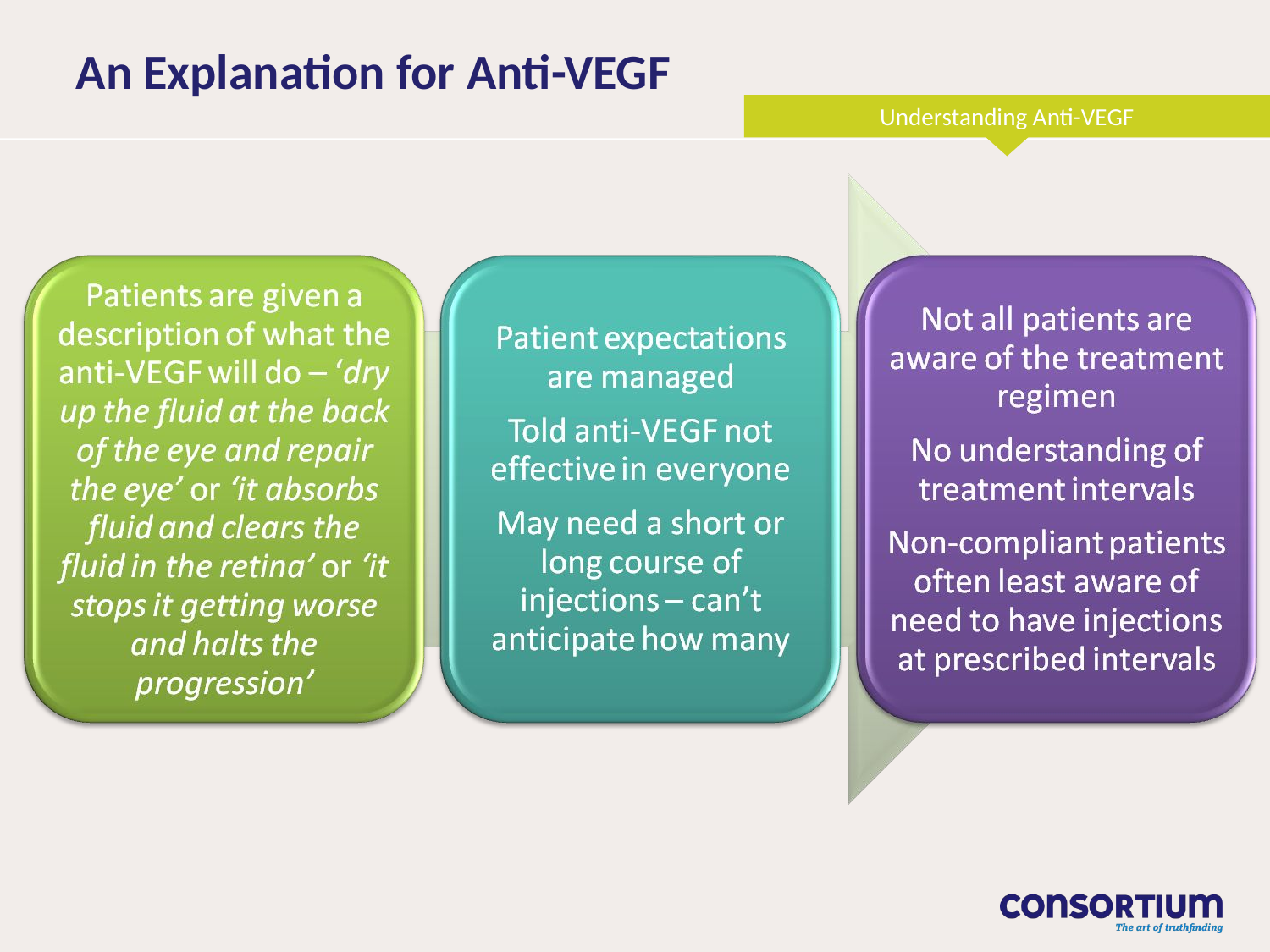

# An Explanation for Anti-VEGF
Understanding Anti-VEGF

## Slide 34
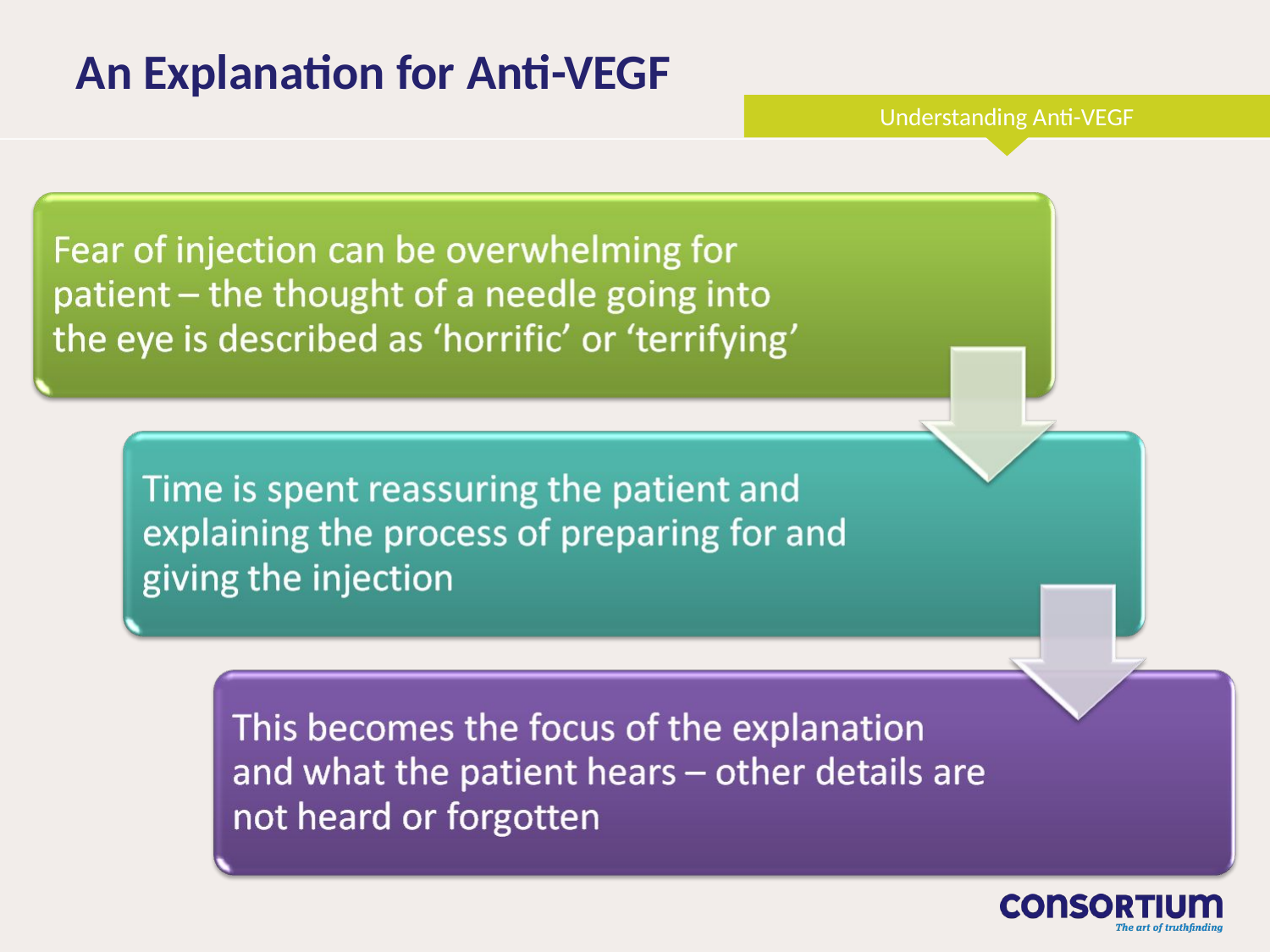

# An Explanation for Anti-VEGF
Understanding Anti-VEGF

## Slide 35
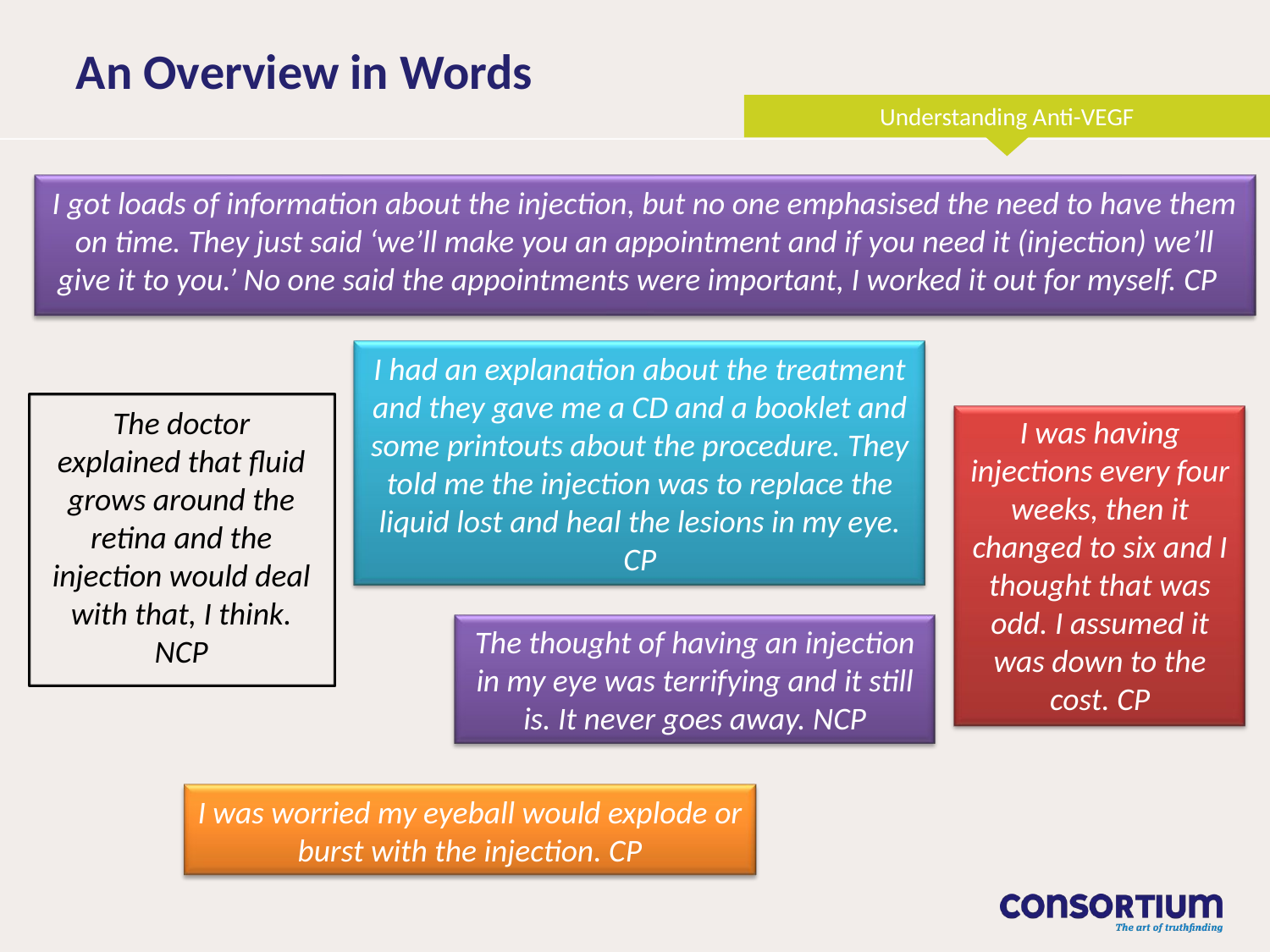

# An Overview in Words
Understanding Anti-VEGF
I got loads of information about the injection, but no one emphasised the need to have them on time. They just said ‘we’ll make you an appointment and if you need it (injection) we’ll give it to you.’ No one said the appointments were important, I worked it out for myself. CP
I had an explanation about the treatment and they gave me a CD and a booklet and some printouts about the procedure. They told me the injection was to replace the liquid lost and heal the lesions in my eye. CP
The doctor explained that fluid grows around the retina and the injection would deal with that, I think. NCP
I was having injections every four weeks, then it changed to six and I thought that was odd. I assumed it was down to the cost. CP
The thought of having an injection in my eye was terrifying and it still is. It never goes away. NCP
I was worried my eyeball would explode or burst with the injection. CP

## Slide 36
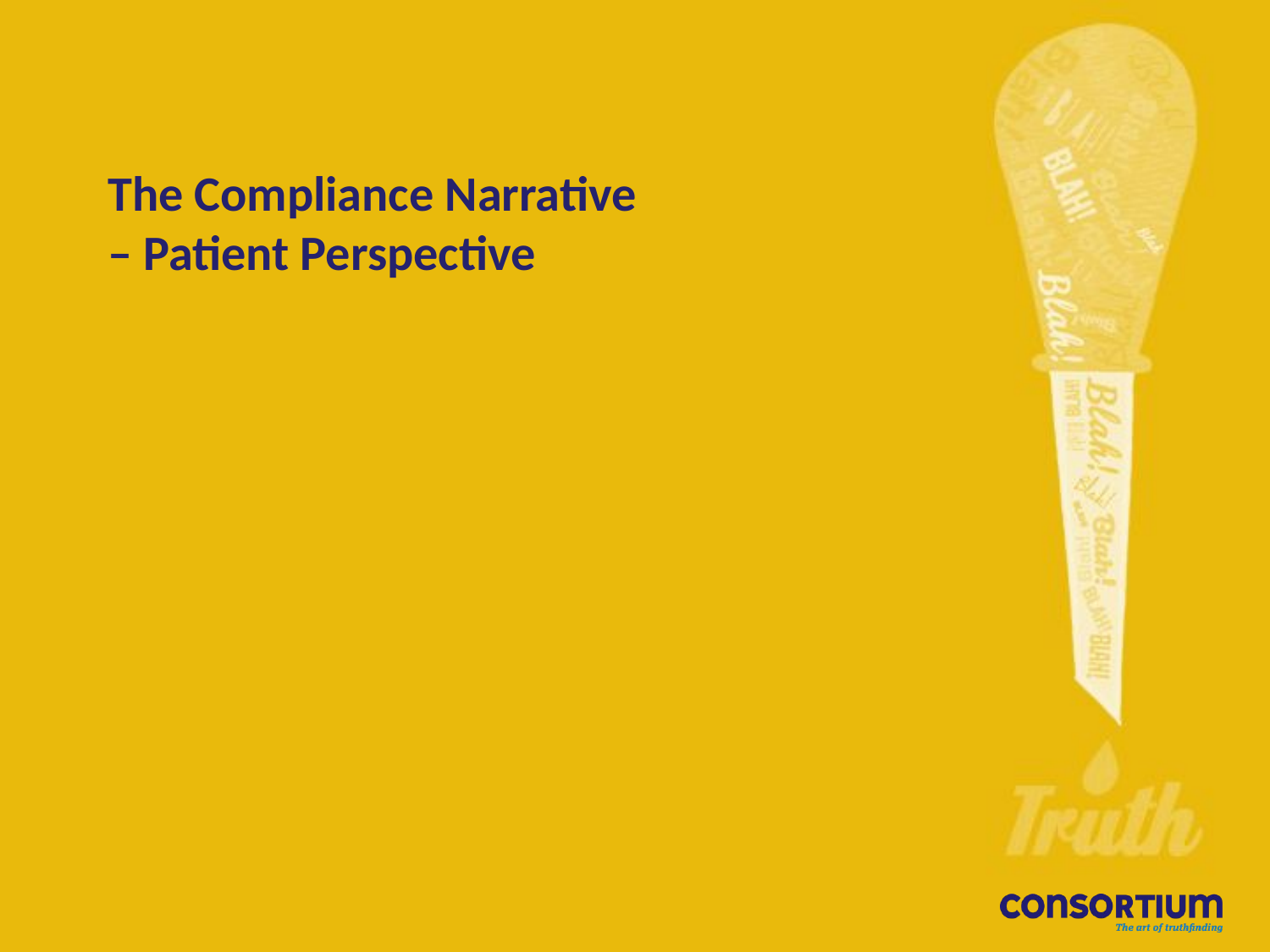

# The Compliance Narrative – Patient Perspective

## Slide 37
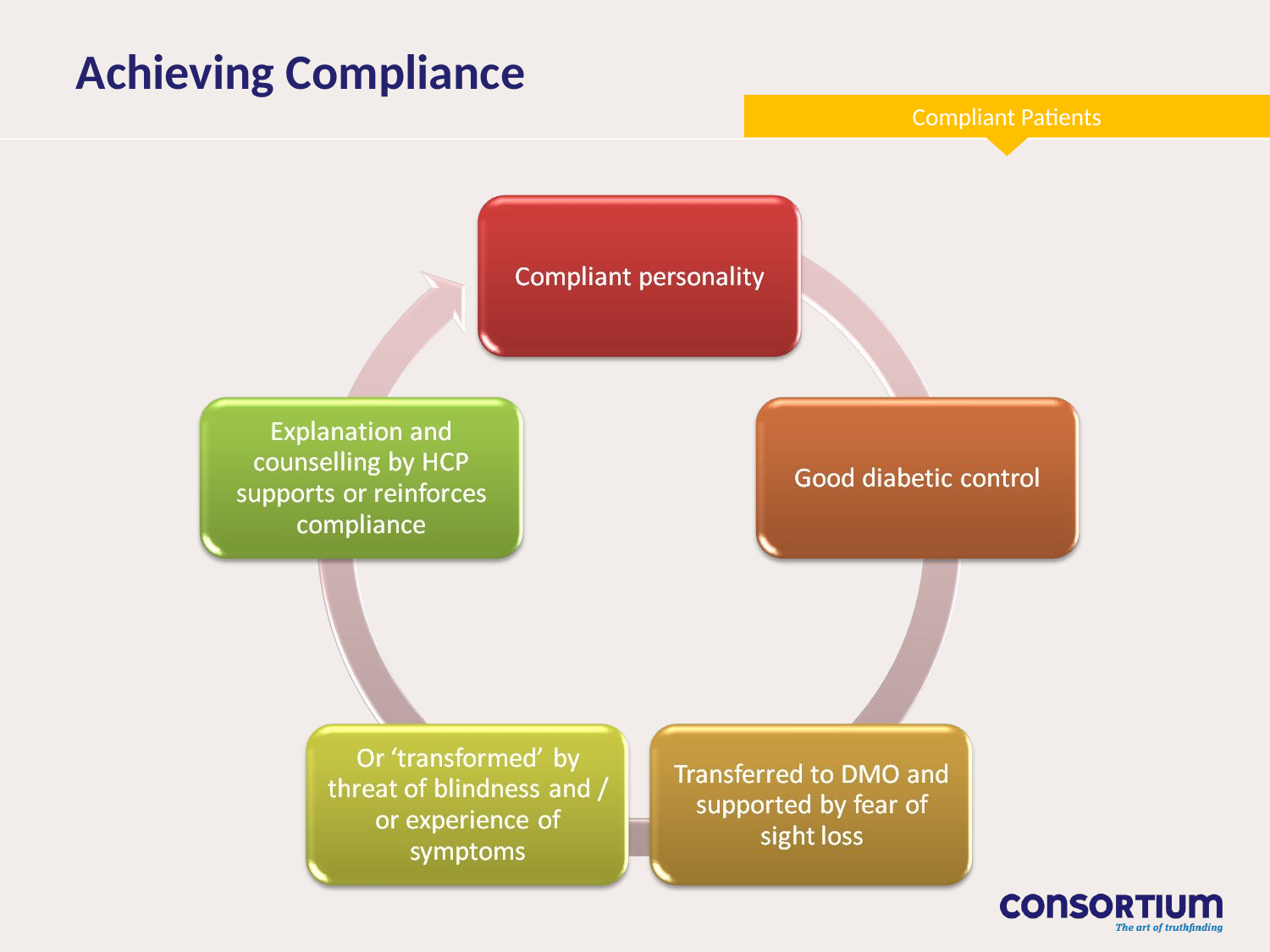

# Achieving Compliance
Compliant Patients

## Slide 38
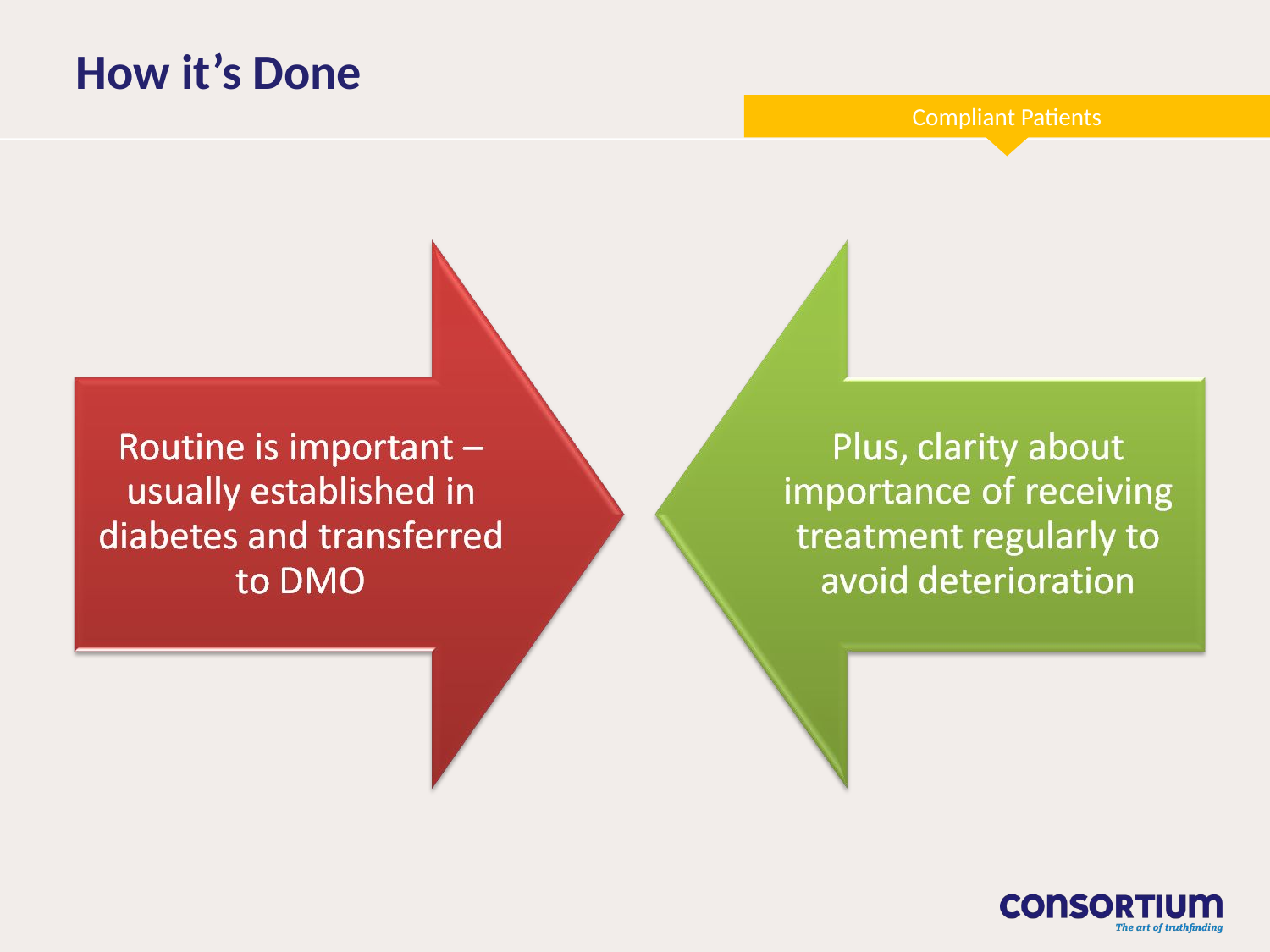

# How it’s Done
Compliant Patients

## Slide 39
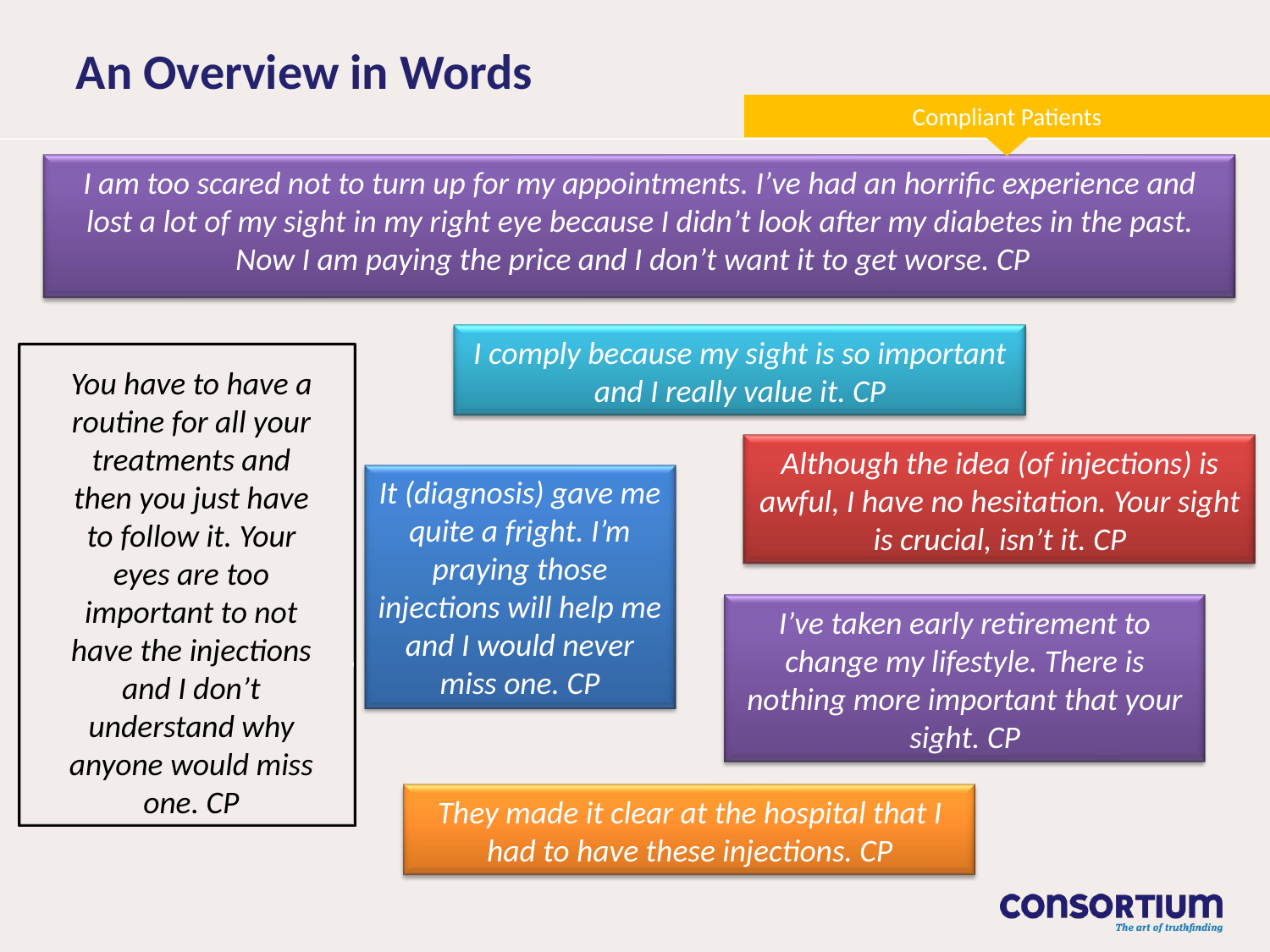

# An Overview in Words
Compliant Patients
I am too scared not to turn up for my appointments. I’ve had an horrific experience and lost a lot of my sight in my right eye because I didn’t look after my diabetes in the past. Now I am paying the price and I don’t want it to get worse. CP
I comply because my sight is so important and I really value it. CP
You have to have a routine for all your treatments and then you just have to follow it. Your eyes are too important to not have the injections and I don’t understand why anyone would miss one. CP
Although the idea (of injections) is awful, I have no hesitation. Your sight is crucial, isn’t it. CP
It (diagnosis) gave me quite a fright. I’m praying those injections will help me and I would never miss one. CP
I’ve taken early retirement to change my lifestyle. There is nothing more important that your sight. CP
They made it clear at the hospital that I had to have these injections. CP

## Slide 40
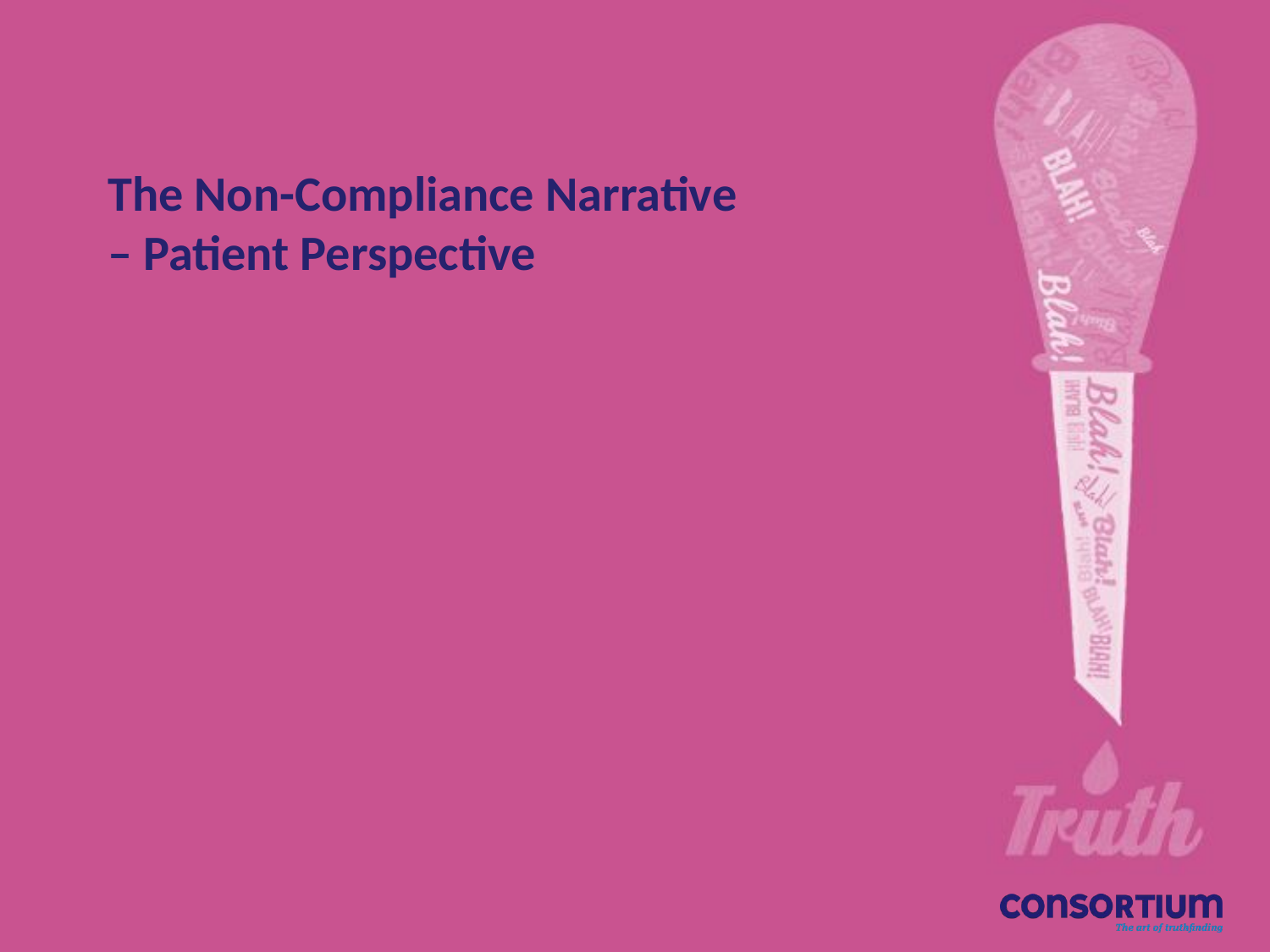

# The Non-Compliance Narrative – Patient Perspective

## Slide 41
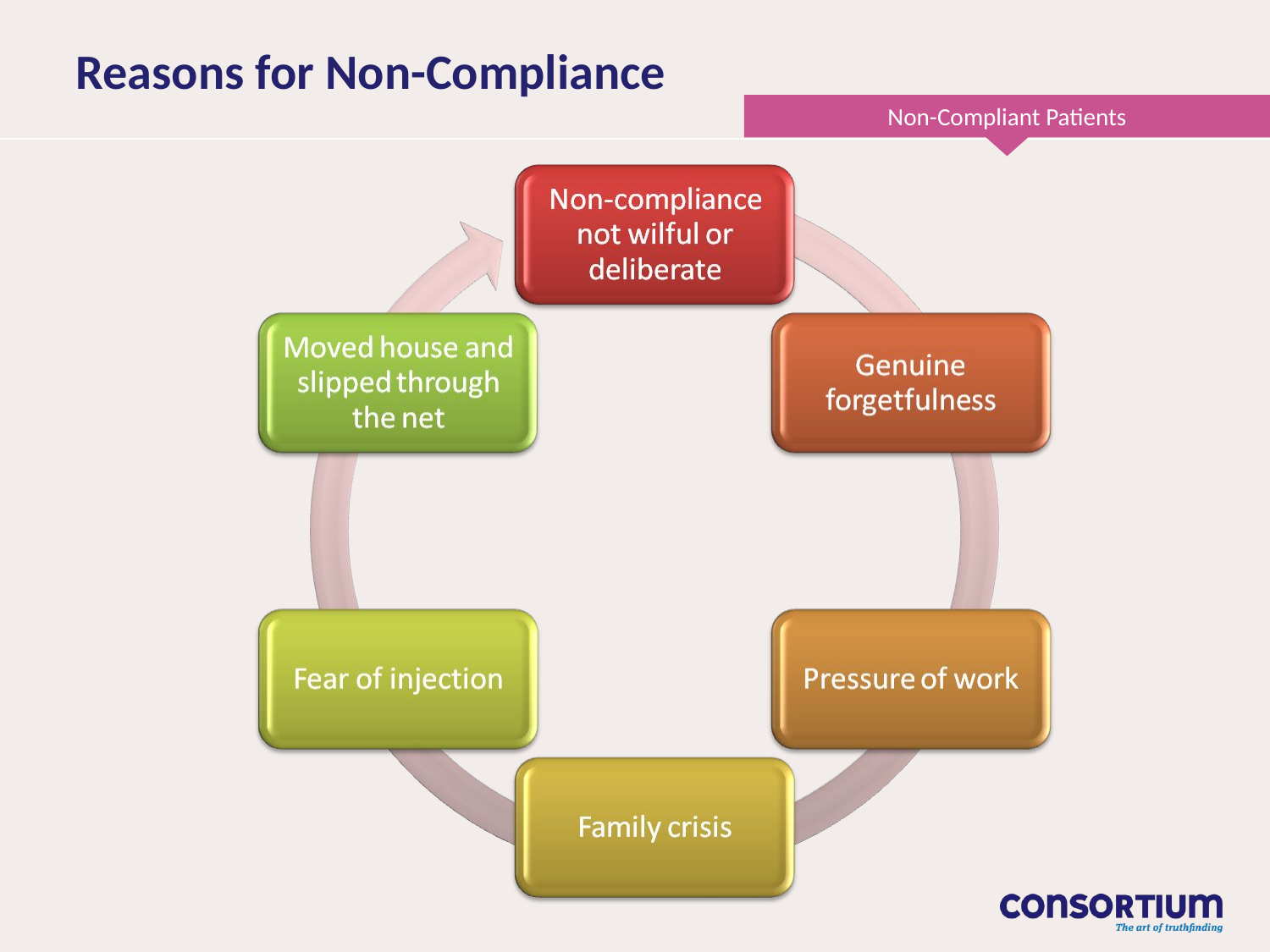

# Reasons for Non-Compliance
Non-Compliant Patients

## Slide 42
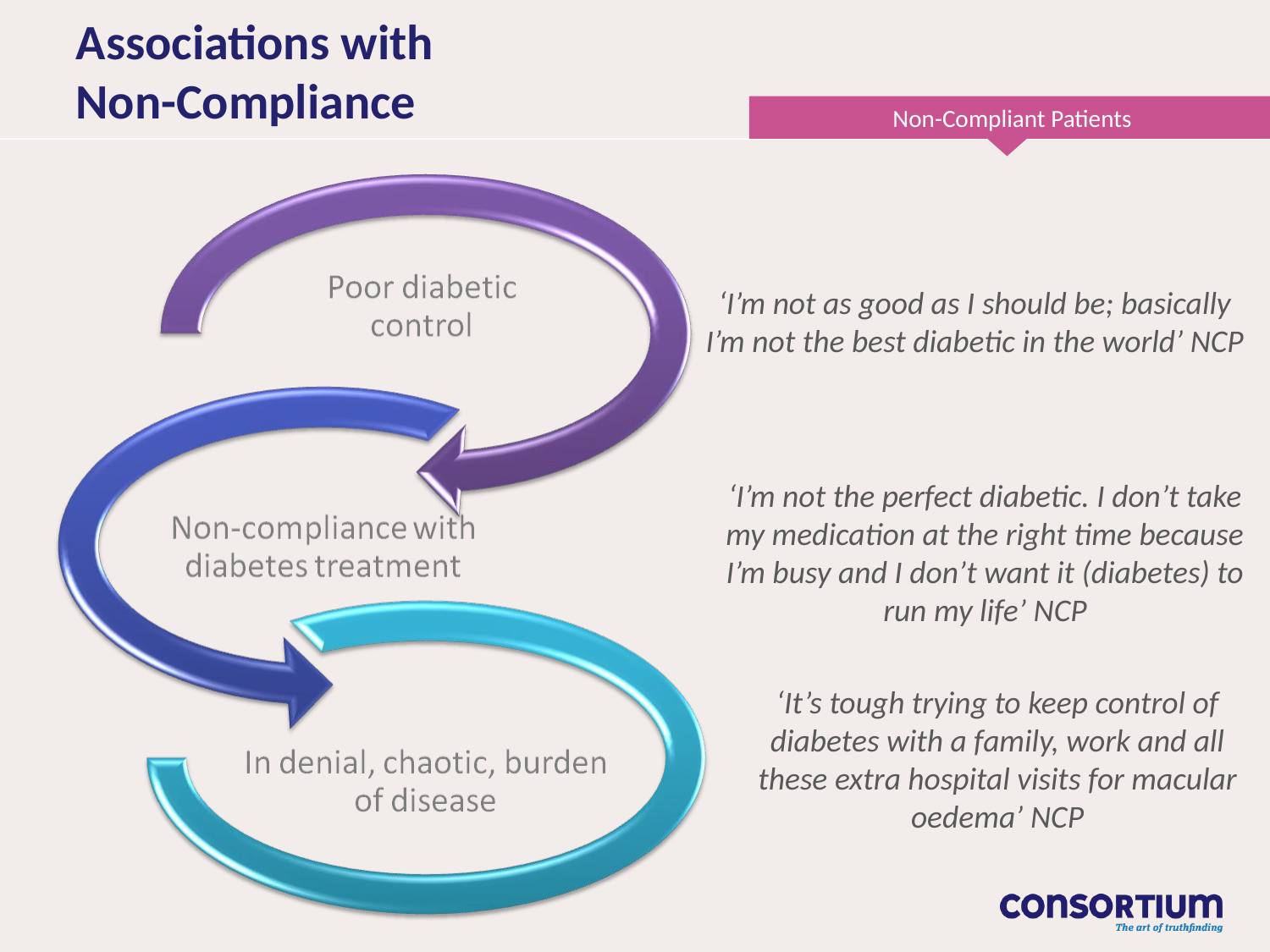

# Associations with Non-Compliance
Non-Compliant Patients
‘I’m not as good as I should be; basically I’m not the best diabetic in the world’ NCP
‘I’m not the perfect diabetic. I don’t take my medication at the right time because I’m busy and I don’t want it (diabetes) to run my life’ NCP
‘It’s tough trying to keep control of diabetes with a family, work and all these extra hospital visits for macular oedema’ NCP

## Slide 43
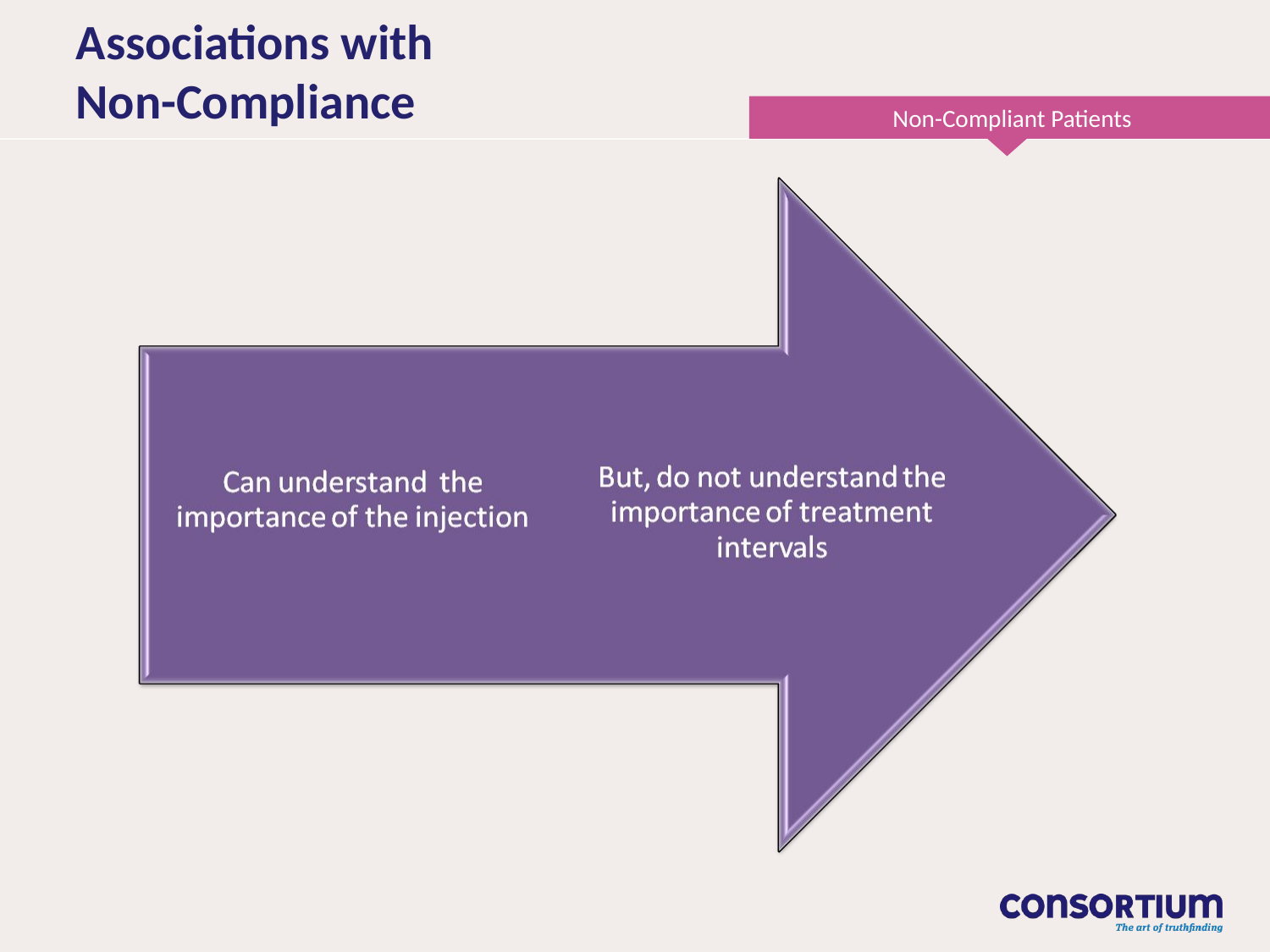

# Associations with Non-Compliance
Non-Compliant Patients

## Slide 44
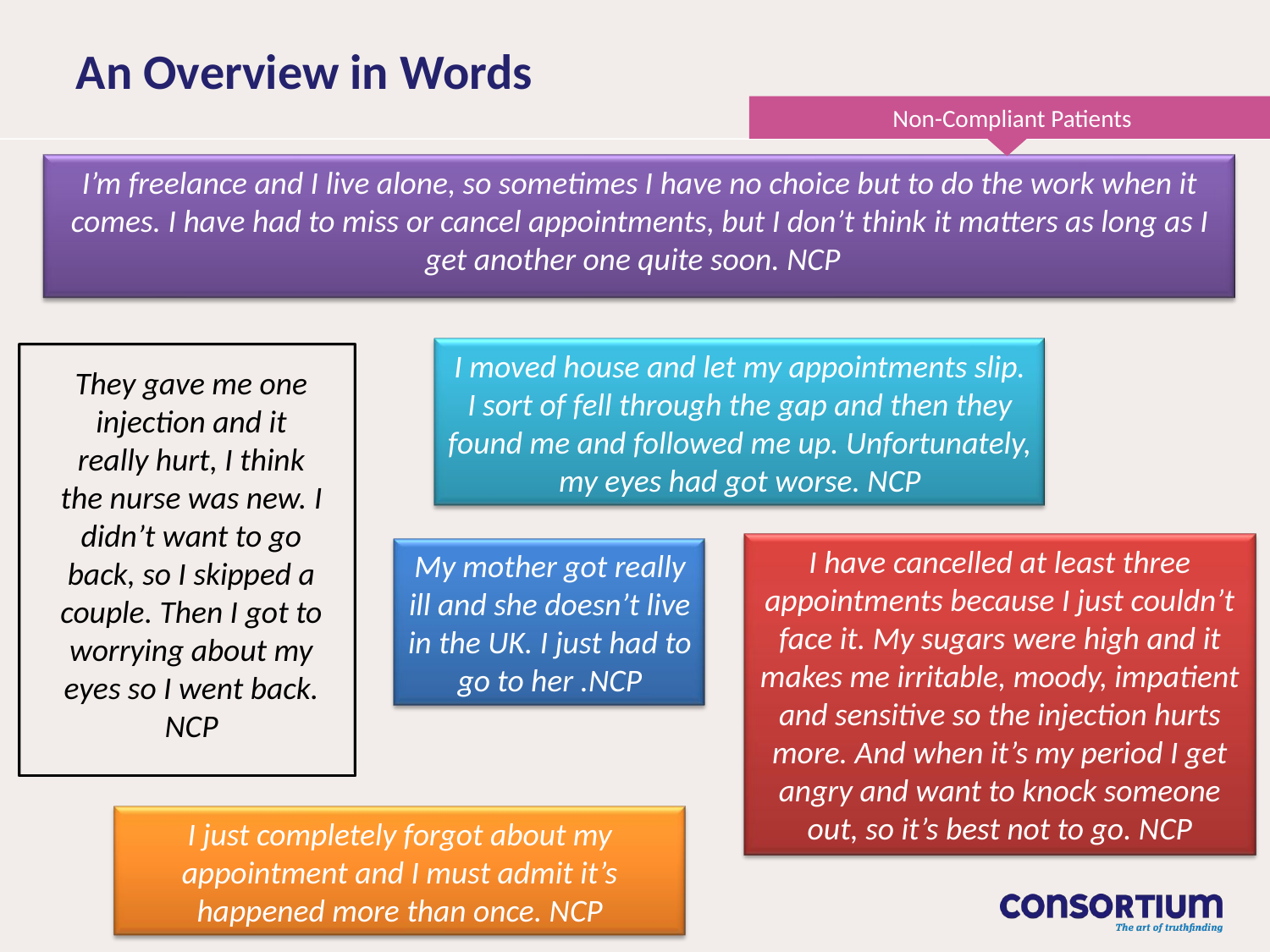

# An Overview in Words
Non-Compliant Patients
I’m freelance and I live alone, so sometimes I have no choice but to do the work when it comes. I have had to miss or cancel appointments, but I don’t think it matters as long as I get another one quite soon. NCP
I moved house and let my appointments slip. I sort of fell through the gap and then they found me and followed me up. Unfortunately, my eyes had got worse. NCP
They gave me one injection and it really hurt, I think the nurse was new. I didn’t want to go back, so I skipped a couple. Then I got to worrying about my eyes so I went back. NCP
I have cancelled at least three appointments because I just couldn’t face it. My sugars were high and it makes me irritable, moody, impatient and sensitive so the injection hurts more. And when it’s my period I get angry and want to knock someone out, so it’s best not to go. NCP
My mother got really ill and she doesn’t live in the UK. I just had to go to her .NCP
I just completely forgot about my appointment and I must admit it’s happened more than once. NCP

## Slide 45
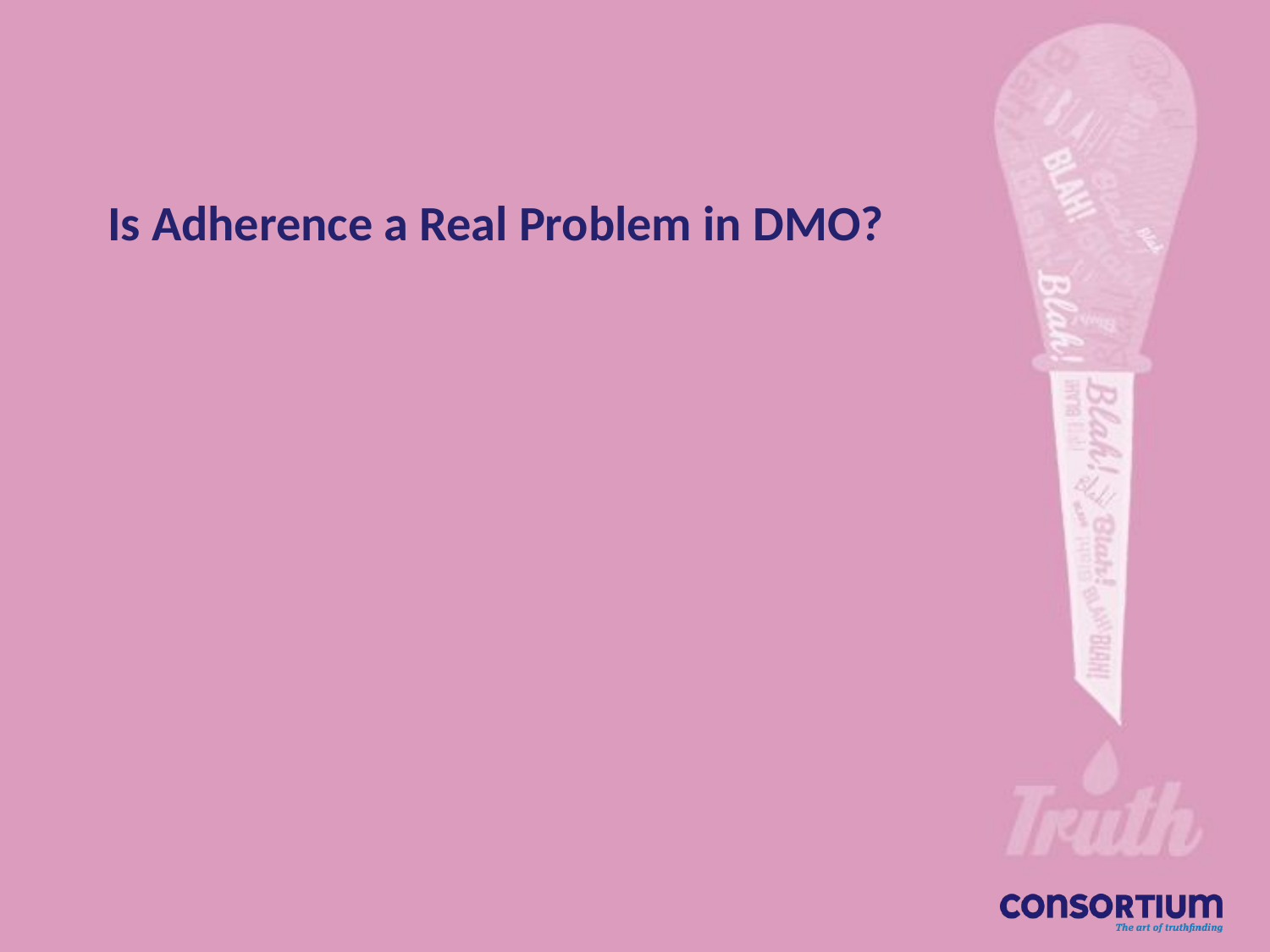

# Is Adherence a Real Problem in DMO?

## Slide 46
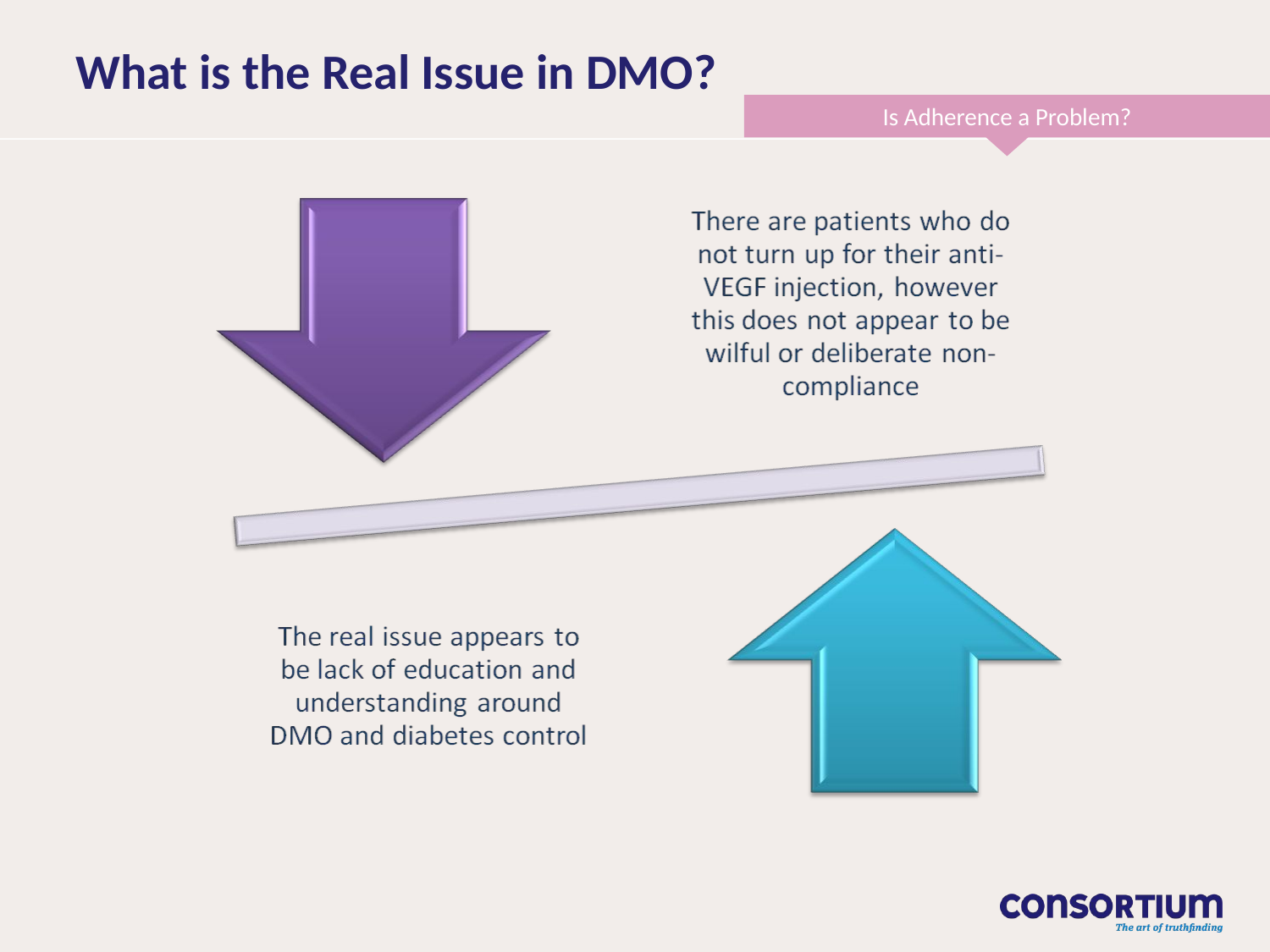

# What is the Real Issue in DMO?
Is Adherence a Problem?

## Slide 47
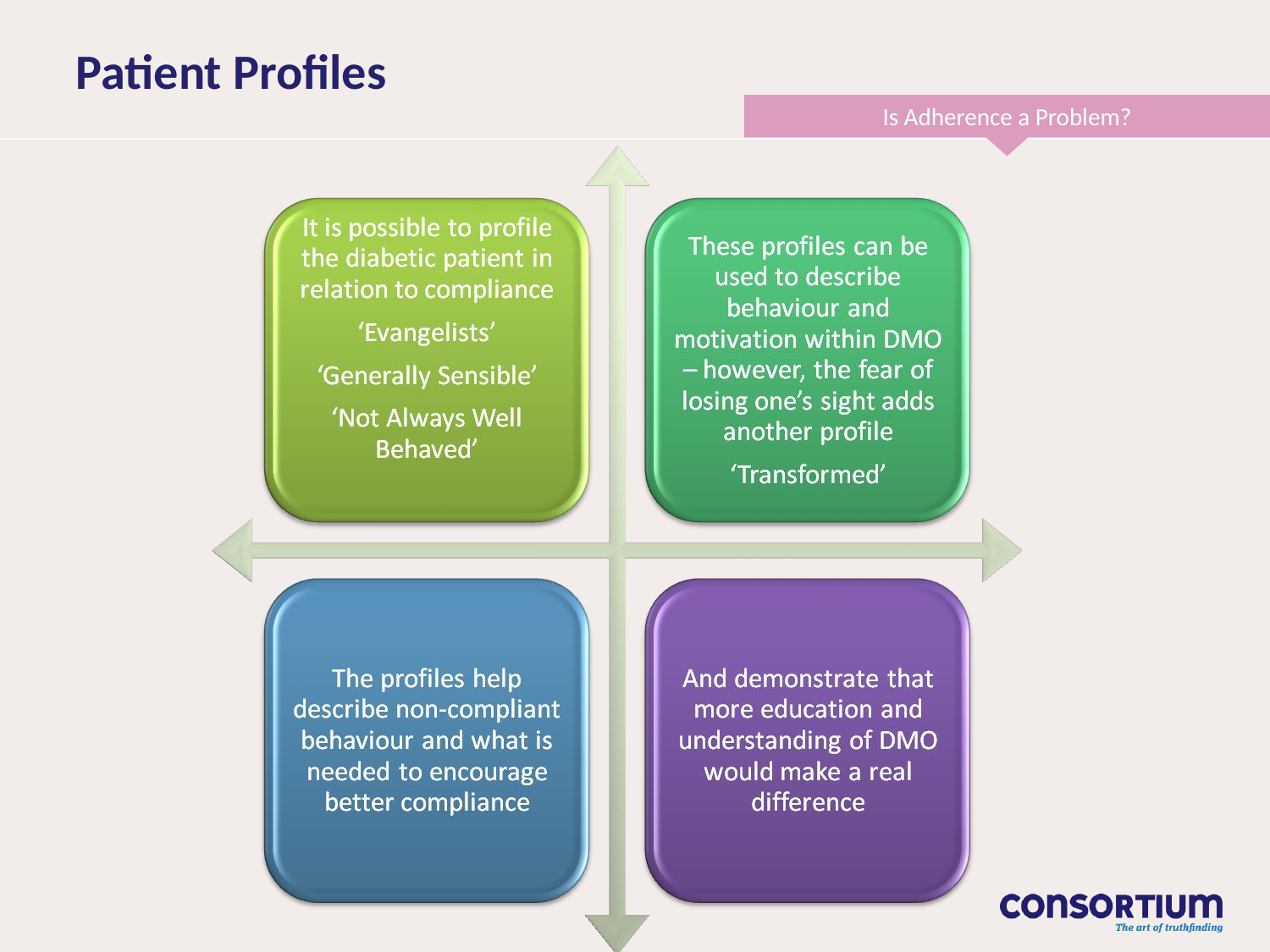

# Patient Profiles
Is Adherence a Problem?

## Slide 48
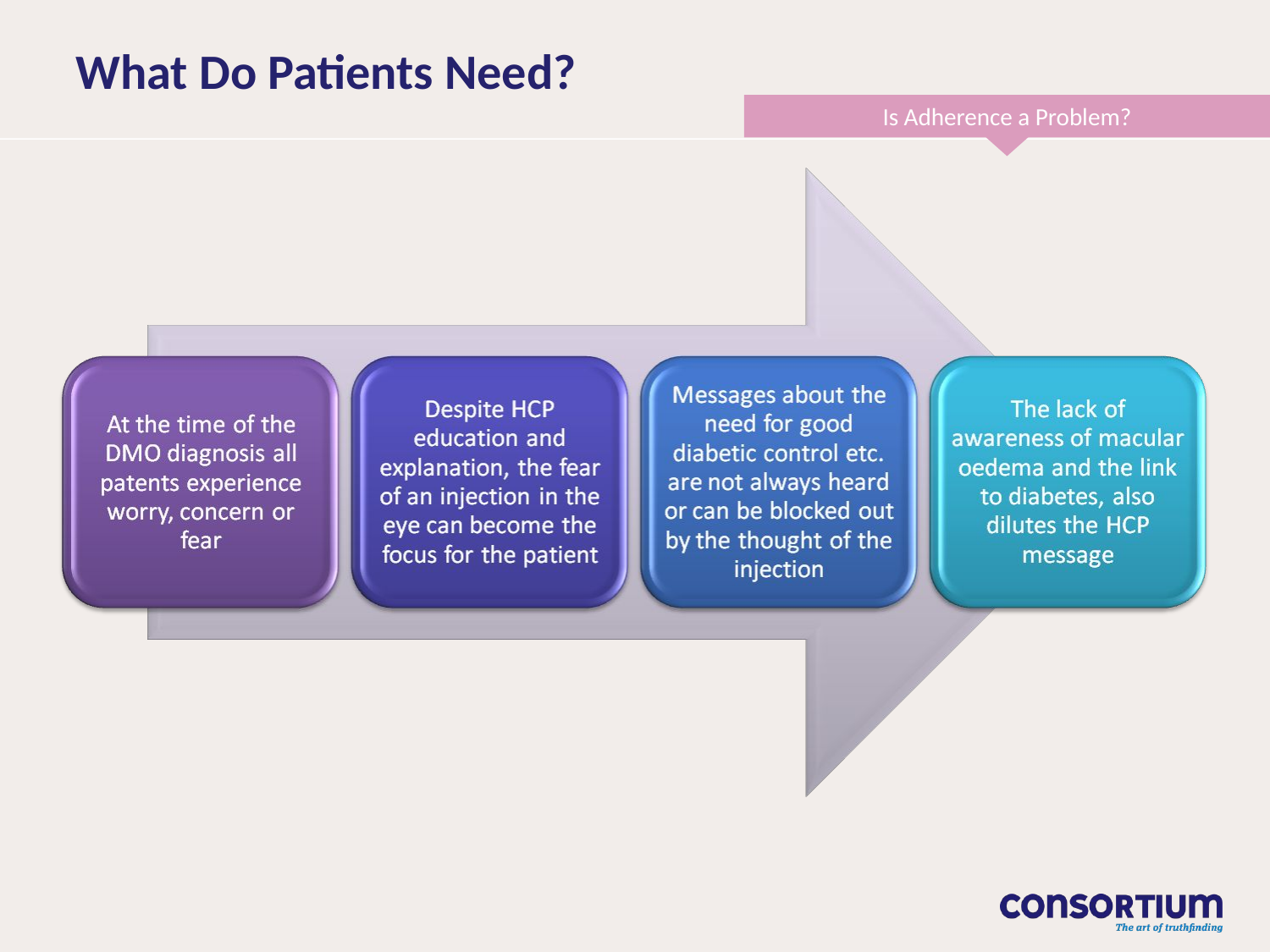

# What Do Patients Need?
Is Adherence a Problem?

## Slide 49
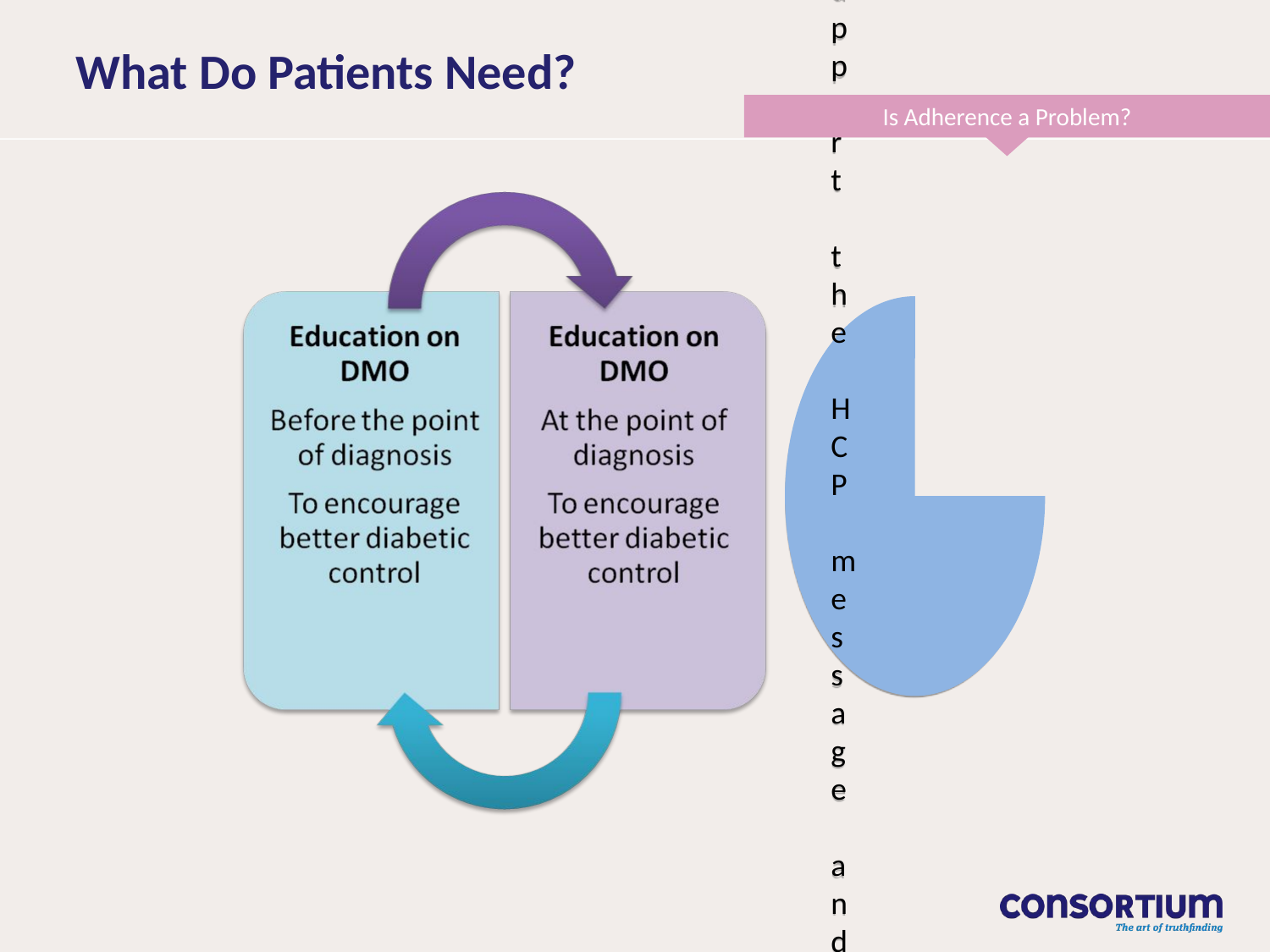

# What Do Patients Need?
Is Adherence a Problem?
An Educational Vehicle
To support the HCP message and agenda
And motivate the patient

## Slide 50
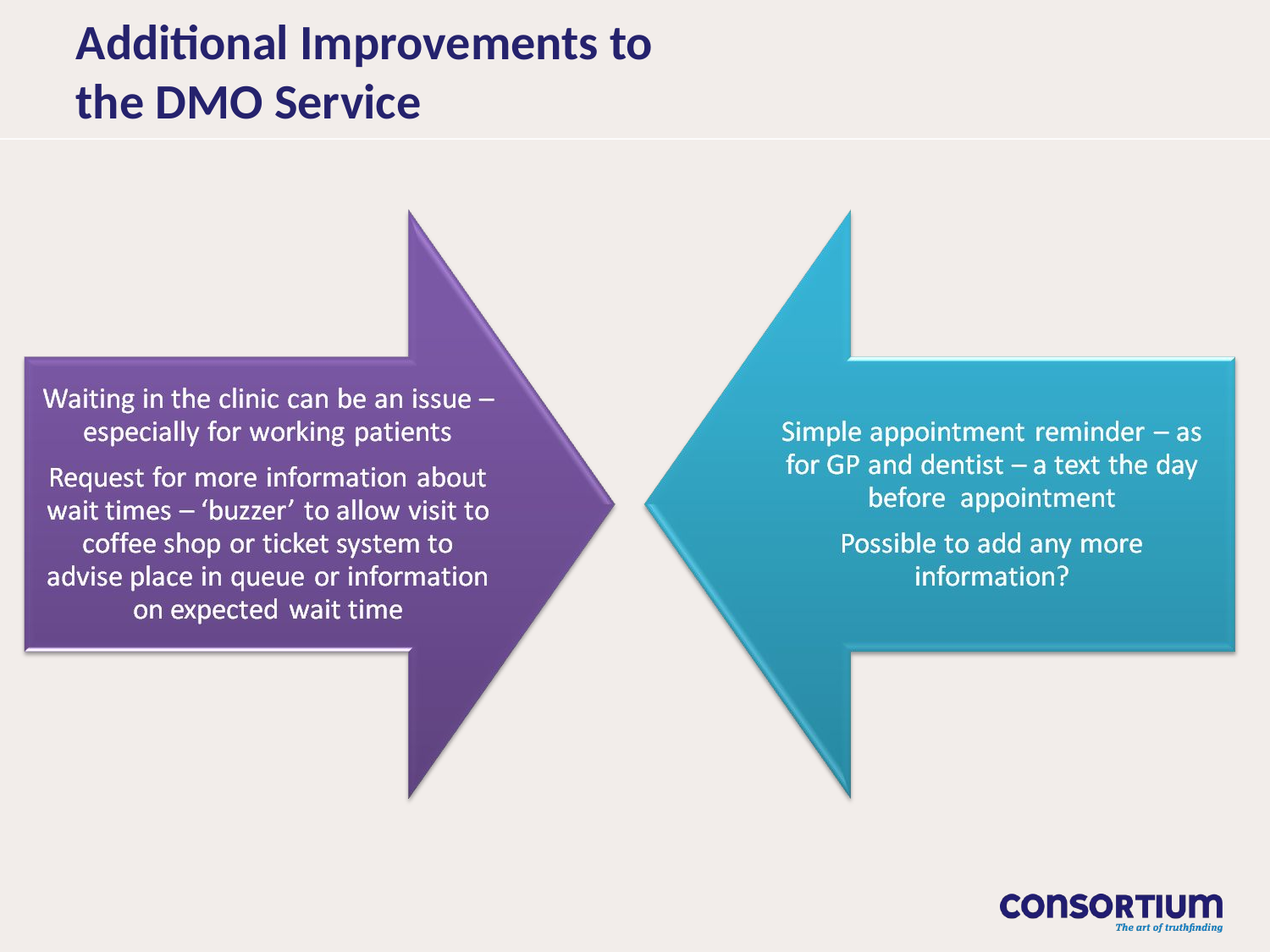

# Additional Improvements to the DMO Service

## Slide 51
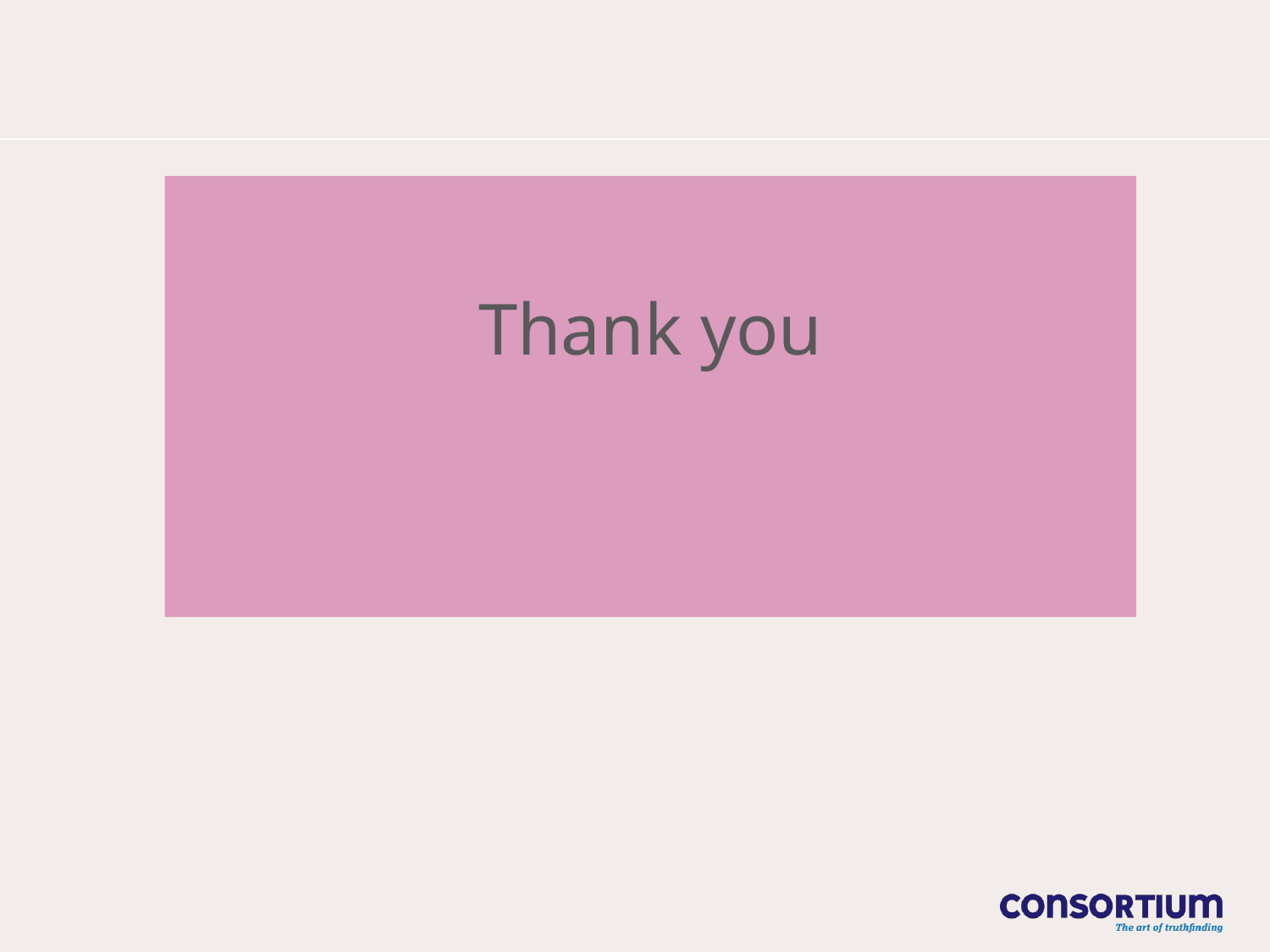

Thank you
